# Supplementary material for: Deep physical neural networks trained with backpropagation
Source: Nature. 2022 Jan 26;601(7894):549–55. doi: 10.1038/s41586-021-04223-6 (PMC8791835; doi:10.1038/s41586-021-04223-6)
Supplement: Supplementary file 1 — This file contains Supplementary Sections 1–5, including Supplementary Figs. 1–37 and References—see the contents page for details. [file 41586_2021_4223_MOESM1_ESM.pdf]

nature portfolio

https://doi.org/10.1038/s41586-021-04223-6

Supplementary information

Deep physical neural networks trained with backpropagation

In the format provided by the
authors and unedited

Deep physical neural networks trained with backpropagation: supplementary material

CONTENTS

1. Physics-aware training 2
   A. Intuition for why physics-aware training works 2
   B. General formulation of physics-aware training 4
   C. Motivating physics-aware training with multilayer feedforward architecture 7
   D. Numerical example 12

2. Supplementary methods 18
   A. Ultrafast second-harmonic generation PNN 18
      1. Experimental setup 18
      2. Input-output transformation characterization 19
   B. Analog transistor PNN 20
      1. Experimental setup 20
      2. Input-output transformation characterization 21
   C. Oscillating plate PNN 22
      1. Experimental setup 22
      2. Input and output encoding 22
      3. Characterization of input-output transformation 24
   D. Data-driven differentiable models 25
      1. Digital model for the mean 25
      2. Digital model for the noise 26
   E. Descriptions of PNN architectures 29
      1. Figure 2-3: Femtosecond SHG-vowels PNN 29
      2. Figure 4: Analog electronic MNIST handwritten digit image classification PNN 34
      3. Figure 4: Oscillating plate MNIST handwritten digit image classification PNN 37
      4. Figure 4: Femtosecond SHG MNIST handwritten digit image classification PNN 39

3. Comparison with reservoir computing 43

4. Scaling PNNs and PAT to more challenging tasks: a numerical example with nonlinear oscillators 47
   A. Example system 47
   B. PNN architecture and details of the physical system 48
   C. MNIST performance 49
   D. Fashion MNIST performance 50
   E. Device-to-device transfer of parameters learned with PAT 51

5. Considerations for useful physical neural networks based on unconventional physical substrates 54
   A. A plausible design workflow for developing PNNs based on unconventional physical substrates 54
   B. Simulation analysis as a tool for physical neural network design 55
      1. General case 56
      2. Nonlinear optical pulse propagation in one dimension 58

3. Analog transistor networks and similar systems 61
C. The operational bottleneck 64
D. PNN design considerations for practical benefits 66
1. Parameter storage and interlayer connectivity 66
2. Pre-training PNNs using *in silico* training 67
3. PNNs for physical-domain inputs and outputs 67

References 70

1. PHYSICS-AWARE TRAINING

In this section, we describe physics-aware training (PAT) and explain its effectiveness at training physical neural networks (PNNs). In Sec. 1 B, we develop the algorithm in full mathematical detail, comparing it to the conventional backpropagation algorithm. In Sec. 1 C, this general case is specified to a generic multi-layer physical neural network. In that section, we introduce and compare physics-aware training to the most relevant alternative for training sequences of physical systems with backpropagation, *in silico* training, in which the backpropagation algorithm is applied to a simulation of the physical system(s). Finally, in Sec. 1 D, we present a numerical example to demonstrate how PAT efficiently trains PNNs, by training a simulated feedforward PNN to perform the vowel classification task with the different training algorithms introduced in this section.

Before delving into more detailed subsections, however, the next subsection provides a high-level intuition for why physics-aware training works, and why alternatives fail. This intuition is backed up rigorously in subsequent subsections.

A. Intuition for why physics-aware training works

The backpropagation algorithm solves the problem of efficiently training the functionality of sequences of mathematical operations with many trainable parameters to perform desired mathematical functions. Its key ingredient is the analytic differentiation of the mathematical operations to efficiently compute the gradient, and hence to determine the optimal parameter updates to improve performance.

Physics-aware training (PAT) solves the problem of applying the backpropagation algorithms to train sequences of real physical operations to perform desired physical functions – which includes performing physically-implemented computations, i.e. physical neural networks.

There are two natural alternatives to PAT. The backpropagation algorithm cannot be applied directly – real physical systems cannot be analytically differentiated. Instead, one could estimate derivatives by using a finite-difference estimator. But this requires n uses of the physical system, where n is the number of trainable parameters. Since n is often 10^6 or even 10^9, and keeps increasing as deep neural network models are scaled up, finite-difference just isn't a practical solution, even for very fast physical systems. The other alternative is to train the physical system using numerical simulations, which are ultimately made up of mathematical operations that one can apply the backpropagation algorithm to. The problem with this approach, however, is that a simulation is not reality – even the most finely-tuned simulation will only describe reality to within some finite precision. A simulation-reality gap is a well-known problem in robotics [24, 72]; the problem for PNNs is similar. The existence of this inevitable simulation-reality gap leads to two problems.

The first problem is the build-up of the simulation-reality gap through layers. Even if the simulation-reality gap is very small (i.e., the model is very accurate), it can matter when we have a process that is composed of a sequence of physical layers, where each layer after the first one takes in the output of the previous layer as an input. Basic error propagation shows us that a small simulation-reality gap (i.e., a small difference between reality and the simulation of reality) is going to build up, usually very rapidly, through layers.

This is a very simple problem, so simple that it can be easily misunderstood. Consider a toy example. Suppose we have a function $f(x) = 2x^{1.1}$ (the 'reality') and a very good 'model' for that function $f_m(x) = 2.01x^{1.1}$. If we take a composition of $f(x)$, $f(f(f(f(f(\dots f(x)))))) = f^n(x)$, as occurs in a deep network or general hierarchical process, the small inaccuracies of the model blow-up. For $n = 1$, the simulation-reality gap (the relative error in the model's output compared to the reality) is about 0.5%. For $n = 5$, it has grown to 3%. By $n = 20$, the gap is 30%. Of course, this is only a toy example: the blow-up depends, among other things, on the function, where it is evaluated, and how and where the model is inaccurate. The key point is that the model is increasingly wrong as the depth increases.

The second problem is even bigger – the build-up of the simulation-reality gap through training. Training is a sequential process that involves 10^5, or even many more steps, each of which depends on the previous one. Each step of training results in new parameters, $\theta_i = T(\theta_{i-1}, f)$, where T is the function(al) that performs one training step and θ_i is the parameter vector on the ith training step. The final parameters at training step n are thus a composition of many applications of T, $\theta_n = T^n(\theta_0, f)$. Just as the simulation-reality gap grows with the number of layers, so too does it grow with training steps. Since there are usually many more training steps than layers, and because each training step is a function of the full-depth network (so it is fully affected by the first problem), this second problem is the most significant, and means that even extremely accurate models that can predict deep networks well, are still not good enough to facilitate training of real physical systems.

Physics-aware training mitigates these problems. It solves the first problem, the depth problem, by simply using the true $f(x)$. The second problem, the training-step problem, is mitigated by PAT both because the simulation-reality gap for $f(x)$ is zero, and because the parameter updates are estimated more accurately than if backpropagation were just applied directly to the model. This second point is more subtle and will be elaborated on in rigorous detail in subsequent sections. An approximate intuition, however, is that PAT assures that when we adjust parameters to change the output of a sequence of physical operations, we are accurately estimating which *direction* the output needs to change to improve its true performance. In contrast, when just performing backpropagation on a model, we may inadvertently think that the network is overshooting the desired output, when in fact, if the simulation's parameters were applied to the true physical system, the output would be undershooting. A second aspect of this improved parameter update estimation is that, even though PAT uses a model to estimate gradients, it uses that model at the correct locations for each layer. For example, in the toy example above, if one is performing backpropagation on the model, the location at which derivatives of the model are evaluated is off by only 0.5% for the second layer (which receives the first layer's output as an input), but is off by 30% for the 21st layer. With PAT, the gradients are always estimated assuming the correct inputs. The net effect is that, with PAT, the simulation-reality gap grows much more slowly than if training were performed solely with simulations. Since PAT is always grounded in the true loss, training can never become misled about how well the physical computation is performing. As a result, even if a small simulation-reality gap may still develop with PAT, it still generally finds parameters that lead to good performance, even if those parameters are sometimes different from those that would have been obtained if ideal backpropagation could be applied to the physical system.

In sum, PAT corrects backpropagation of physical systems, compared to performing backpropagation exclusively with a model, by preventing the build-up of a simulation-reality gap in several different ways. First, it exactly calculates the output of a sequence of physical operations, as well as each intermediate output in the sequence, because it uses the actual physical system to do this. Second, it improves the estimation of parameter updates based on gradient descent, because the deviation of the true physical output from the intended output is exactly known,

and because gradients are always estimated with respect to the correct inputs.

PAT seems to do many other things that are helpful, that are mostly beyond the scope of this work, but that are worth mentioning briefly here. These other helpful things include: making sure the trained model works despite the existence of noise in the forward pass, allowing generic models to be used even when there are device-device variations, and allowing training to proceed accurately even when the model fails to account for parts of the true physics. The first occurs because PAT includes a perfect model of the physical system's noise, inherently (the physical system itself). During training, if the physical system's output randomly fluctuates, estimated parameter updates will also fluctuate. Training's progress along 'noisy' dimensions (i.e., dimensions for which the loss strongly fluctuates due to noise) in parameter space will therefore be slowed depending on how noisy those dimensions are. This is essentially a generalization of how quantization-aware training [48] facilitates the training of deep neural networks that can tolerate low-precision weights and low-precision operations. PAT allows generic models to be used on heterogeneous devices because it does not require a perfect model for the physical system (though it does require access to the true physical system). This may be helpful when training mass-manufactured devices whose individual parameters may vary. Lastly, when the model is not accurate in particular regimes, PAT seems to see this as noise, since the estimated parameter updates will average to approximately zero along 'mis-modelled' directions, due to the fluctuating disagreement between the physics and model. This is probably a generalization of the phenomenon of feedback alignment encountered when training deep neural networks with random synaptic feedback [52].

B. General formulation of physics-aware training

Physics-aware training (PAT) is a gradient-based learning algorithm. The algorithm computes the gradients of the loss w.r.t. the parameters of the network. Since the loss indicates how well the network is performing at its machine learning task, the gradients of the loss are subsequently used to update the parameters of the network. The gradients are computed efficiently via the backpropagation algorithm, which we briefly review below.

The backpropagation algorithm is commonly applied to neural networks composed of differentiable functions. It involves two key steps: a forward pass to compute the loss and a backward pass to compute the gradients with respect to the loss. The mathematical technique underpinning this algorithm is reverse-mode automatic differentiation (autodiff). Here, each differentiable function in the network is an autodiff function which specifies how signals propagate forward through the network and how error signals propagate backward. Given the constituent autodiff functions and a prescription for how these different functions are connected to each other, i.e., the network architecture, reverse-mode autodiff is able to compute the desired gradients iteratively from the outputs towards the inputs and parameters (heuristically termed "backward") in an efficient manner.

For example, the output of a conventional deep neural network is given by $f(f(\dots f(f(\boldsymbol{x}, \boldsymbol{\theta}^{[1]}), \boldsymbol{\theta}^{[2]}) \dots, \boldsymbol{\theta}^{[N-1]}), \boldsymbol{\theta}^{[N]})$. f denotes the constituent autodiff function and is given by $f(\boldsymbol{x}, \boldsymbol{\theta}) = \mathrm{Relu}(W\boldsymbol{x} + \boldsymbol{b})$, where the weight matrix $\boldsymbol{W}$ and the bias $\boldsymbol{b}$ are the parameters of a given layer, and Relu is the rectifying linear unit activation function (other activation functions may be used). Given a prescription for how the forward and backward pass is performed for f, the autograd algorithm is able to compute the overall loss of the network. In Sec. 1 C, the full training procedure for simple feedforward NN of this kind is described in more detail.

The conventional backpropagation algorithm uses the same function for the forward and backward passes (see Fig. S1). Mathematically, the forward pass, which maps the input and parameters into the output, is given by

$$\boldsymbol{y} = f(\boldsymbol{x}, \boldsymbol{\theta}), \tag{1}$$

where $\boldsymbol{x} \in \mathbb{R}^n$ is the input, $\boldsymbol{\theta} \in \mathbb{R}^p$ are the parameters, $\boldsymbol{y} \in \mathbb{R}^m$ is the output of the map, and $f : \mathbb{R}^n \times \mathbb{R}^p \to \mathbb{R}^m$ represents some general function that is an constituent operation which is applied in the overall neural network.

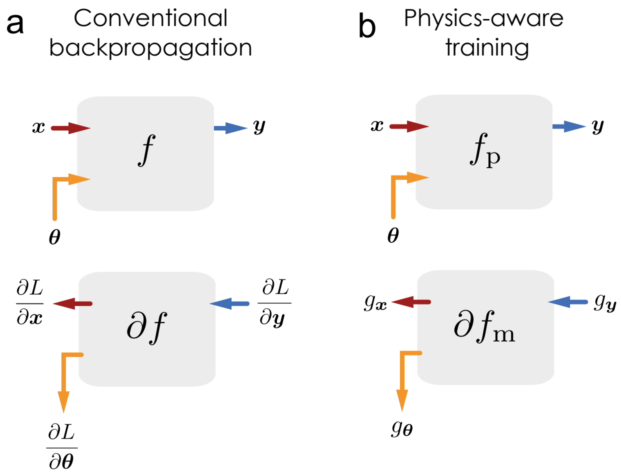

Fig. S1: Schematic for the autodiff function in conventional backpropagation (a) and in physics-aware training (b). In the forward pass, input and parameters are propagated to the output. In the backward pass, gradients w.r.t. the output are backpropagated to gradients w.r.t. the input and parameters. In physics-aware training, the backward pass is estimated using a differentiable digital model, $f_{\mathrm{m}}(\boldsymbol{x}, \boldsymbol{\theta})$, while the forward pass is implemented by the physical system, $f_{\mathrm{p}}(\boldsymbol{x}, \boldsymbol{\theta})$.

The backward pass, which maps the gradients w.r.t. the output into gradients w.r.t. the input and parameters, is given by the following Jacobian-vector product:

$$\frac{\partial L}{\partial \boldsymbol{x}} = \left(\frac{\partial f}{\partial \boldsymbol{x}}(\boldsymbol{x}, \boldsymbol{\theta})\right)^{\mathrm{T}} \frac{\partial L}{\partial \boldsymbol{y}}, \tag{2}$$

$$\frac{\partial L}{\partial \boldsymbol{\theta}} = \left(\frac{\partial f}{\partial \boldsymbol{\theta}}(\boldsymbol{x}, \boldsymbol{\theta})\right)^{\mathrm{T}} \frac{\partial L}{\partial \boldsymbol{y}}, \tag{3}$$

where $\frac{\partial L}{\partial \boldsymbol{y}} \in \mathbb{R}^m$, $\frac{\partial L}{\partial \boldsymbol{x}} \in \mathbb{R}^n$, $\frac{\partial L}{\partial \boldsymbol{\theta}} \in \mathbb{R}^p$ are the gradients of the loss w.r.t. the output, input and parameters respectively. $\frac{\partial f}{\partial \boldsymbol{x}}(\boldsymbol{x}, \boldsymbol{\theta}) \in \mathbb{R}^{m\times n}$ denotes the Jacobian matrix of the function f w.r.t. $\boldsymbol{x}$ evaluated at $(\boldsymbol{x}, \boldsymbol{\theta})$, i.e. $\left(\frac{\partial f}{\partial \boldsymbol{x}}(\boldsymbol{x}, \boldsymbol{\theta})\right)_{ij} = \frac{\partial f_i}{\partial x_j}(\boldsymbol{x}, \boldsymbol{\theta})$. Analogously, $\left(\frac{\partial f}{\partial \boldsymbol{\theta}}(\boldsymbol{x}, \boldsymbol{\theta})\right)_{ij} = \frac{\partial f_i}{\partial \theta_j}(\boldsymbol{x}, \boldsymbol{\theta})$.

Though the backpropagation algorithm given by (1-3) works well for deep learning with digital computers - it cannot be naively applied to physical neural networks. This is because the analytic gradients of input-output transformations performed by physical systems (such as $\frac{\partial f}{\partial \boldsymbol{x}}(\boldsymbol{x}, \boldsymbol{\theta})$) cannot be computed. The derivatives could be estimated via a finite-difference approach - requiring $n + p$ number of calls to the physical system per backward pass. The resulting algorithm would be $n + p$ times slower - this is significant for reasonably sized networks with thousands or millions of trainable parameters.

In physics-aware training, we propose an alternative implementation of the backpropagation algorithm that is suitable for arbitrary real physical input-output transformations. This variant employs autodiff functions that utilizes different functions for the forward and backward pass. As shown schematically in Fig. S1b, we use the non-differentiable transformation of the physical system (f_{p}) in the forward pass and use the differentiable digital model of the physical transformation (f_{m}) in the backward pass. Since physics-aware training is a backpropagation algorithm, which efficiently computes gradients in an iterative manner, it is able to train the physical system efficiently. Moreover, as it is formulated in the same paradigm of reverse-mode automatic differentiation, a user can define these custom autodiff functions in any conventional machine learning library (such as PyTorch) to design and train physical neural networks with the same workflow adopted for conventional neural networks.

Formally, physics-aware training is described as follows. For a constituent physical transformation in the overall

physical neural network, the forward pass operation of this constituent is given by

$$\boldsymbol{y} = f_{\mathrm{p}}(\boldsymbol{x}, \boldsymbol{\theta}). \tag{4}$$

As a different function is used in the backward pass than in the forward pass, the autodiff function is no longer able to backpropagate the gradients at the output layer to the exact gradients at the input layer. Instead, it strives to approximate the backpropagation of the exact gradients. Thus, the backward pass is given by

$$g_{\boldsymbol{x}} = \left(\frac{\partial f_{\mathrm{m}}}{\partial \boldsymbol{x}}(\boldsymbol{x}, \boldsymbol{\theta}) \right)^{\mathrm{T}} g_{\boldsymbol{y}}, \tag{5}$$

$$g_{\boldsymbol{\theta}} = \left(\frac{\partial f_{\mathrm{m}}}{\partial \boldsymbol{\theta}}(\boldsymbol{x}, \boldsymbol{\theta}) \right)^{\mathrm{T}} g_{\boldsymbol{y}}, \tag{6}$$

where $g_{\boldsymbol{y}}$, $g_{\boldsymbol{x}}$, and $g_{\boldsymbol{\theta}}$ are estimators of the gradients $\frac{\partial L}{\partial \boldsymbol{y}}$, $\frac{\partial L}{\partial \boldsymbol{x}}$, and $\frac{\partial L}{\partial \boldsymbol{\theta}}$ respectively.

In listing 1, we show how to construct these custom autograd function for physics-aware training in PyTorch, a popular library for machine learning. With these functions at hand, the user can proceed to build physical neural network with complex architectures in PyTorch. (An implementation of physics-aware training in PyTorch is available at: https://github.com/mcmahon-lab/Physics-Aware-Training.)

```
import torch

def make_pat_func(forward_f, backward_f):
    """
    A function that constructs and returns the custom autograd function for physics-aware training.

    Parameters
    ----------
    f_forward: The function that is applied in the forward pass.
            Typically, the computation is performed on a physical system that is connected and controlled by
            the computer that performs the training loop.
            It takes in an input PyTorch tensor x, a parameter PyTorch tensor theta and returns the output PyTorch tensor y.
    f_backward: The function that is employed in the backward pass to propagate estimators of gradients.
             It takes in an input PyTorch tensor x, an input PyTorch theta and returns the output PyTorch tensor y.

    Returns
    -------
    f_pat: The custom autograd function for physics-aware training.
          It takes in an input PyTorch tensor x, an input PyTorch theta and returns the output PyTorch tensor y.

    """
    class func(torch.autograd.Function):
        @staticmethod
        def forward(ctx, x, theta): #here ctx is an object that stores data for the backward computation.
            ctx.save_for_backward(x, theta) #ctx is used to save the input and parameter of this function.
            return f_forward(x, theta)

        @staticmethod
        def backward(ctx, grad_output):
            x, theta = ctx.saved_tensors #load the input and parameters that are stored in the forward pass
            torch.set_grad_enabled(True) #autograd has to be enabled to perform jacobian computation.
```

```
            # Perform vector jacobian product of the backward function with PyTorch.
            y = torch.autograd.functional.vjp(f_backward, (x, theta), v=grad_output)
            torch.set_grad_enabled(False) #autograd should be restored to off state after jacobian computation.
            return y[1]
    f_pat = func.apply
    return f_pat
```

Listing 1: A helper function that generates the custom autograd function for physics-aware training. This code was designed to run on Python 3.7 and PyTorch 1.6 .

C. Motivating physics-aware training with multilayer feedforward architecture

In the previous subsection, we were able to describe physics-aware training in full generality by casting it in the language of automatic differentiation. Thus, we assumed familiarity with automatic differentiation and did not provide a full prescription of the training loop that is performed during physics-aware training. In this subsection, we pedagogically walk-through all the steps involved in physics-aware training for a specific PNN architecture. Here, we specialize to the feedforward PNN architecture as it is the PNN counterpart of the canonical feedforward neural network (multilayer perceptrons) in deep learning. Similar to Sec. 1 B, we begin by reviewing the full training loop for the conventional backpropagation algorithm. We show why it is ineffective for PNNs, before introducing the alternative training algorithms: *in silico* training and physics-aware training.

From this section onwards, we call the conventional backpropagation algorithm applied to the physical transformations in PNNs the "ideal backpropagation algorithm". This renaming is motivated by two reasons. First, as was pointed out in the last section, physical systems cannot be autodifferentiated, so it is impractical to apply this algorithm to real PNNs. Second, to avoid nomenclature confusion with *in silico* training, which applies the conventional backpropagation algorithm to a model of the system.

In supervised machine learning, a training dataset defined by the set of pairs of examples and targets $\{\, (\boldsymbol{x}^{(k)}, \boldsymbol{y}^{(k)})\,\}_{k=1}^{N_{\text{data}}}$ is provided. During training, the goal is to obtain parameters of the network that maps any given example $\boldsymbol{x}^{(k)}$ to an output (prediction) $\hat{\boldsymbol{y}}^{(k)}$ that is as close as possible to the desired target $\boldsymbol{y}^{(k)}$. In gradient-based learning algorithms, this training is performed by minimizing the following overall loss function L_{total} via gradient descent:

$$L_{\text{overall}} = \frac{1}{N_{\text{data}}} \sum_k L^{(k)} = \frac{1}{N_{\text{data}}} \sum_k \ell(\hat{\boldsymbol{y}}^{(k)}, \boldsymbol{y}^{(k)}), \tag{7}$$

where ℓ is the loss function of choice. For instance, in the case of mean-square error loss, $\ell(\hat{\boldsymbol{y}}, \boldsymbol{y}) = |\hat{\boldsymbol{y}} - \boldsymbol{y}|^2$. As the overall loss function is an average over the different individual examples, the gradient of the loss with respect to parameters of the networks are also averaged over individual examples. Thus, following the approach taken in Ref. [73], the following text will focus on a single example for notational simplicity; we will on occasions present expressions encompassing all examples for completeness.

As shown in Fig. S2, the feedforward PNN is composed of N physical systems. A given example $\boldsymbol{x}$ is fed into the input of the physical system at the first layer $\boldsymbol{x}^{[1]} = \boldsymbol{x}$. Subsequent physical systems' input are taken from the output of the previous layer. Thus, the forward pass is mathematically given by the following iterative expression:

$$\text{Perform Forward Pass :} \qquad \boldsymbol{x}^{[l+1]} = \boldsymbol{y}^{[l]} = f_{\text{p}}(\boldsymbol{x}^{[l]}, \boldsymbol{\theta}^{[l]}). \tag{8}$$

In this architecture, the output of the physical system at the final layer is the prediction produced by the PNN $\hat{\boldsymbol{y}} = \boldsymbol{y}^{[N]}$.

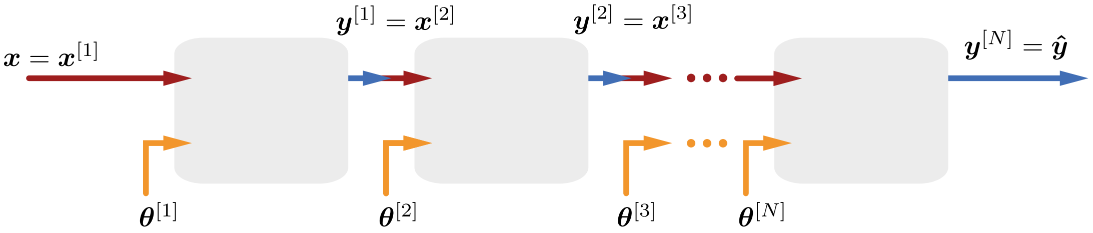

Fig. S2: Schematic of a feedforward physical neural network (PNN). In this architecture, the output of a physical system is passed as an input to the subsequent physical system, i.e. $\boldsymbol{x}^{[l+1]} = \boldsymbol{y}^{[l]}$. Parameters $\boldsymbol{\theta}^{[l]}$ are fed into each layer of the PNN to train the transformation that the PNN applies to the input $\boldsymbol{x}^{[l]}$.

To train the parameters of the PNN via gradient descent, we require the gradient of the loss $L = \ell(\hat{\boldsymbol{y}}, \boldsymbol{y})$ with respect to parameters of each physical system $\frac{\partial L}{\partial \boldsymbol{\theta}^{[l]}}$. These gradients are computed via the backpropagation algorithm, which iteratively computes the gradients from the "back", i.e. from the last layer towards the first layer. This iterative procedure is more efficient than if $\frac{\partial L}{\partial \boldsymbol{\theta}^{[l]}}$ is computed from scratch for each layer, which wastes computation on redundant calculations of many intermediate terms that result from the chain rule. Thus, to begin this process, we first require the error vector ($\frac{\partial L}{\partial \hat{\boldsymbol{y}}} = \frac{\partial L}{\partial \boldsymbol{y}^{[N]}}$), which is computed via

$$\text{Compute Error Vector}: \qquad \frac{\partial L}{\partial \boldsymbol{y}^{[N]}} = \frac{\partial \ell}{\partial \boldsymbol{y}^{[N]}}(\boldsymbol{y}^{[N]}, \boldsymbol{y}). \tag{9}$$

The error vector points out the direction the output of the PNN should change in to minimize the loss. (Since the goal is to *minimize* the loss, in practice one updates parameters along the opposite direction of the gradient.) In the case of the MSE loss, the error vector takes a particularly intuitive form given by the difference between the output and the target ($\frac{\partial L}{\partial \boldsymbol{y}^{[N]}} = 2(\hat{\boldsymbol{y}} - \boldsymbol{y})$). This error vector serves as the "initial" condition for the following backward pass computation:

$$\text{Perform Backward Pass}: \qquad \frac{\partial L}{\partial \boldsymbol{y}^{[l-1]}} = \left(\frac{\partial f_{\mathrm{p}}}{\partial \boldsymbol{x}}(\boldsymbol{x}^{[l]}, \boldsymbol{\theta}^{[l]})\right)^{\mathrm{T}} \frac{\partial L}{\partial \boldsymbol{y}^{[l]}}, \tag{10a}$$

$$\frac{\partial L}{\partial \boldsymbol{\theta}^{[l]}} = \left(\frac{\partial f_{\mathrm{p}}}{\partial \boldsymbol{\theta}}(\boldsymbol{x}^{[l]}, \boldsymbol{\theta}^{[l]})\right)^{\mathrm{T}} \frac{\partial L}{\partial \boldsymbol{y}^{[l]}}. \tag{10b}$$

To gain an intuitive understanding of Eq. 10, it is important to internalize that just like the error vector, the gradients of the loss w.r.t. to the outputs $\frac{\partial L}{\partial \boldsymbol{y}^{[l]}}$ (parameters $\frac{\partial L}{\partial \boldsymbol{\theta}^{[l]}}$) points out how the output (parameters) of each layer should be changed to decrease the loss. Thus, Eq. 10a iteratively prescribes how an output in the previous layer $l-1$ should change (to reduce the loss) given information about how an output in the current layer l should change (to reduce the loss). Eq. 10b then takes this computed information about how outputs should be changed, to compute how the parameters in each layer should be changed to induce that desired change in the output of that layer.

With the gradient of the loss w.r.t. parameters, the parameters can be updated with any gradient-descent optimizer. Though advanced optimizers such as Adam [74] and Adadelta [75] typically results in faster training (and are used in our work), this subsection will focus on the canonical gradient descent update rule (without momentum) for simplicity. In this framework, the parameters are updated as follows:

$$\text{Update parameters}: \qquad \boldsymbol{\theta}^{[l]} \to \boldsymbol{\theta}^{[l]} - \eta \frac{1}{N_{\text{data}}} \sum_k \frac{\partial L^{(k)}}{\partial \boldsymbol{\theta}^{[l]}}, \tag{11}$$

where η is the learning rate and $\frac{\partial L^{(k)}}{\partial \boldsymbol{\theta}^{[l]}}$ denotes the gradient vector computed for the kth example $\boldsymbol{x}^{(k)}$. Consistent with (7), the parameter update involves an average over the different examples.

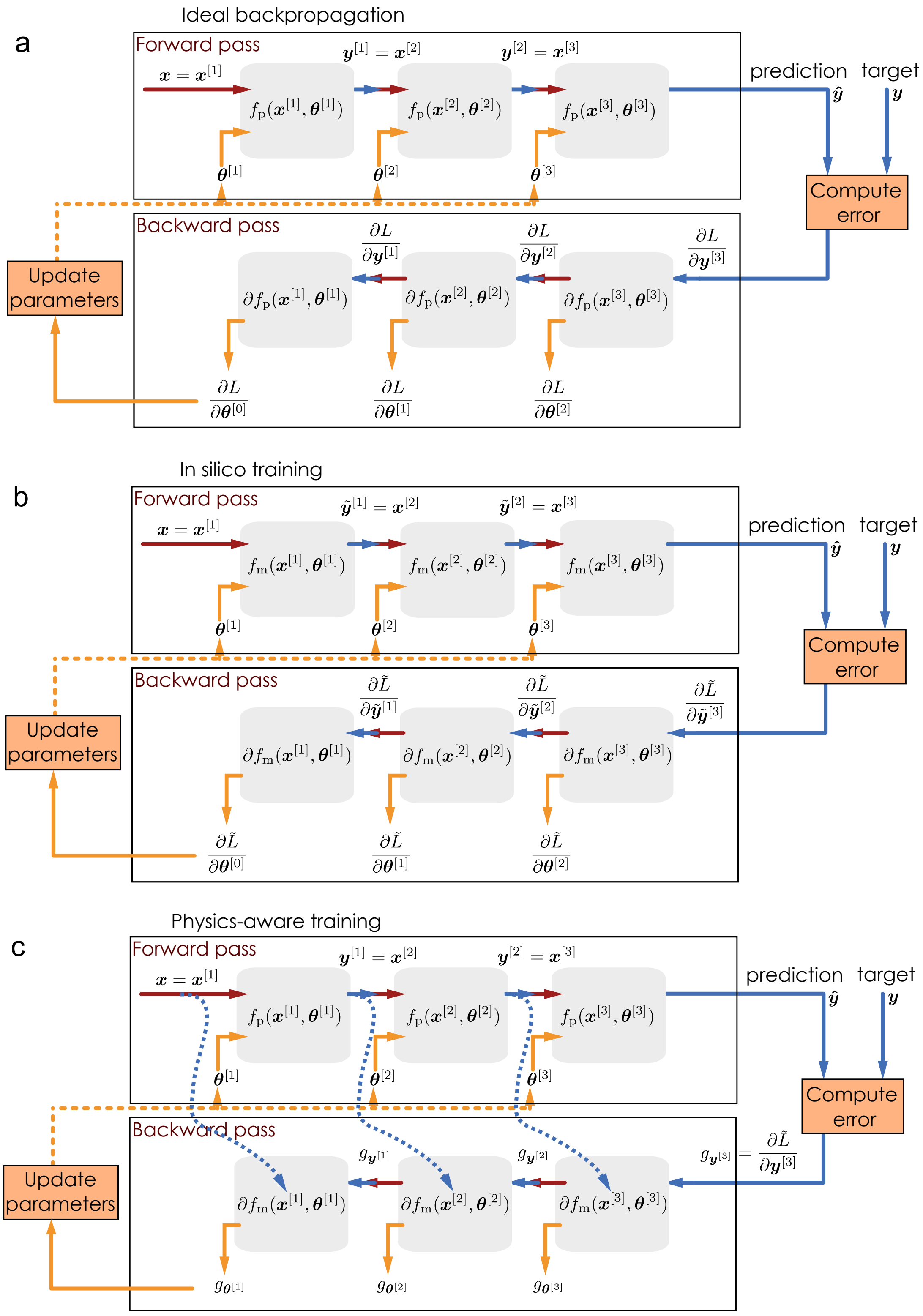

Fig. S3: Schematic of the full training loop for the different training algorithms applied to feedforward PNNs. All three algorithms are backpropagation algorithms and involve four key steps, namely: the forward pass, computing the error vector, the backward pass and the update of the parameters. The main difference between the algorithms is whether the physical transformation f_p or f_m is used for the forward and backward passes. For instance for *in silico* training (panel b), f_m is used in the forward and backward passes. Thus, the intermediate quantities in this algorithm $\tilde{\boldsymbol{x}}^{[l]}$ are different from that of the ideal backpropagation algorithm (panel a) and PAT (panel c). The blue dashed arrows indicate that intermediate outputs are saved during the forward pass and the Jacobians will be evaluated at the saved locations.

Although the ideal backpropagation algorithm (8 - 11) would in principle arrive at good parameters of the physical systems that correspond to low loss, it is not practical in practice as it cannot be performed efficiently. As mentioned in Sec. 1 B, this is because the algorithm requires analytic gradients of the physical transformation f_{p}, which can only be approximated via a finite-difference approach. More specifically, each backward call requires $n+p$ number of repeated calls to the physical system, making the training prohibitively slow for PNNs with large number of parameters.

In silico training circumvents this issue by training the parameters of a network with a differentiable digital model f_{m} of the physical transformation. As the digital model is differentiable, the analytic Jacobian required for backpropagation can be computed in roughly the same time required for the forward pass (without repetition). Hence, *in silico* training could also be referred to as training performed in numerical simulations, or training performed exclusively with a numerical model. In *in silico* training, the training happens solely on a digital computer, to arrive at a set of parameters for the PNNs. After training, these parameters are loaded on to physical system for evaluation. Thus, the algorithm is equivalent to performing conventional backpropagation with f_{m} as the autodiff function. Thus, the full training loop is given by

$$\text{Perform Forward Pass} \quad : \quad \tilde{\boldsymbol{x}}^{[l+1]} = \tilde{\boldsymbol{y}}^{[l]} = f_{\text{m}}(\tilde{\boldsymbol{x}}^{[l]}, \boldsymbol{\theta}^{[l]}), \tag{12}$$

$$\text{Compute Error Vector} \quad : \quad \frac{\partial \tilde{L}}{\partial \tilde{\boldsymbol{y}}^{[N]}} = \frac{\partial \ell}{\partial \tilde{\boldsymbol{y}}^{[N]}}(\tilde{\boldsymbol{y}}^{[N]}, \boldsymbol{y}), \tag{13}$$

$$\text{Perform Backward Pass} : \quad \begin{aligned} \frac{\partial \tilde{L}}{\partial \tilde{\boldsymbol{y}}^{[l-1]}} &= \left(\frac{\partial f_{\text{m}}}{\partial \boldsymbol{x}}(\tilde{\boldsymbol{x}}^{[l]}, \boldsymbol{\theta}^{[l]}) \right)^{\text{T}} \frac{\partial \tilde{L}}{\partial \tilde{\boldsymbol{y}}^{[l]}}, \\ \frac{\partial \tilde{L}}{\partial \boldsymbol{\theta}^{[l]}} &= \left(\frac{\partial f_{\text{m}}}{\partial \boldsymbol{\theta}}(\tilde{\boldsymbol{x}}^{[l]}, \boldsymbol{\theta}^{[l]}) \right)^{\text{T}} \frac{\partial \tilde{L}}{\partial \tilde{\boldsymbol{y}}^{[l]}}, \end{aligned} \tag{14}$$

$$\text{Update parameters} \quad : \quad \boldsymbol{\theta}^{[l]} \to \boldsymbol{\theta}^{[l]} - \eta \frac{1}{N_{\text{data}}} \sum_k \frac{\partial \tilde{L}^{(k)}}{\partial \boldsymbol{\theta}^{[l]}}, \tag{15}$$

where $\tilde{\boldsymbol{x}}^{[l]}, \tilde{\boldsymbol{y}}^{[l]}$ denotes the input and output of each layer that is predicted by the differentiable digital model. With the predicted output of the PNN ($\tilde{\boldsymbol{y}}^{[N]}$), the predicted loss $\tilde{L}$ is computed and minimized in *in silico* training via parameter updates governed by gradients of predicted loss $\tilde{L}$ ($\frac{\partial \tilde{L}}{\partial \boldsymbol{\theta}^{[l]}}$) instead of the true loss L ($\frac{\partial L}{\partial \boldsymbol{\theta}^{[l]}}$). Therefore, the angle between the gradients vectors $\measuredangle(\frac{\partial L}{\partial \boldsymbol{\theta}^{[l]}}, \frac{\partial \tilde{L}}{\partial \boldsymbol{\theta}^{[l]}})$ conceptually characterizes the performance of the *in silico* training algorithm. When this angle is above 90°, training the PNN (via a parameter update) would decrease the predicted loss, but actually increase the true error, and *degrade* the performance of the PNN.

We use the angle between gradients and the gradient obtained by ideal backpropagation as a conceptual tool here, rather than a readily computable metric of performance since the ideal backpropagation algorithm is impractical to implement in experiment. Ultimately, the best metric of training algorithms is their achieved performance. In addition, since there are usually many equivalently-performing local optima in training large neural networks, it is not necessarily an indication that a backpropagation algorithm performs poorly if it does not follow the exact same training trajectory as ideal backpropagation. This angle is only evaluated in Sec. 1 D as we treat a hypothetical numerical example, which allows for the "true" gradient to be computed via autograd. In addition, the gradient vectors of the two differing algorithm must be evaluated for the same parameters and the same PNN input $\boldsymbol{x}$ (example of the ML dataset). Finally, in addition to the angle, the difference between the magnitude of the gradient vectors ($|\frac{\partial L}{\partial \boldsymbol{\theta}^{[l]}}| - |\frac{\partial \tilde{L}}{\partial \boldsymbol{\theta}^{[l]}}|$) is also an important metric as the magnitude of the gradient vector determines how big a step is taken during training. Thus, if the magnitude of the predicted gradient is vastly off, it can lead to training instabilities.

With the angle metric in mind, it becomes apparent that *in silico* training has a decisive flaw; the error vector $\frac{\partial \tilde{L}}{\partial \tilde{\boldsymbol{y}}^{[N]}}$, which is the initial condition of the backward pass, is computed via the predicted output $\tilde{\boldsymbol{y}}^{[N]}$ instead of the true output $\boldsymbol{y}^{[N]}$. Thus, without an exceptionally precise digital model, this predicted error vector would point in a significantly different direction from the true error vector. This inaccurate error vector would then be backpropagated via (14), to result in bad parameter updates. This problem is exacerbated for deep PNNs, as the difference between

the predicted output of the digital model and the physical system scales exponentially w.r.t. to depth. In addition to the inaccurate error vector, the Jacobian matrices that are used to backpropagate the gradients in the intermediate layers are also inaccurate as they evaluated at the inaccurate points, at $\tilde{\boldsymbol{x}}^{[l]}$ instead of $\boldsymbol{x}^{[l]}$.

Physics-aware training is specifically designed to avoid the pitfalls of both the ideal backpropagation algorithm and *in silico* training. It is a hybrid algorithm involving computation in both the physical and digital domain. More specifically, the physical system is used to perform the forward pass, which alleviates the burden of having the differential digital models be exceptionally accurate (as in *in silico* training). The differentiable digital model is only utilized in the backward pass to complement parts of the training loop that the physical system cannot perform.

As described in Section. 1 B, physics-aware training can be formalized by the use of custom constituent autodiff functions in an overall network architecture. In the case of the feedforward PNN, the autodiff algorithm with these custom functions results in and simplifies to the following training loop:

$$\text{Perform Forward Pass} \quad : \quad \boldsymbol{x}^{[l+1]} = \boldsymbol{y}^{[l]} = f_{\mathrm{p}}(\boldsymbol{x}^{[l]}, \boldsymbol{\theta}^{[l]}), \tag{16}$$

$$\text{Compute Error Vector} \quad : \quad g_{\boldsymbol{y}^{[N]}} = \frac{\partial L}{\partial \boldsymbol{y}^{[N]}} = \frac{\partial \ell}{\partial \boldsymbol{y}^{[N]}}(\boldsymbol{y}^{[N]}, \boldsymbol{y}), \tag{17}$$

$$\text{Perform Backward Pass} : \quad \begin{aligned} g_{\boldsymbol{y}^{[l-1]}} &= \left(\frac{\partial f_{\mathrm{m}}}{\partial \boldsymbol{x}}(\boldsymbol{x}^{[l]}, \boldsymbol{\theta}^{[l]})\right)^{\mathrm{T}} g_{\boldsymbol{y}^{[l]}}, \\ g_{\boldsymbol{\theta}^{[l]}} &= \left(\frac{\partial f_{\mathrm{m}}}{\partial \boldsymbol{\theta}}(\boldsymbol{x}^{[l]}, \boldsymbol{\theta}^{[l]})\right)^{\mathrm{T}} g_{\boldsymbol{y}^{[l]}}, \end{aligned} \tag{18}$$

$$\text{Update parameters} \quad : \quad \boldsymbol{\theta}^{[l]} \to \boldsymbol{\theta}^{[l]} - \eta \frac{1}{N_{\text{data}}} \sum_k g^{(k)}_{\boldsymbol{\theta}^{[l]}}, \tag{19}$$

where $g_{\boldsymbol{\theta}^{[l]}}, g_{\boldsymbol{y}^{[l]}}$ are estimators of the "exact" gradients $\frac{\partial L}{\partial \boldsymbol{\theta}^{[l]}}, \frac{\partial L}{\partial \boldsymbol{y}^{[l]}}$ respectively. Thus, analogous to the metric defined for *in silico* training, the angle between the estimated gradient vector and the exact gradient vector $\measuredangle(\frac{\partial L}{\partial \boldsymbol{\theta}^{[l]}}, g_{\boldsymbol{\theta}^{[l]}})$ conceptually characterizes the performance of physics-aware training. In physics-aware training, the error vector is exact ($g_{\boldsymbol{y}^{[N]}} = \frac{\partial L}{\partial \boldsymbol{y}^{[N]}}$) as the forward pass is performed by the physical system. This error vector is then backpropagated via (18), which involves Jacobian matrices of the differential digital model evaluated at the "correct" inputs ($\boldsymbol{x}^{[l]}$ instead of $\tilde{\boldsymbol{x}}^{[l]}$) at each layer. Thus, in addition to utilizing the output of the PNN ($\boldsymbol{y}^{[N]}$) via physical computations in the forward pass, intermediate outputs ($\boldsymbol{y}^{[l]}$) are also utilized to facilitate the computation of accurate gradients in physics-aware training.

D. Numerical example

In this subsection, we present a numerical example to demonstrate how PAT efficiently trains PNNs. The numerical example presented here is a simplified, simulated emulation of the experimental results presented in Figure 2 of the main manuscript. Thus, we train a PNN to perform the vowel classification task with a feedforward mult-layer architecture, where the constituent hypothetical physical transformation is an autocorrelation of the input and parameters (see (21)), which is a crude approximation for the physical transformation realized with broadband optical second harmonic generation. We construct a differentiable digital model for this hypothetical physical transformation, which is then employed in PAT. The performance of PAT is compared against the performance achieved by training with ideal backpropagation and *in silico* training. We show the training curves and how the computation is performed in the forward and backward pass, for the different algorithms.

In the vowel task, the input vowel formant vectors are 12-dimensional, while the output required for predictions is 7-dimensional, since we consider 7 vowel classes and use the one-hot encoding. To accommodate this, we consider the feedforward PNN introduced previously, adding some minor pre-processing and post-processing of the initial input and final output respectively.

More concretely, we consider a feedforward PNN with a constituent physical function f_{p} that has 24-dimensional inputs and outputs. Hence, the input vowel vector is repeated twice to produce a 24-dimensional input for the first layer of the PNN and selected portions of the final output are summed to produce a 7-dimensional vector. Mathematically, the PNN is given by

$$\boldsymbol{x}^{[1]} = [x_1, x_1, x_2, x_2, \ldots, x_{12}, x_{12}]^{\mathrm{T}}, \tag{20a}$$

$$\boldsymbol{x}^{[l+1]} = \boldsymbol{y}^{[l]} = f_{\mathrm{p}}(\boldsymbol{x}^{[l]}, \boldsymbol{\theta}^{[l]}) \qquad \text{for } l = 1 \ldots N, \tag{20b}$$

$$\boldsymbol{o} = [y_5^{[N]} + y_6^{[N]}, y_7^{[N]} + y_8^{[N]}, \ldots, y_{17}^{[N]} + y_{18}^{[N]}]^{\mathrm{T}}. \tag{20c}$$

where $\boldsymbol{o}$ denotes the final output of the PNN [1] and $y_i^{[l]}$ denotes the value of the neuron i (ith index of vector $\boldsymbol{y}^{[l]}$) at layer l. As is typical in conventional NNs, the predicted label for this PNN is given by the index of the final output $\boldsymbol{o}$ that results in the largest value. For the constituent physical function f_{p}, we choose a mathematical form that roughly mirrors the transformation for the SHG experiment conducted in Figure 2 of the main manuscript. Hence, $f_{\mathrm{p}}(\boldsymbol{x}, \boldsymbol{\theta}) = a\boldsymbol{s}/\max(\boldsymbol{s}) + c$, where a and c are trainable factors and offsets. Here $\boldsymbol{s}$ is an autocorrelation of the control signal,

$$s_j = \sum_{i=0}^{\min(j, N-j-1)} q_{j-i} q_{j+i} \tag{21}$$

where $\boldsymbol{q} = \mathrm{clamp}([x_1, \ldots, x_N, \theta_1, \ldots, \theta_N]^T)$ represents the overall control applied to a given layer, which is clamped between 0 and 1 via the clamp operation ($\mathrm{clamp}(v) = \max(0, \min(v, 1))$). With f_{p} at hand, we train a differentiable model f_{m} of the physical transformation via a data driven approach. A deep neural network (DNN) with 3 hidden layers that have 1000, 500, 300 hidden neurons respectively was trained on 2000 instances of physical input vectors (i.e., the vector containing inputs and parameters) and output pairs generated by repeated application of f_{p}.

The PNN is trained by minimizing the following loss function:

$$L = \underbrace{H(\boldsymbol{y}, \hat{\boldsymbol{y}})}_{L_{\text{cross-entropy}}} + \underbrace{\lambda \sum_l \sum_i \max(0, q_i^{[l]} - v_{\max}) - \min(0, q_i^{[l]} - v_{\min})}_{L_{\text{constraint}}} \tag{22}$$

---

[1] Here we denote the output by $\boldsymbol{o}$ instead of $\hat{\boldsymbol{y}}$ as the output of the PNN has not been made to approximate the target $\boldsymbol{y}$ since it has not been converted to a probability distribution via the softmax function.

where H is the cross-entropy function ($H(\boldsymbol{p}, \boldsymbol{q}) = -\sum_i p_i \log(q_i)$) and $\hat{\boldsymbol{y}} = \mathrm{Softmax}(\boldsymbol{o})$. The first term ($L_{\text{cross-entropy}}$) is the cross-entropy loss responsible for training the output of the PNN to approach the desired target, while the second term ($L_{\text{constraint}}$) is a regularization term, with regularization coefficient λ, to constrain the controls $\boldsymbol{q}^{[l]}$ of each layer to be between v_{min} and v_{max}. For this numerical example $\lambda = 0.02$, $v_{\text{min}} = 0$, and $v_{\text{max}} = 1$.

With this loss function, the PNN was trained with the ideal backpropagation algorithm, *in silico* training, and physics-aware training. In Fig. S4, we show the training curves for the 3 training algorithms. Initially, all 3 algorithms are able to train the parameters to reduce the cross-entropy loss (see inset of Fig. S4b). However, by about ∼350 epochs, *in silico* training is no longer able to train the physical system effectively due to the increasing differences between the outputs of f_{p} and f_{m}. PAT initially follows the training curve of the ideal backprop algorithm quite closely. By about ∼ 500 epochs, the effective rate of learning for PAT slows down but nevertheless it is able to lower the loss consistently until the test classification accuracy is ∼96%, which is close to that of ideal backprop. After ∼2000 epochs, the cross-entropy loss and classification accuracies exhibits fluctuations (this is also seen in Figure 2 of the main manuscript). We speculate that minor differences in the Jacobian of f_{p} and f_{m} causes the parameters to drift around when they are located near an optimal parameter region of the overall loss function.

We also characterize the inner workings of the PNNs for the different algorithms. To do so, we consider two perspectives. The first looks at how the algorithms differ over just one training step. This perspective, which is presented in Fig. S5, assumes all algorithms initially have the exact same parameters, and we then take 1 training step (i.e., 1 parameter update based on 1 mini-batch of training examples). The second perspective analyzes divergence of training trajectories over many training steps. This perspective, which is shown in Figs. S6 and S7, shows how the single-step inaccuracy of in silico training compounds through training leading to increasingly worse training of the physical system, and shows how PAT avoids this divergence.

In Fig. S5, we show how the forward and backward passes of the PNN differ across the different algorithms when the parameters are set to initially be the same. Here we observe that the prediction error between the outputs of f_{m} and f_{p} build up progressively through layers during the forward pass. This results in an incorrect error vector for *in silico* training, that is backpropagated to result in incorrect gradients of the loss w.r.t. parameters. As shown in the last row of Fig. S5, the gradients of *in silico* training do not resemble the "true" gradient of the ideal backpropagation

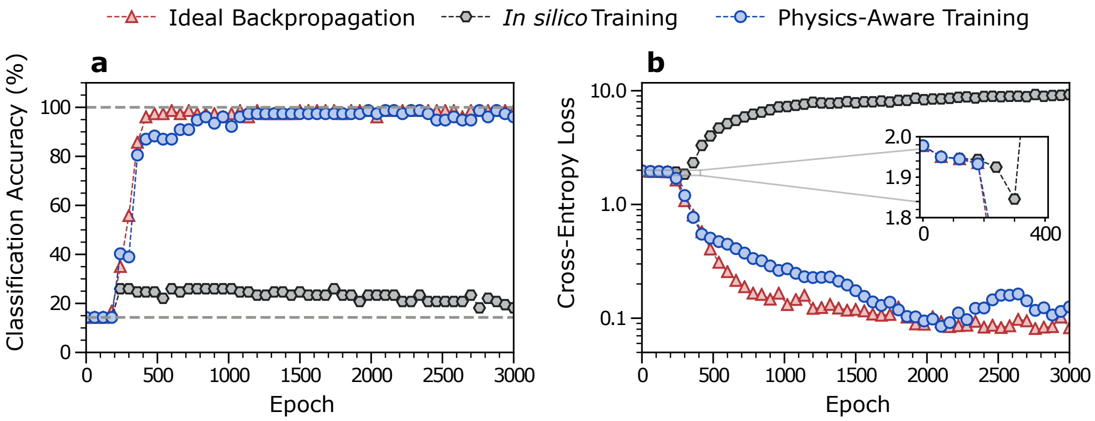

Fig. S4: Comparison of the test classification accuracy (a) and test cross-entropy loss (b) versus training epoch for ideal backpropagation, *in silico* training, and physics-aware training. Since this numerical example attempts to mirror the experiment performed in Figure 2 of the main manuscript, it considers the vowel classification task with a feedforward PNN, specified by (20). The inset in b zooms in on the initial training phase, where the cross-entropy loss for the *in silico* training briefly decreases for about 300 epochs before increasing due to compounding mismatch between f_{p} and f_{m}.

algorithm (which can only be obtained from a finite-difference approach in a laboratory setting), in both direction and magnitude. In contrast, PAT is able to approximate the gradients fairly accurately, as it utilizes the experimental transformation f_{p} in the forward pass. This may be surprising because PAT uses the exact same model as *in silico* training to estimate gradients. The key is that the gradients with PAT are estimated at the correct locations in parameter space (i.e., the inputs to each physical layer are known exactly), whereas in *in silico* training, the gradients are calculated at locations that are increasingly mismatched with reality as one goes deeper into the network.

In Fig. S6 and S7, we characterize the PNNs after training for 300 and 800 epochs respectively, for the different training algorithms. We show how the vowel data propagates through the different layers of the PNNs to arrive at the final prediction $\hat{\boldsymbol{y}}$ for 3 different vowel examples. In Fig. S6, we see that the ideal backpropagation algorithm learns the fastest as it is already able to correct classify 2 of out the 3 vowels. The parameters learned by PAT are also reasonably similar to those obtained with ideal backpropagation, and the trained model performs similarly well. *In silico* training meanwhile trains slowly, but by 300 epochs has made some small positive progress. When the PNN is executed with the parameters obtained by in silico training after 300 epochs, the PNN's output is shifted slightly towards the target vowel. Thus, consistent with Fig. S4, learning has occurred for all 3 algorithms after 300 epochs of training. As training proceeds, however, the simulation-reality mismatch from each training step compounds, causing *in silico* training to diverge rapidly away from ideal backpropagation and PAT. This can be exacerbated because *in silico* training can often proceed into regions of parameter space where its predictions are especially poor, leading to an even faster divergence from ideal backpropagation and PAT. By epoch 800 (see Fig. S7), the parameters predicted by *in silico* training have drifted into a region where the digital model's predictions are especially inaccurate. This is shown in the rightmost column of Fig. S7, where the output of the experiment with parameter trained via *in silico* training classifies the vowel wrongly 2 out of 3 times, while the PAT and ideal backpropagation is able to classify all 3 vowels accurately with a reasonable high confidence, which is indicated by the value of $\hat{\boldsymbol{y}}$ being close to 1 for the correct vowel.

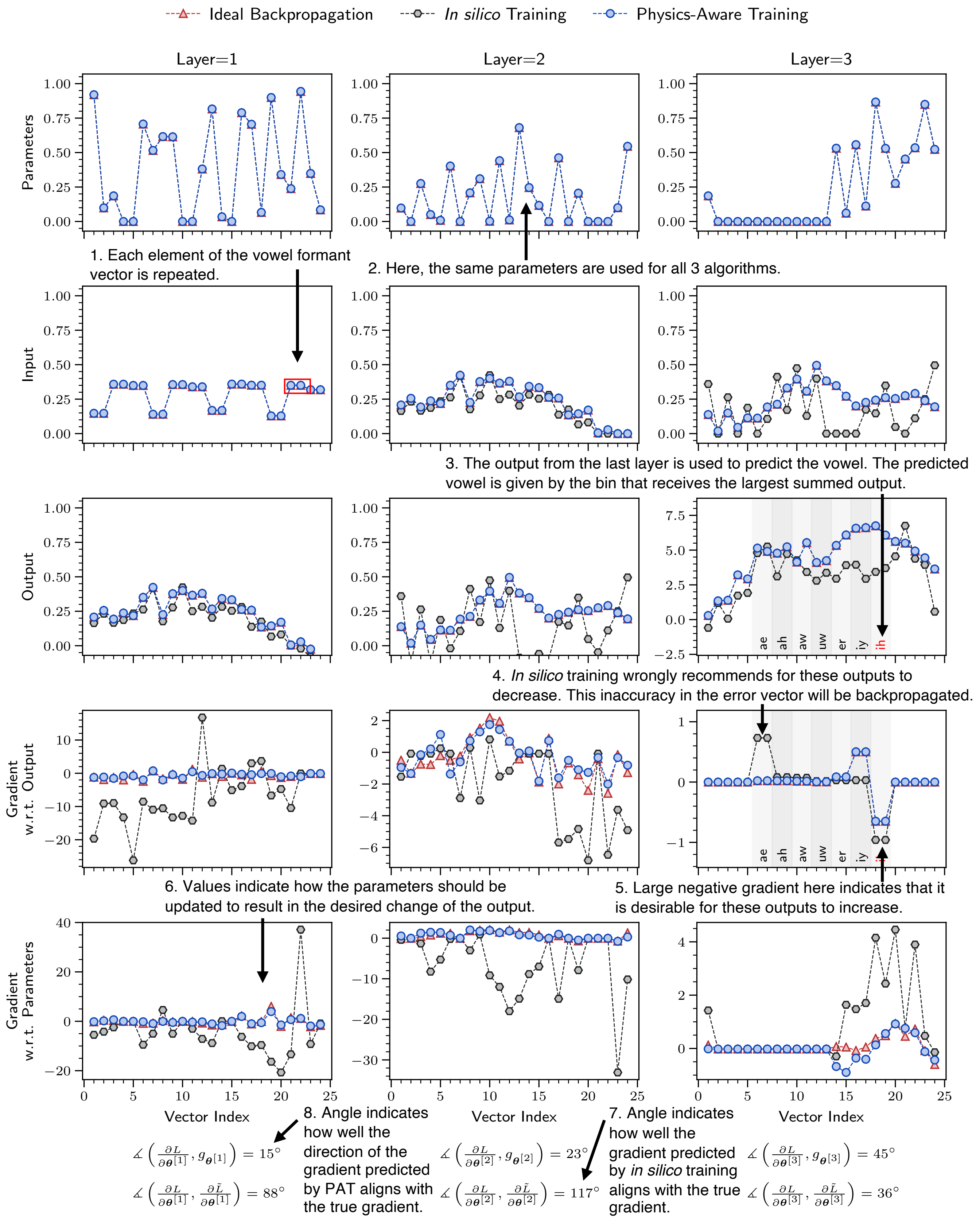

Fig. S5: Comparing 3 training algorithms in a numerical example, for a single training step. More specifically, the inputs, outputs, parameters and gradients of the feedforward PNN applied to the vowel classification task are shown in different rows, and their values for different physical layers in the first, second and third column, respectively. Gradients are evaluated w.r.t. to the parameters and previous layer's output. Here, we transfer the same parameters to all 3 algorithms, to evaluate how similar the gradient vectors are for the different algorithms.

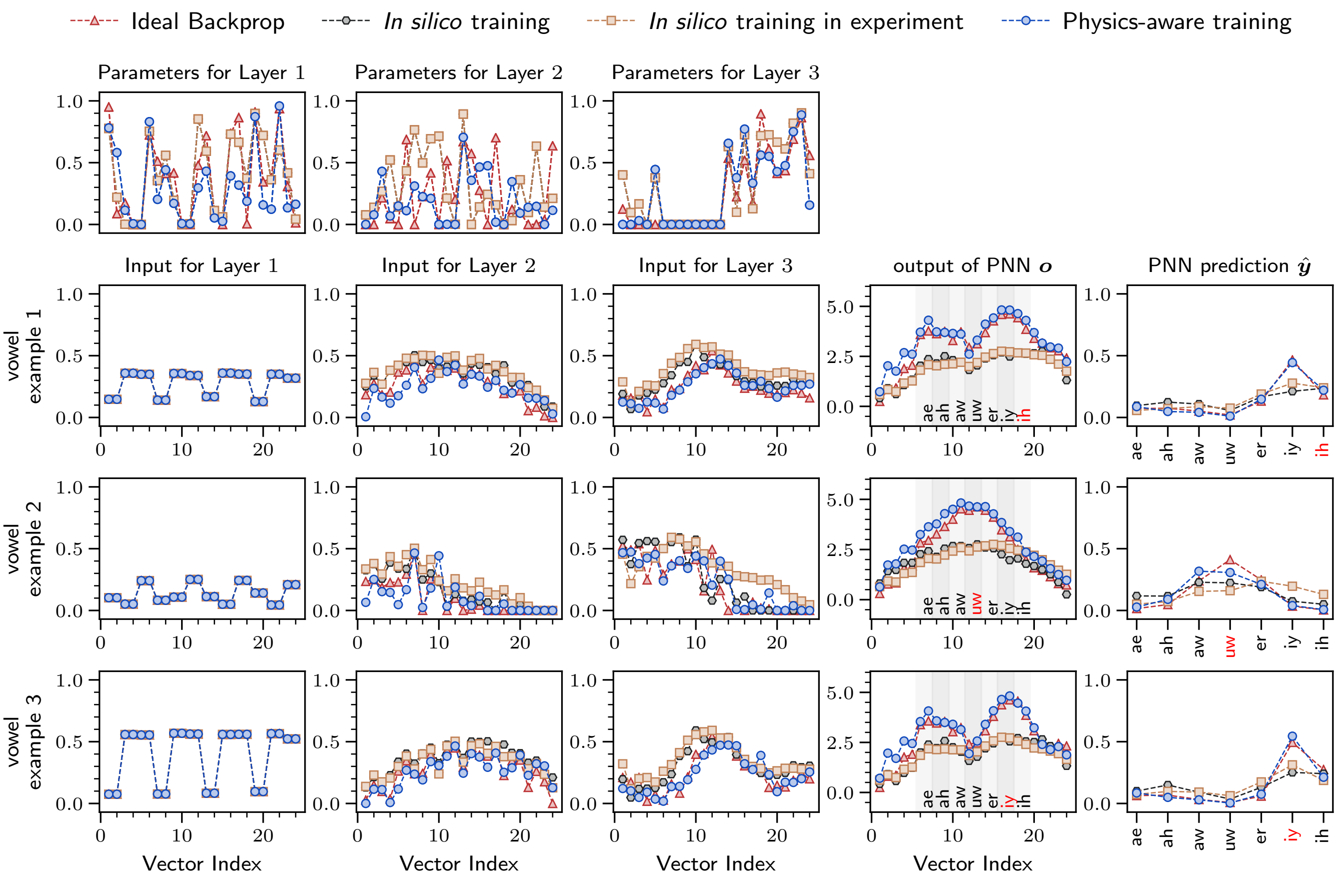

Fig. S6: Comparing the training of 3 training algorithms over many training steps. Here, we show a snapshot of a single step, after the training has proceeded for 300 steps. More specifically, we show the physical inputs at the different layers, the output and the final PNN prediction of PNNs for three example vowels. In contrast to Fig. S5, each algorithm here is associated with different parameters that result from each of their own distinct training trajectories shown in Fig. S4. Plots associated with "*In silico training* in experiments" refer to characterizations of the PNN when the parameters trained via *in silico* training is transferred over and evaluated in the experiment (with the function f_{p} instead of f_{m}).

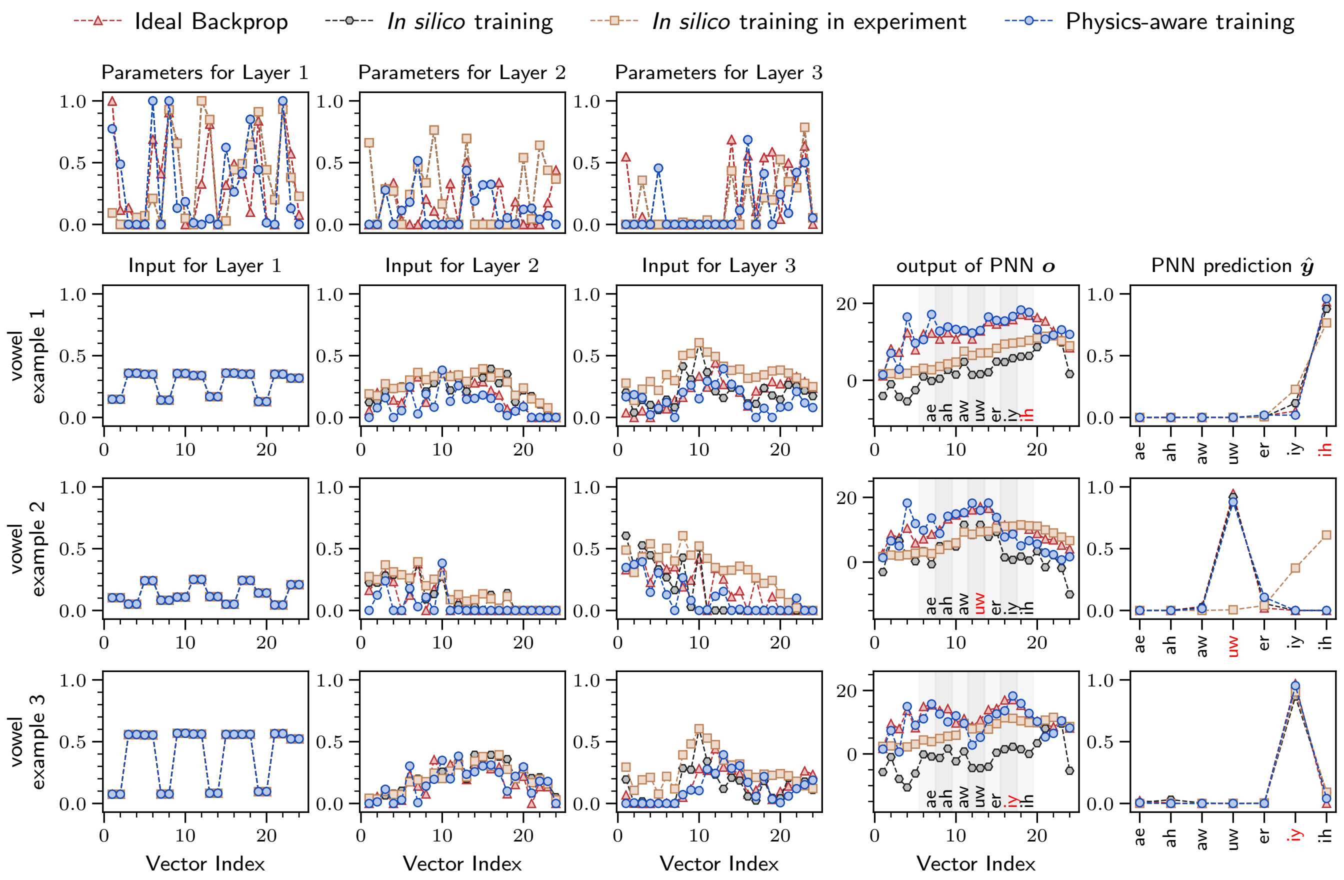

Fig. S7: Comparing the training of 3 training algorithms over many training steps. Here, we show a snapshot of a single step, after the training has proceeded for 800 steps. More specifically, we show the physical inputs at the different layers, the output and the final PNN prediction of PNNs for three example vowels. In contrast to Fig. S5, each algorithm here is associated with different parameters that result from each of their own distinct training trajectories shown in Fig. S4. Plots associated with "*In silico training* in experiments" refer to characterizations of the PNN when the parameters trained via *in silico* training is transferred over and evaluated in the experiment (with the function f_{p} instead of f_{m}).

2. SUPPLEMENTARY METHODS

In this section, we first describe the experimental setups for each of the three physical systems used to implement PNNs. We then describe our data-driven differentiable models, as well as the noise models used to improve the performance of *in silico* training. Finally, the PNN architectures used in the main text for classifying vowels and MNIST digits are described and typical results are presented to illustrate how the PNNs perform the learned classification tasks.

A. Ultrafast second-harmonic generation PNN

1. Experimental setup

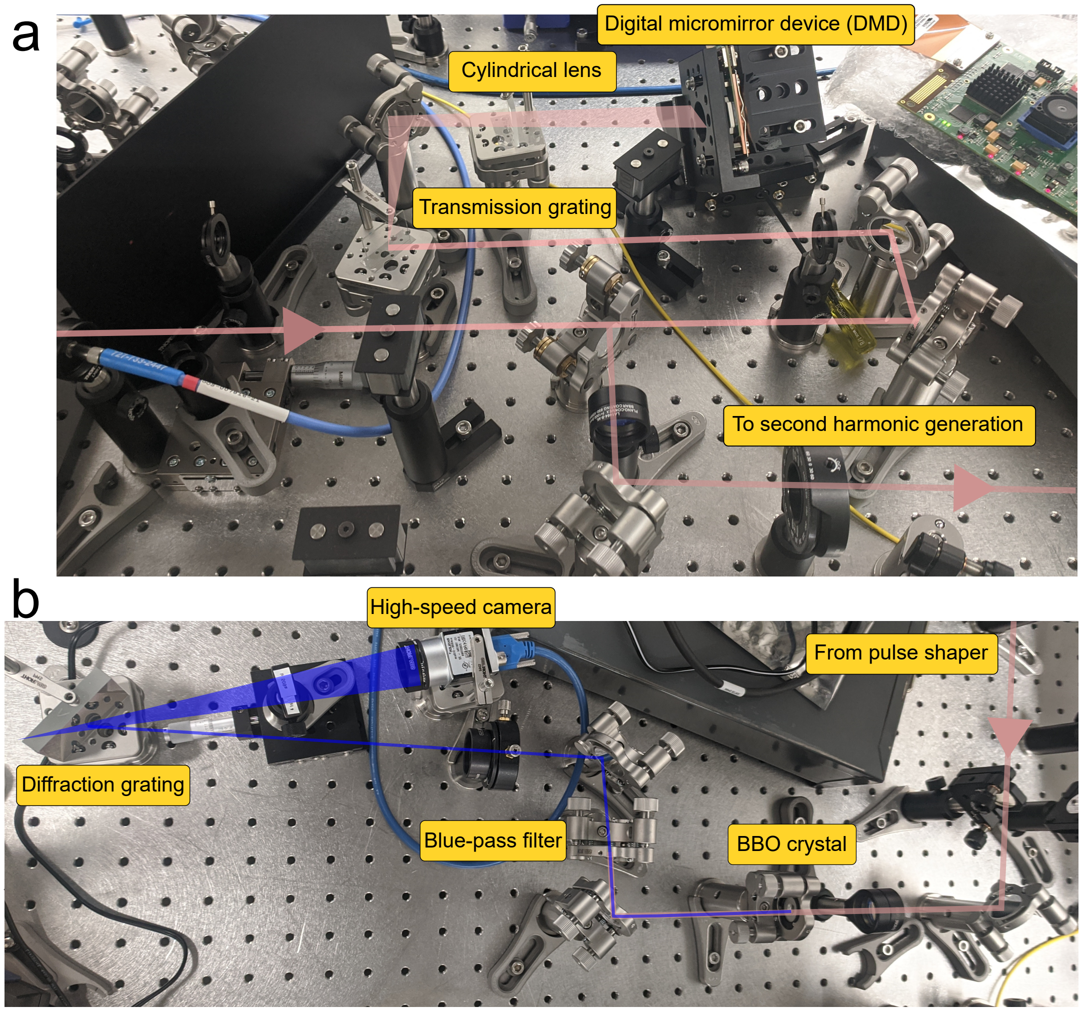

Fig. S8: Labelled photos of the pulse shaper and second-harmonic generation experimental setups.

Our ultrafast second-harmonic generation (SHG) PNN is based on a mode-locked Titanium:sapphire oscillator (Spectra Physics Tsunami) which is operated on by a digital micromirror device (DMD, Vialux V-650L)-based pulse shaper to produce complex, amplitude-modulated pulses according to input data and parameters. The spectrally-

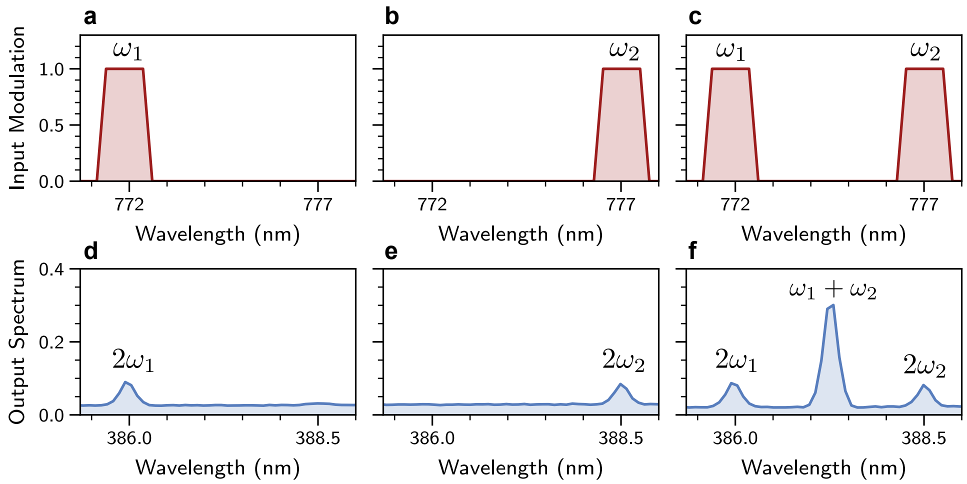

Fig. S9: Characterization of the femtosecond second harmonic generation (SHG) experimental setup with simple inputs. a-c. Sharply peaked input signals to the pulse shaper, which results in continuous-wave like input waves for SHG. d-f. The resulting output spectrum of femtosecond SHG for their respective input signals.

modulated pulses, centered around 780 nm, are then focused into an 0.5 mm long BBO crystal where SHG occurs. After filtering the residual 780 nm light, the resulting blue ($\sim$ 390 nm) light is diffracted by a ruled reflection grating and focused onto a fast camera (Basler ace acA800-510u) to measure the SHG spectrum. Annotated photos of the setup are shown in Supplementary Figure S8.

Training PNNs requires executing the physical evolution millions of times, so it was prudent to design the setup to allow for high-throughput operation. Based on the design described above, we were able to operate the SHG-PNN at a sustained rate of 200 Hz, reliably over months (including remotely during the COVID-19 pandemic).

The DMD-based pulse shaper achieves continuous spectral amplitude modulation in the following way. Light diffracted by a transmission grating (Ibsen Photonics PCG-1765-808-981) is focused by a cylindrical lens (150 mm, Thorlabs) onto the DMD, which is mounted at 45 degrees from the vertical so the micromirrors' rotation axis is vertical with respect to the table. The DMD is then rotated slightly so that the light reflected by the mirrors in the '0' state retroflects backward through the cylindrical lens and grating, following an identical path except with a slight downward angle so as to be separated by a pick-off mirror. By writing vertically-oriented (with respect to the table, 45 degrees to the DMD's axes) gratings of varying duty cycle to the DMD, we can achieve multi-level amplitude modulation at each spectral position (the frequency components are dispersed across the DMD in the direction parallel to the table by the grating). The design of this pulse shaper was inspired by Ref. [67], and to control the DMD using Python we adapted code developed for Ref. [68] available at Ref. [69].

While such a design allows continuous amplitude modulation at significantly higher speeds than most spatial light modulator-based pulse shapers, it results typically in pulses with complex spatiotemporal coupling which would be challenging to account for with traditional simulations. In this regard, our data-driven digital models were helpful in allowing the physical apparatus to be designed to optimize the reliability and speed of the experiment, rather than to ensure for the physics to easily be modelled by traditional simulations.

2. *Input-output transformation characterization*

Here, we characterize the input-output transformation of femtosecond SHG.

In Fig. S9, we characterize the SHG setup with simple quasi-cw inputs to elucidate the basic physics of the device.

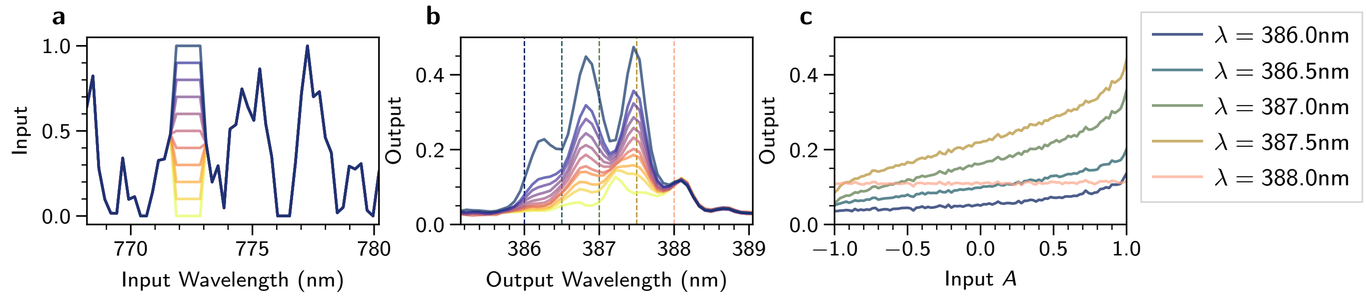

Fig. S10: Response of femtosecond SHG device to a complex-waveform input whose spectrum has a small flat region with an amplitude that is varied. **a.** Waveform for pulse shaper to apply to optical pulse, which are the physical inputs of the SHG system. Different colors correspond to different values (A) that the small flat region takes on. **b.** Output spectrum from SHG. Each curve of a particular color, is the output of the SHG system subject to input signals with the same color in subpanel a. **c.** Slices through output spectrum as a function of the input section amplitude A, which shows the nonlinearity of the transformation implemented by SHG.

In Fig. S9a and b, we consider continuous-wave-like inputs that are sharply peaked in wavelength. The corresponding SHG outputs in Fig. S9d and e are also sharply peaked in wavelength, with half the wavelength. In panel c, we consider an input that is the sum of input a and b; the output shown in panel f, illustrates the nonlinear nature of SHG, as it is not the sum of output d and e. There is an additional strong output at a center wavelength resulting from combining both input beams at 772nm and 777nm.

Having characterized its basic behavior, we illustrate the complex controllable transformation of the SHG device in Fig. S10, by showing its response to a complex input waveform. Figure S10a shows input waveforms in which a small section has been held constant to a fixed amplitude A, for varying values of A between 0 and 1. Figure S10b shows the resulting output spectrum from the SHG, which illustrates the complexity of the spectrum; The output can be significantly different across a wide portion of the spectrum despite the changed portion in the input being relatively small. Figure S10c plots the output spectrum at a particular wavelength for varying values of the amplitude A, which also shows that the nonlinearity is strongest for large amplitude ($A \approx 1$).

B. Analog transistor PNN

1. *Experimental setup*

The electronic PNN experiments were carried out with standard bulk electronic components, a hobbyist circuit breadboard, and a high-speed DAQ device (Measurement Computing USB-1208-HS-4AO), which allowed for a single 1 MS/s analog input and output. A schematic of the setup is shown in Supplementary Figure S11.

A variety of circuits were explored over the course of this work. The one used ultimately for the MNIST digit classification task was designed to be as simple as possible while still exhibiting very strongly nonlinear dynamics. The inclusion of a RLC oscillator within the circuit was inspired by the relative success of the oscillating plate (described in the next section), which we found was easily able to achieve good performance on the mostly-linear MNIST task.

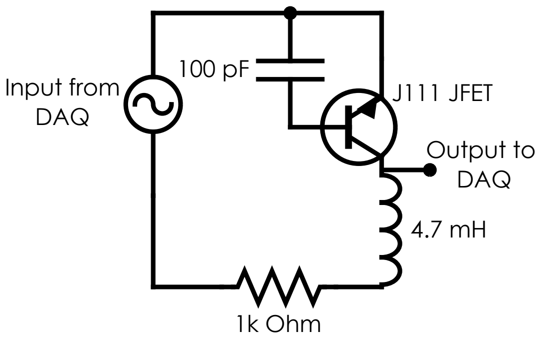

Fig. S11: Schematic of the analog electronic circuit used for the electronic PNN.

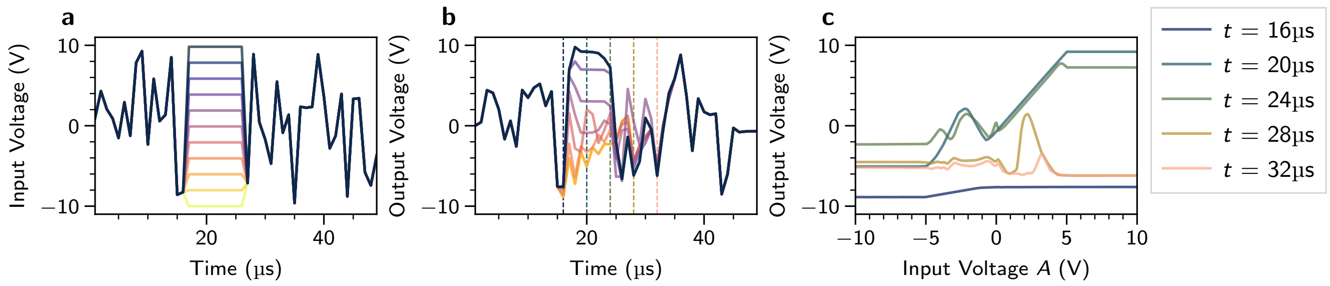

Fig. S12: Response of nonlinear analog transistor circuit to a complex waveform with one varied constant section. **a.** Input waveforms (different colors correspond to different input waveforms, where a section in the middle has been held constant to different fixed voltages). **b.** Output waveforms (different colors correspond to the different input traces in a). **c.** Slices through output waveforms as a function of the input section amplitude show the strong nonlinearity of the circuit's transformation of the input.

2. *Input-output transformation characterization*

Here we briefly discuss characterization of the circuit's response. Supplementary Figure S13 shows the impulse response of the circuit to an impulse with a varying amplitude. Supplementary Figure S12a shows a complex input waveform in which a section has been held constant to a fixed amplitude A, for varying values of A from -10 to 10 V. Supplementary Figure S12b shows the resulting output time series, illustrating both the nonlinearity of the response as well as the complex coupling between outputs after the changed value. Finally, Supplementary Figure S12c illustrates the nonlinearity of the circuit's response more clearly by plotting the output voltage of the indicated output time bins as a function of the input amplitude voltage A. The individual curves are highly nonlinear with respect to the input voltages.

At the highest input amplitudes in Fig. S12c, an additional nonlinearity due to the limiting of the DAQ can be observed. While this nonlinearity is not strictly a part of the electronic circuit, we opted to allow the PNN to operate in this regime to illustrate how PNNs can inherently utilize such "undesired" nonlinearities, and because the circuit's response was already highly nonlinear, so we did not expect this additional nonlinearity to significantly change the circuit's computational capabilities (in contrast, it was important to avoid this DAQ-based nonlinearity in our oscillating plate experiments described next, since the oscillating plate's input-output response was determined to be very linear).

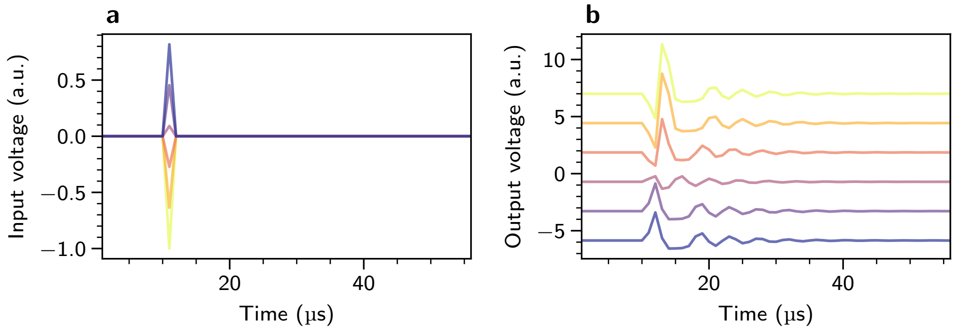

Fig. S13: Impulse response of the nonlinear analog transistor circuit to impulses of varying amplitude. The circuit's response is that of a nonlinear oscillator. In b, the different curves are offset artificially to improve visual clarity.

C. Oscillating plate PNN

1. *Experimental setup*

The setup of the oscillating plate PNN consists of an audio amplifier (Kinter K2020A+), a commercially available full-range speaker, a microphone (Audio-Technica ATR2100x-USB Cardioid Dynamic Microphone), and a computer controlling the setup.

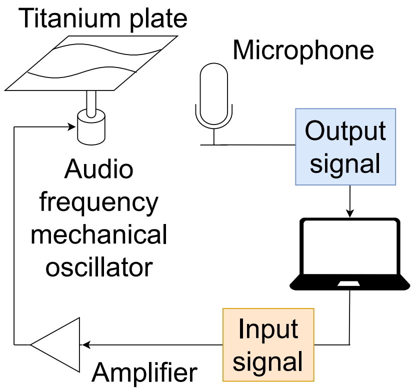

Fig. S14: A schematic of the information flow in the oscillating plate experimental setup. An input and control signal from the computer is amplified and applied to the mechanical oscillator (realized by the voice coil of an acoustic speaker). A microphone records the sound produced by the oscillating plate and returns it to the computer.

We use the speaker to drive mechanical oscillations of a 3.2 cm $\times$ 3.2 cm $\times$ 1 mm titanium plate that is mounted on the speaker's voicecoil. The diaphragm has been removed from the speaker such that most of the sound produced stems from the oscillations of the titanium plate. In Supplementary Figure S15, the steps taken to construct the oscillator with the mounted plate are shown.

2. *Input and output encoding*

Similar to the electronical PNN, we encode the physical inputs in the time domain. Supplementary Figure S16 shows the different steps of encoding and decoding signals in the oscillating plate PNN. First, inputs are encoded in a time-series of rectangular pulses that are transformed into an analog signal at 192 kS/s by the laptop's soundcard DAC. The signal is amplified by an audio amplifier and drives the voicecoil of a speaker that in turn drives the

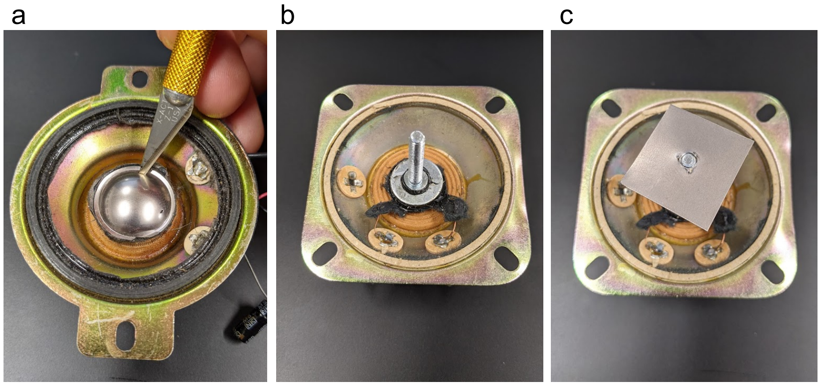

Fig. S15: Photographs of steps taken to construct an audio-frequency mechanical oscillator from a commercially available speaker. **a.** First, we remove the diaphragm and dust cap from a speaker with a precision knife to expose the voice coil. **b.** Next, we glue a screw and washer to the voice coil with commercially available two component glue. **c.** After letting the glue dry for 24 hours, we attach the titanium plate on the screw. Finally, we securely mount the speaker on a stable surface to suppress vibrations of the whole device. Different speakers were used over the course of the experiment and not all speakers shown above are the same model.

oscillating titanium plate. We ensured that both the soundcard DAC and the pre-amplifier are operated in a regime where their input-output relation is completely linear. The signal arriving at the oscillating plate was therefore a linearly amplified and slightly low-pass filtered version of the input and control signal created by the computer. The oscillations of the plate produce soundwaves which are recorded by a microphone and converted into digital signals at 192 kS/s. The signal is further downsampled by partitioning it into a number of equally long subdivisions and averaging the signal's amplitudes over this window. The length of the window is determined by the desired output dimension of the PNN layer.

Inputs and outputs are synchronized by a repeating trigger signal which precedes every sample that is played on the speaker. By overlapping the trigger signals we can synchronize samples to about 5 µs (1/sampling frequency).

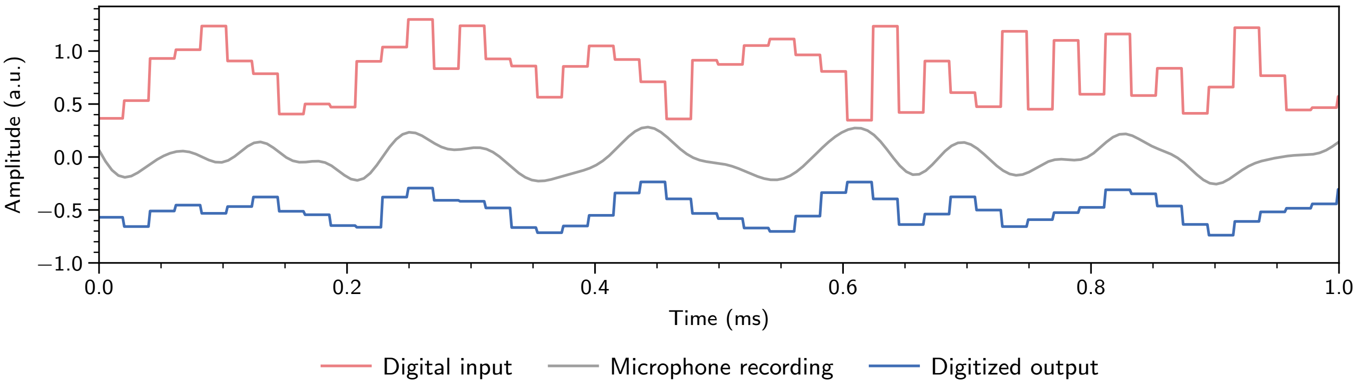

Fig. S16: An example of a 48-dimensional digital input to the oscillating plate setup (red). After driving the oscillating plate, the signal is recorded by the microphone (grey) and digitized in time to the desired output dimension, here 24 (blue). Y-axis units are arbitrary normalized amplitudes, and curves are offset vertically for ease of viewing.

3. *Characterization of input-output transformation*

While we initially aimed to create high-amplitude, nonlinear oscillations on the titanium plate, we realized that we could not reach such amplitudes with our setup. In Fig. S17c, we observe that in the time-domain the output voltage's response is overwhelmingly linear with respect to the input voltage.

Neglecting noise and assuming the oscillations of the plate are completely linear, each input to the plate can be regarded as exciting a band of modes between the frequency of the input and the Nyquist frequency of the digital sampler (192 kHz). Following their excitation, the modes decay in a transient oscillation depending on the damping of the material, thereby affecting the following outputs in a way that can be expressed as a convolution: $y_k = \sum_{0<j<k}^{N} c_{k-j} x_{j+1}$. Here y_k is the kth output, x_j is the jth input, and the c_j coefficients are constants determined by the medium of the oscillation and the excited modes.

As a matrix operation, the map from inputs to outputs can be approximated by:

$$\begin{pmatrix} y_1 \\ y_2 \\ y_3 \\ \vdots \\ y_d \end{pmatrix} = \begin{pmatrix} c_1 & 0 & 0 & \dots & 0 \\ c_2 & c_1 & 0 & \dots & 0 \\ c_3 & c_2 & c_1 & \dots & 0 \\ \vdots & \vdots & \vdots & \ddots & \vdots \\ 0 & 0 & 0 & \dots & c_1 \end{pmatrix} \begin{pmatrix} x_1 \\ x_2 \\ x_3 \\ \vdots \\ x_d \end{pmatrix} \tag{23}$$

For a typical input signal to the oscillating plate PNN, the resulting output is shown in Fig. S17a and b. In Fig. S17a, eleven input time-series that are identical except for 20 inputs at around 0.3ms are plotted. Inputs change with a frequency of 192 kHz. The transient oscillation set in motion by the inputs at 0.3ms persists for about 2ms (not completely shown in the figure). Hence, at this input frequency, an output of the oscillating-plate PNN is a convolution of approximately $N = 384$ previous inputs. If we encoded inputs at a slower frequency, for example 5 kHz, we would only convolve the previous $N = 10$ inputs. Therefore, by controlling the frequency of inputs, the oscillating plate emulates the operation of a convolution with variable kernel size and fixed kernel coefficients. This feature was utilized by designing our PNN for MNIST handwritten digit classification.

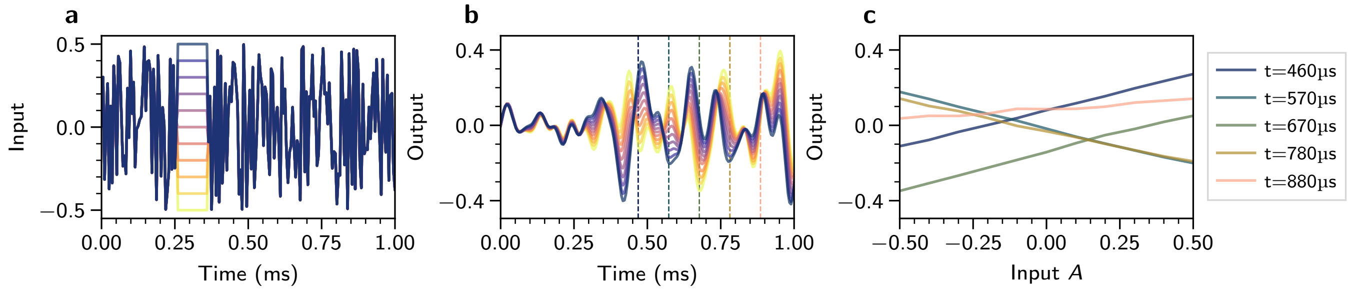

Fig. S17: Response of oscillating plate to a complex waveform with one varied constant section. **a.** Input waveforms. **b.** Output waveforms. Due to causality, all outputs before 0.3 ms are the same (up to noise). Afterwards, different input voltages produce different transient oscillations. **c.** Slices through output waveforms as a function of the input voltage (in arbitrary units) show the overwhelmingly linear response of the output to the input voltage.

D. Data-driven differentiable models

1. *Digital model for the mean*

As explained in Section 1, to approximate the gradients of physical systems, one needs a differentiable digital model to perform autodifferentiation on in the backward pass of PAT. In principle, any differentiable model could be used, such as a traditional differential equation physics model, some other analytic model, a data-driven model like a deep neural network, or a physics-informed neural network. In this work, we found that using neural architecture search [66] to find optimal deep neural network architectures allowed for the most accurate digital models across the physical systems we considered, and thus this was our default approach.

Of course, we expect that including physical insight into the differentiable digital model will usually result in better performance for any specific system, or at least allow for faster training of the digital model. Since any system used as a PNN will be ideally capable of being executed many times very quickly, we expect that it will usually be beneficial to include significant data-driven components in the digital model, since obtaining a large volume of training data will be straightforward. In some cases conceivable in the future, however, it may be more challenging to rapidly change the physical system's parameters, and the input-output dimension of the physical system used may be extremely high, such that even a large training data set significantly undersamples the space. In these and other cases, it is obvious that using physical insight to permit accurate differentiable digital models with relatively little training data will become essential.

The accuracy of the DNN models we obtained for the SHG and nonlinear analog circuit systems will be shown and commented on in later sections. Here, we will give a summary overview of our methods and the models ultimately used.

We first collected training data by recording the outputs of the physical system for a selection of N inputs/parameter vectors, where N was usually $\sim 10^4$ and the input/parameter vectors were usually drawn from a uniform distribution. We also investigated more sophisticated input distributions, but found these were generally unnecessary to produce good results (at least for the results considered in this work). This data set, consisting of N input/parameter vectors and their corresponding output vectors, was split into training and validation sets and used to train neural networks whose hyperparameters were found through a neural architecture search.

To perform our neural architecture searches, we used the Optuna package [66] with the following typical search space:

1. Number of layers between 1 and 5
2. Each layer width between 20 and 1500 units
3. Initial learning rates between 10^{-5} and 10^{-2}

The swish activation function was found to perform best, and we used the Adam optimizer.

For the SHG system, the optimal architecture was found to be a deep neural network with the following layer dimensions: [100, 500, 1000, 300, 50].

For the electronic circuit, the optimal architecture was found to be a deep neural network with the following layer dimensions: [70, 217, 427, 167, 197, 70].

For the oscillating plate, we initially tried to find a deep neural network architecture. However, once we realized the system dynamics were linear, we instead adopted a fully linear model, which was implemented in our deep neural network training framework by creating a network with no hidden layers.

2. Digital model for the noise

The digital models we used also include a model of the physical system's noise (including its dependence on the particular input/parameter vector to the system). We used these noise models for two key reasons. First, we were often interested in simulating PNN architectures and wanted to have a reasonably accurate prediction of their performance in the experiment. This was especially useful for slow physical systems, like the oscillating plate, for which MNIST experiments were much too slow to allow us to perform systematic examination of different architectures. More importantly, we wanted to ensure that we maximized the performance of *in silico* training, and providing an accurate model of the system's noise is a logical, standard technique to help mitigate the simulation-reality gap. *All the results for training with simulation presented in this work (including in Figure 3) were obtained with a simulation model of the physical system noise.*

We find however that while including a sophisticated model of physical system's noise in a simulation does improve the ability of *in silico* training to estimate the approximate performance level of results obtained in the lab, including noise in the simulation does not allow *in silico* training to perform as well as PAT for training the actual physical system.

A total digital model incorporating both mean-field and noise predictions needs to learn the following stochastic map: $y = f_{\text{mean}}(x) + n_{\text{noise}}(x)$, where $x \in \mathrm{R}^{N_{\text{in}}}$ is the input to the PNN, and y is a non-deterministic output from the PNN. This output has a deterministic component (given by the mean-field digital model f_{mean}), and a "noise" component $n_{\text{noise}}(x) \in \mathrm{R}^{N_{\text{out}}}$ whose distribution must agree with the true physical system's input-dependent noise.

Note that the digital model needs to learn the noise distribution from *any* given input/parameter vector x (for brevity, we will refer to x as just the input vector from here onward, but note that it includes both the parameters and input data to the physical system). For simplicity, we first outline how to learn the noise distribution for a given fixed input vector, denoted as $x^{(\text{fixed})}$ before addressing the more general case of predicting the noise for any input vector.

To learn the noise distribution for this fixed input, we first collect samples of outputs for the same fixed input. We execute the physical system N_{repeat} times with the same input vector $x^{(\text{fixed})}$. The output vectors will be denoted $y^{(fixed,k)}$ where $k = 1, ..., N_{\text{repeat}}$. From experimental measurements, we have ascertained that the output distribution of the physical systems considered in this work are reasonably well described by a multivariate normal distribution. Thus, their entire noise distribution is well characterized by the mean vector $\mu_i^{(fixed)} = \sum_k y_i^{(fixed,k)}$ predicted by the mean-field digital model, and a covariance matrix $\Sigma_{ij}^{(fixed)} = \frac{1}{N_{\text{repeat}}-1} \sum_k (y_i^{(fixed,k)} - \mu_i^{(fixed)})(y_j^{(fixed,k)} - \mu_j^{(fixed)})$.

How can we generate typical additive noise on top of the mean-field output vector, given the covariance matrix? The answer is simple: we need to find a matrix $A^{(fixed)}$ that satisfies $A^{(fixed)} A^{(fixed)^{\mathrm{T}}} = \Sigma^{(fixed)}$. Once we have the A matrix, a sample of the noise $n \in \mathrm{R}^{N_{\text{out}}}$, which has the correct distribution, can be obtained from a matrix multiplication between $A^{(fixed)}$ and a vector of i.i.d normal noise $z_i \sim N(0, \sigma^2 = 1)$, i.e. $n = A^{(fixed)} z$.

So far, the dimensions of A has been left unspecified. In principle, if the noise is sufficiently simple (e.g., the covariance matrix is rank 1), the covariance matrix can be decomposed as an outer product between a single vector and itself, i.e. $A^{(fixed)} \in \mathrm{R}^{N_{\text{out}} \times 1}$. We can generalize this concept of compressing the representation of the noise via the following procedure. First perform the spectral decomposition on $\Sigma^{(fixed)}$ to arrive at UDU^{T}. Then, by looking at the eigenvalue spectra, ascertain how many dominant eigenvalues are present in the covariance matrix and denote that number as N_λ. Then let $A^{(fixed)} = U\sqrt{D}[:, 1 : N_\lambda]$, to arrive at a compressed $A^{(fixed)}$ matrix with dimension $N_{\text{out}} \times N_\lambda$. For training our noise digital models, we find it is helpful to limit the number of eigenvectors of the covariance matrix, so that the dimension of the input-output map that must be learned is as small as possible (and because higher-order eigenvectors are not usually important).

To summarize, for a given input vector $x^{(fixed)}$, we only need to learn $A^{(fixed)} \in \mathrm{R}^{N_{\text{out}} \times N_\lambda}$ to output samples of the noise. Thus, to learn a noise digital model for general inputs, we simply train neural network to, given many

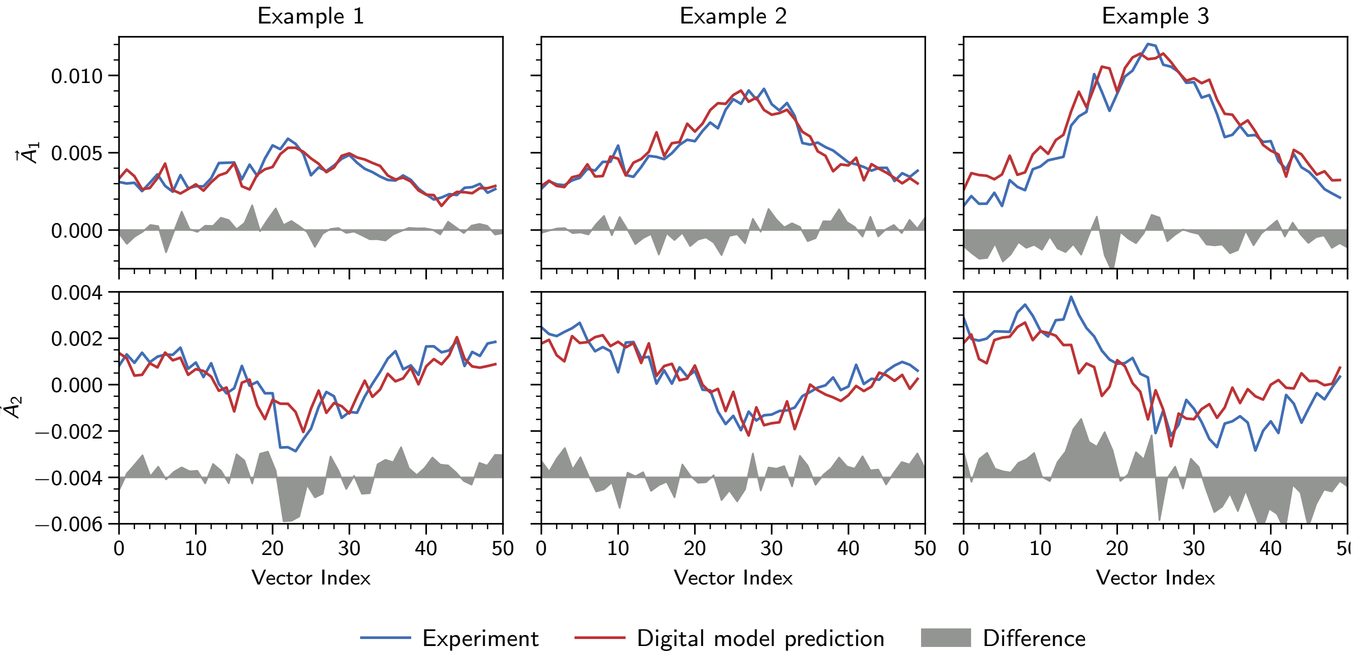

Fig. S18: Characterization of the noise digital model. Here, the digital model (red) predicts the 2 dominant noise eigenvectors $\vec{A}_1$ and $\vec{A}_2$ for three different 50-dimensional physical inputs to the SHG device. The experimental noise eigenvectors (blue) are derived from diagonalizing the sampled covariance matrix from 30 repeated measurements. Each row represents a distinct physical input.

different inputs $x^{(q)}$, predict the corresponding $A^{(q)}$ for any given $x^{(q)}$. This corresponds to a neural network that outputs a vector corresponding to a flattened A (a vector of dimension $N_{\text{out}}N_{\lambda}$). Training data for this network is obtained by measuring the output of the physical system many times (we found 40 repeats was usually sufficient) for a nominally-fixed $x^{(q)}$ in order to compute the corresponding $A^{(q)}$ (repeating the above procedure for each training example). When used in the forward pass of the digital model for training in simulation, the noise model NN's predicted vector A is reshaped to the matrix form and then used to generate a noise sample that is added to the mean-field digital model's prediction.

To provide examples of typical noise digital models, Supplementary Figures S18 and S19 compare, for the validation set, the performance of the noise digital model's prediction of the covariance eigenvectors and the covariance matrix to the experimental measurements of these quantities. The agreement is somewhat more approximate than what we obtain for the mean-field digital models (shown in the next subsections), but is overall excellent.

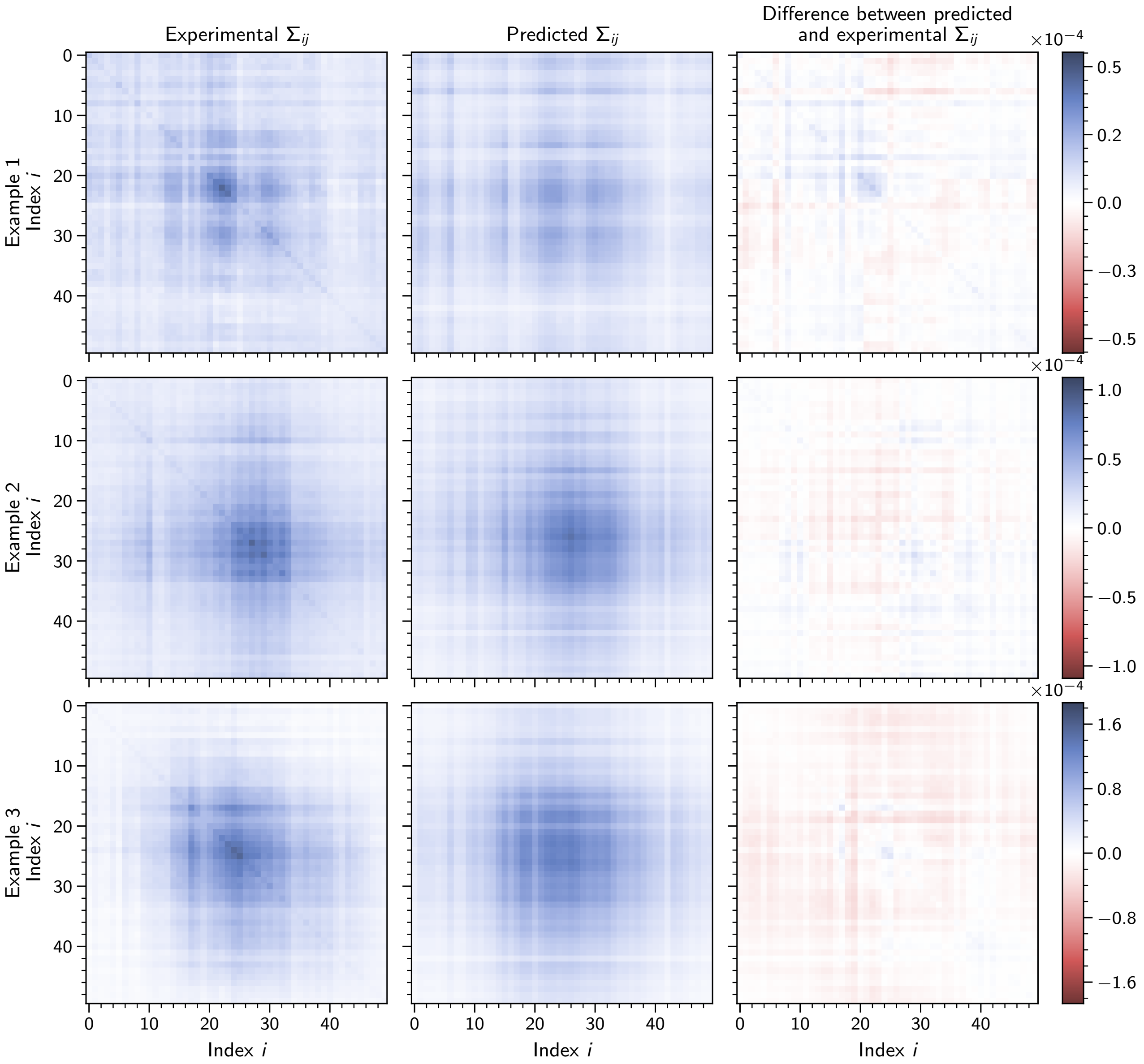

Fig. S19: Characterization of the noise digital model. Left column: The experimental sampled covariance matrix from 30 repeated measurements. Middle column: The predicted covariance matrix computed with the predicted dominant noise eigenvectors $\vec{A}_1$ and $\vec{A}_2$ that are shown in Fig. S18. Right column: The difference between the predicted and experimental covariance matrix. Each row represents a distinct physical input, and the covariance matrix for a given row shares a color bar that is shown on the right. The relatively low amplitudes visible in the right column show the strong agreement between the digital model's predicted noise distribution and the experimentally-measured distribution.

E. Descriptions of PNN architectures

In this section, we describe the PNN architectures used for the different machine learning tasks implemented with each physical system. In each subsection, we also show the performance of the differentiable digital model used, and (for the PNNs not thoroughly examined in the main paper) present results illustrating how the physical transformations perform the classification task.

Before mentioning specifics for each architecture, we mention several commonalities among all PNNs we present in this work.

First, the same basic training procedure was used in all cases. We found that the Adadelta optimizer [75] worked best, and speculate that its exclusion of momentum and parameter-dependent adaptation makes it well-suited to PNNs, whose parameters are generally not symmetric. We also usually utilized learning rate scheduling, but this was of secondary importance.

Second, in all cases we compared trained PNNs to baseline reference models in which all physical input-output transformations were replaced by identity operations. This allowed us to confirm that, despite each PNN architecture including some digital operations, the digital operations alone were not responsible for the majority of the functionality of the trained PNN model.

Third, for convenience, we sometimes performed some digital pooling operations to reduce the dimension of the measured physical output vectors. This is because the physical input-output transformations we considered (for convenience) had all equal input and output dimensions, and this operation allowed us to modify the dimensionality of the input-output transformations, allowing us to tackle machine learning problems with different dimensionality in the input (feature) vector and output vector (for instance, the MNIST dataset has a 196 input dimension and 10 dimensional output). Since this operation is not trained and it can only contribute a minor computational power to the PNN. Besides, any minor benefits it confers can easily be performed physically as it is equivalent to changing the sampling rate of output measurements (e.g., by using a slower digital-analog converter). Finally, we note that for the oscillating plate and electronic PNNs, downsampling operations of this kind were ultimately irrelevant, as both devices were operated at input and output sampling rates limited by the sound card and data acquisition device respectively.

Finally, in all cases it was necessary to include additional Lagrangian terms in the loss in order to ensure that physical inputs and parameters sent to physical systems were within the allowed ranges. These additional terms are identical to the Lagrangian terms that have been discussed earlier in Section 1 D.

1. *Figure 2-3: Femtosecond SHG-vowels PNN*

The femtosecond SHG-vowel PNN was chosen to be the first example PNN of the main manuscript for two reasons. First, the vowel classification task is simple enough to facilitate a relatively simple PNN, and second, the SHG physical transformation is relatively unusual, being a fully-nonlinear transformation with nearly all-to-all coupling of inputs. Although the second feature does not make SHG well-suited to the vowel-classification task (which is linear), we felt it served as a clear, small-scale tutorial demonstration of a multilayer PNN on a relatively high-dimensional task in which the physical transformation used is highly nonlinear. Vowel classification is a simple task, but has been regularly used in testing novel machine learning hardware [10, 11, 76].

To make the SHG-vowel PNN intuitive and illustrative for the concept of PNNs in general, we made several design choices. We used a portion of the pulse shaper's spectral modulations for trainable parameters, and a second, distinct portion for encoding input data. In total, each SHG input-output transformation maps 100 physical inputs, which are split between the input data and trainable parameters, to a 50-dimensional output SHG spectrum.

In Fig. S20, we show some representative input and outputs of this transformation, where physical inputs are split equally between the input and parameters (50 dimensional input and parameters each). Consistent with the analysis performed in Sec. 2 A 2, the transformation is complex, as evidenced by the widely-varying outputs. Nevertheless, this transformation can be effectively modeled via a data-driven model. By performing the neural architecture prescribed in Sec. 2 D, we found a deep neural network that is able to learn the transformation effectively. This digital model's excellent agreement with the experimental input-output transformation is shown in Fig. S18.

Having characterized the performance of the differentiable digital model, we now show how the SHG system performs vowel classification. To begin, we first describe the vowel classification task that we perform. We use the vowel dataset from Ref. [10], which can be downloaded at this `https://static-content.springer.com/esm/art%3A10.1038%2Fs41586-018-0632-y/MediaObjects/41586_2018_632_MOESM2_ESM.docx`, with an additional (untrainable) renormalization to account for the fact that the SHG system takes in physical inputs between 0 and 1. For completeness, we will paraphrase the description of the dataset that is in the supplementary information of Ref. [10], before describing the renormalization we apply to the dataset.

The vowel data considered is a subset of the Hillenbrand vowel database, which can be found at `https://homepages.wmich.edu/~hillenbr/voweldata.html`. In this dataset, each spoken vowel is characterized by their formant frequencies, which refers to the dominant frequencies (in units of Hz) present in an audio signal. More specifically, each vowel is characterized by a 12-dimensional vector, which contains the first three formants sampled at the steady state (whole audio signal) and three temporal sections, centered at 20%, 50% and 80% of the total vowel duration respectively. The subset of vowels we consider are the vowels spoken by 37 female speakers which have complete formant information (some vowels in the full dataset have formants that could not be measured). Since each speaker utters 7 different vowels, the vowel dataset contains a total of 259 vowels ($N_{\text{data}} = 257$).

In order to accommodate the finite input range of the SHG experiment, we normalize them via the element-wise min-max feature scaling, where each dimension of the input vector is renormalized by their corresponding maximum and minimum values (over all 259 vowels). Mathematically, if the original dataset is given by $\{\,(\boldsymbol{x}^{(k)}, \boldsymbol{y}^{(k)})\,\}_{k=1}^{N_{\text{data}}}$, the examples of the transformed dataset $\{\,(\boldsymbol{x'}^{(k)}, \boldsymbol{y}^{(k)})\,\}_{k=1}^{N_{\text{data}}}$ is given by $x'^{(k)}_i = \left(x^{(k)}_i - x_{\min,i}\right) / \left(x_{\max,i} - x_{\min,i}\right)$, where the element-wise minimum and maximum values are given by $x_{\min,i} = \min\limits_k x^{(k)}_i$ and $x_{\max,i} = \max\limits_k x^{(k)}_i$.

The full architecture for performing the vowel classification with the SHG system is schematically shown in Fig. S21. In the following, we outline the steps involved in performing an inference with the PNN. To show how the intermediate quantities evolve in the PNN, we plot quantities that are labelled by letter labels in Fig. S21 in subpanels of Fig. S22.

1. The 12-D normalized vowel formant frequencies are overall rescaled, i.e. $x_i \to a x_i + b$, where a and b are trainable parameters.

2. The 12-D vector is then repeated 4 times. The 48-D output is the input to the first SHG device, i.e. $\boldsymbol{x}^{[1]} = [x_1, x_1, x_1, x_1, \ldots, x_{12}, x_{12}, x_{12}, x_{12}]^{\mathrm{T}}$ to arrive at a 48-D input for the first SHG system. This 48-D input is shown in a of Fig. S21 and Fig. S22.

3. For the first SHG system, this input is concatenated with a 52-D parameter and the total 100-D physical input $\boldsymbol{q}^{[1]} = [\boldsymbol{x}^{[1]}, \boldsymbol{\theta}^{[1]}]^{\mathrm{T}}$ is sent to the first SHG.

4. The SHG performs the physical computation $\boldsymbol{s}^{[1]} = f_{\text{SHG}}(\boldsymbol{q}^{[1]})$. The output of the first SHG $\boldsymbol{s}^{[1]}$ is shown in b of Fig. S21 and Fig. S22.

5. The 50-D output of the first SHG $\boldsymbol{s}^{[1]}$ is renormalized and an overall trainable rescaling is applied, i.e. $\boldsymbol{y}^{[1]} = a^{[1]}\boldsymbol{s}^{[1]}/\max(\boldsymbol{s}^{[1]}) + b^{[1]}$, where $a^{[1]}$ and $b^{[1]}$ are trainable scalars.

6. The 50-D output is then concatenated with a 50-D parameter and sent to the next SHG device.

7. Step 4 and 5 are repeated until the output of the fifth SHG device has been renormalized and rescaled. Mathematically, they are given by,

$$\boldsymbol{x}^{[l]} = y^{[l-1]} \tag{24}$$

$$\boldsymbol{q}^{[l]} = [\boldsymbol{x}^{[l]}, \boldsymbol{\theta}^{[l]}]^{\mathrm{T}} \tag{25}$$

$$\boldsymbol{s}^{[l]} = f_{\mathrm{SHG}}(\boldsymbol{q}^{[l]}) \tag{26}$$

$$\boldsymbol{y}^{[l]} = a^{[l]}\boldsymbol{s}^{[1]}/\max(\boldsymbol{s}^{[l]}) + b^{[l]} \tag{27}$$

for $l = 2, 3, \ldots 5$. The output of each SHG device is shown in c-e of Fig. S21 and Fig. S22.

8. From the 50-D output, a 21-D section is cropped as follows $\boldsymbol{y}^{[\mathrm{c}]} = [y^{[5]}_{12}, y^{[5]}_{13}, \ldots y^{[5]}_{32}]^{\mathrm{T}}$. The 21-D output is shown in f of Fig. S21 and Fig. S22.

9. Each 3-D section of this new 21-D output is summed to arrive at the final output of the PNN, i.e. $\boldsymbol{o} = [y^{[\mathrm{c}]}_1 + y^{[\mathrm{c}]}_2 + y^{[\mathrm{c}]}_3, y^{[\mathrm{c}]}_4 + y^{[\mathrm{c}]}_5 + y^{[\mathrm{c}]}_6, \ldots y^{[\mathrm{c}]}_{19} + y^{[\mathrm{c}]}_{20} + y^{[\mathrm{c}]}_{21}]^{\mathrm{T}}$. This final output is shown in g of Fig. S21 and Fig. S22.

Analogous to Sec. 1 D, the PNN is trained by minimizing the following loss function:

$$L = H(\boldsymbol{y}, \mathrm{Softmax}(\boldsymbol{o})) + \lambda_\theta \sum_l L_{\mathrm{bound}}(\boldsymbol{\theta}^{[l]}, v_{\min}, v_{\max}) + \lambda_{\mathrm{x}} \sum_l L_{\mathrm{bound}}(\boldsymbol{x}^{[l]}, v_{\min}, v_{\max}) \tag{28}$$

where H is the cross-entropy function and $L_{\mathrm{bound}}(\boldsymbol{z}, v_{\min}, v_{\max}) = \sum_l \frac{1}{N_{\boldsymbol{z}}} \sum_i^{N_{\boldsymbol{z}}} \max(0, z_i - v_{\max}) - \min(0, z_i - v_{\min})$. The last two terms are regularization terms to constraint the inputs and parameters of each SHG device, whose parameters are $\lambda_\theta = 0.5, \lambda_{\mathrm{x}} = 2, v_{\min} = 0, v_{\max} = 1$.

Instead of vanilla stochastic gradient descent (as in (19)), we perform PAT with the Adadelta optimizer. This is because the adaptive learning rate is helpful for dealing with the different nature of parameters that exist in the model. The initial learning rate is 1.0 and it was reduced by half every 700 epochs.

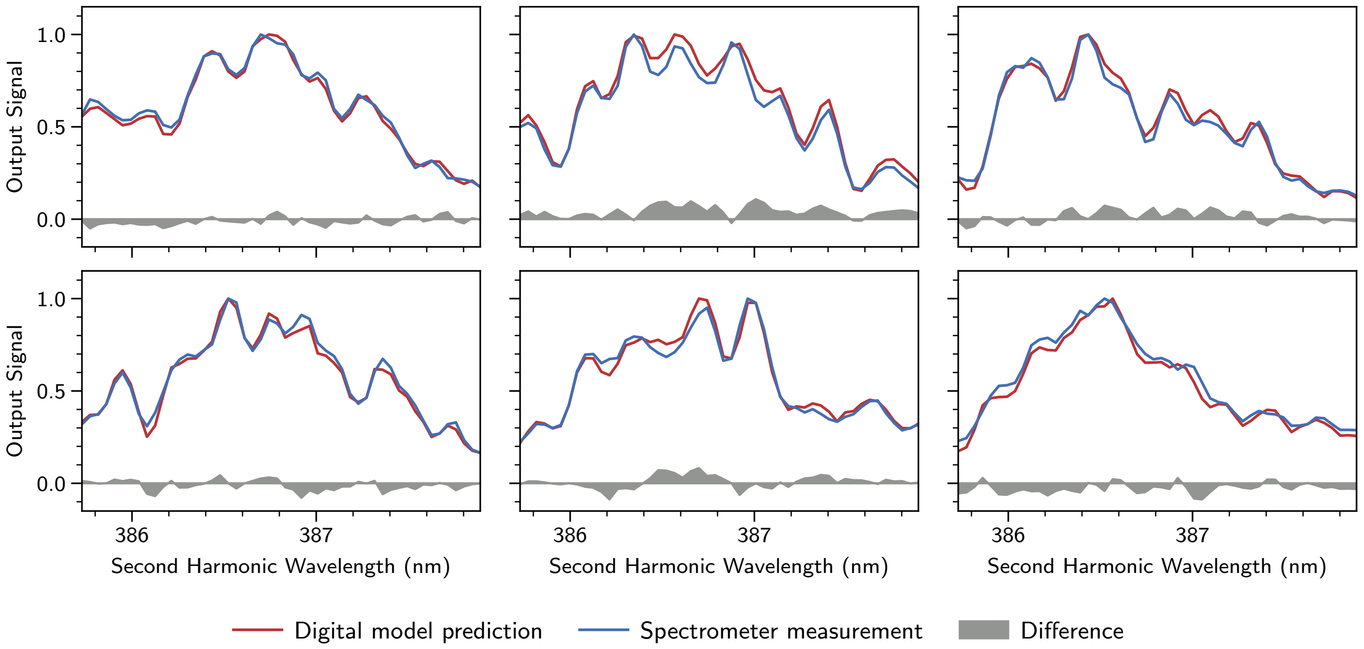

Fig. S20: Typical outputs and digital model predictions for the femtosecond SHG device. Here, 100 dimensional physical inputs are applied to the pulse shaper and the outputs of the SHG is 50 dimensional. As shown by the good agreement between the blue and red curves, the data-driven model emulates the physical system well. Note that the physical inputs considered here is part of the "test" set and has not been used to train the data-driven model.

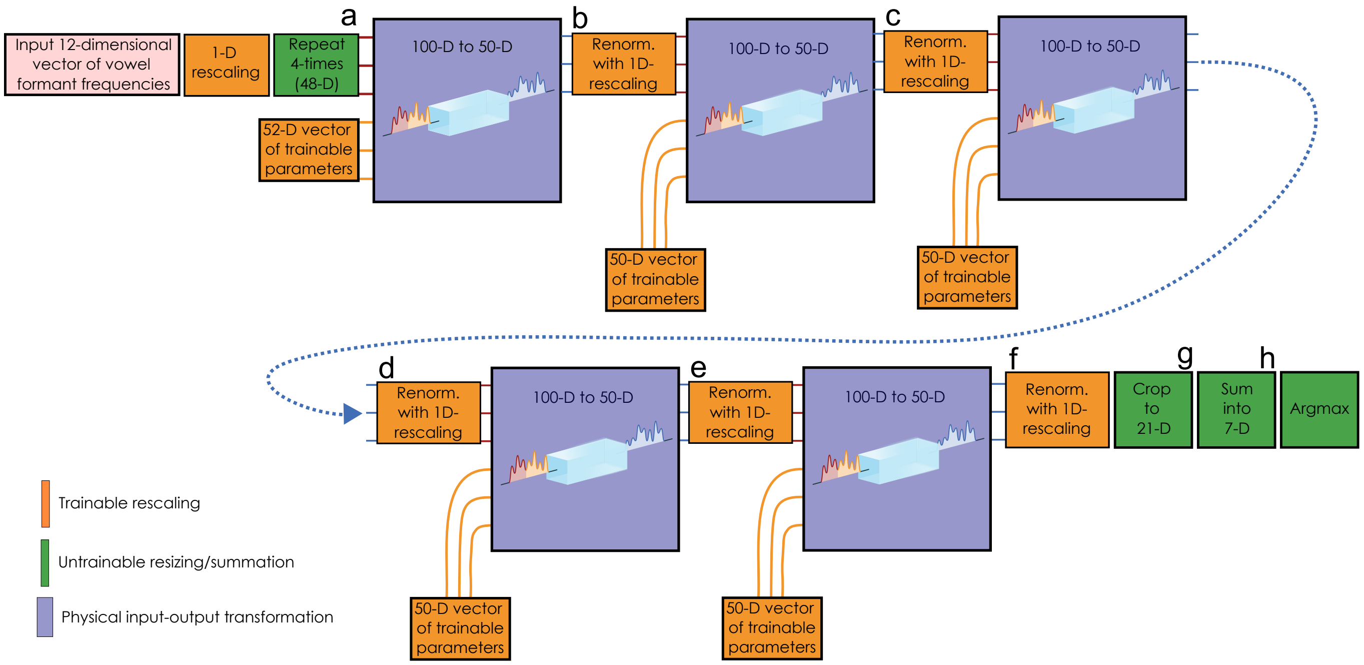

Fig. S21: The full PNN architecture used for the second harmonic generation vowel classifier (for the case of 5-layers). Annotation letters (a-h) refer to the plots in Fig S22. The architecture has a total of 12 trainable digital parameters, and 252 trainable physical parameters.

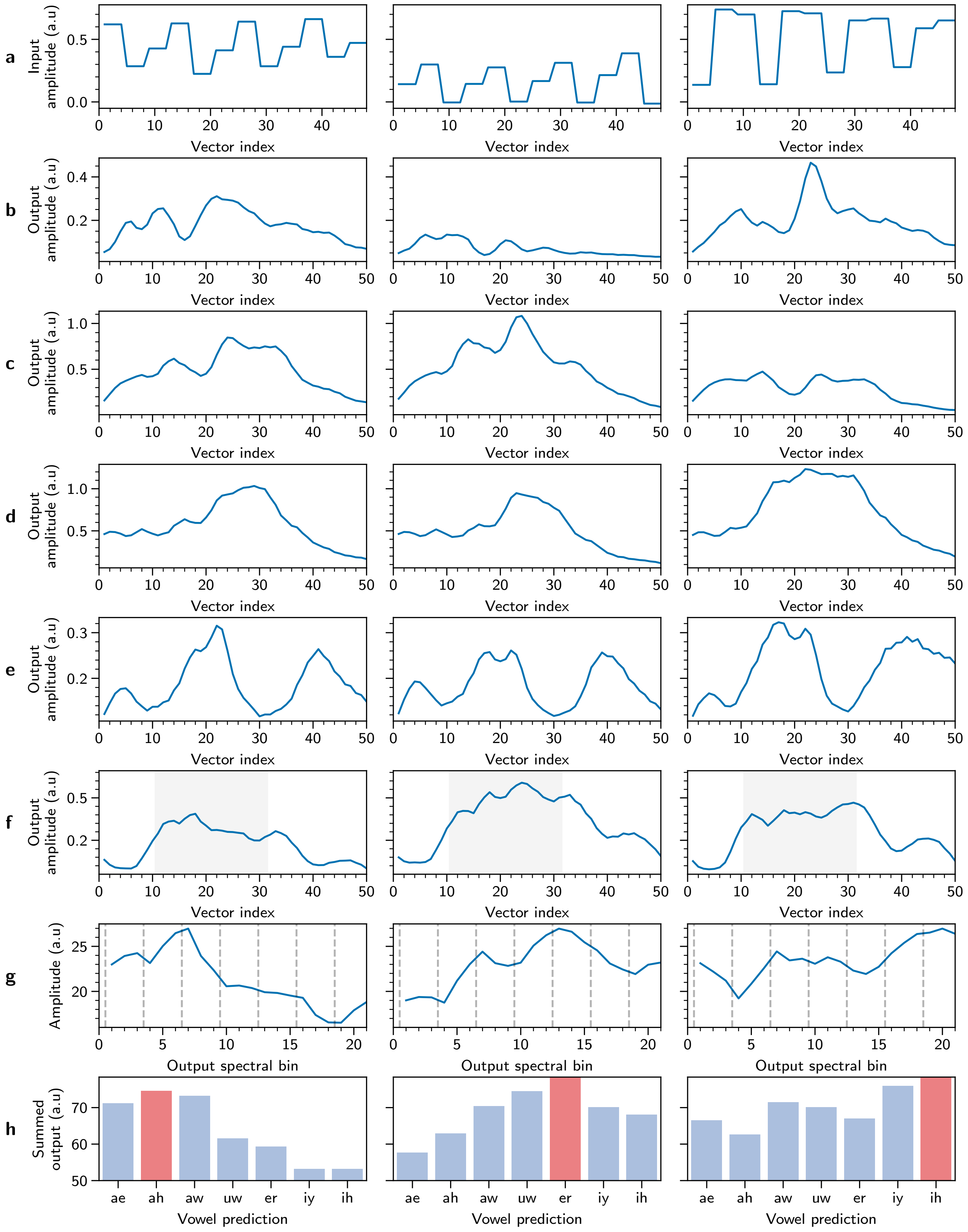

Fig. S22: The sequence of operations that make up the trained SHG PNN vowel model. The lettered labels corresponds to intermediate quantities that are labelled in Fig. S21. The red bars in part h indicate the correct vowel. Here, each column shows a different input vowel formant vector.

2. *Figure 4: Analog electronic MNIST handwritten digit image classification PNN*

As described in the main article, the electronic circuit chosen to serve as an electronic PNN demonstration was selected to exhibit highly nonlinear transient dynamics by embedding a transistor within an RLC oscillator.

This circuit was, unexpectedly, particularly challenging to produce an accurate differentiable digital model for. While we expect that incorporating knowledge-based components or other physical insight could improve the results, in order to keep our procedure general, we found that an effective workaround was to simply reduce the dimensionality of the input-output transformation. Empirically, we found that 70-dimensions (70 input samples, and 70 output samples) was small enough to achieve reasonable results.

The digital model was found by performing a neural architecture search. The best model found obtained a validation loss of 0.09 (with data normalized to -1 to 1). It is a 4-layer neural network with swish activation functions, and 217, 427, 167, and 197 hidden units. The Adam optimizer was used with a learning rate of 4.56×10^{-4}.

A related challenge with the analog electronic PNN was that our implementation exhibits high noise, mainly due to lost samples during streaming over the USB connection, and to jitter related to triggering at the limit of the device's temporal resolution. Even after implementing post-selection to account for traces corrupted or lost during the fast read-write operation of the DAQ, the mean deviation amplitude between the output voltage time series for two identical inputs was over 5% of the maximum absolute output amplitude, and nearly 20% of the absolute mean output amplitude.

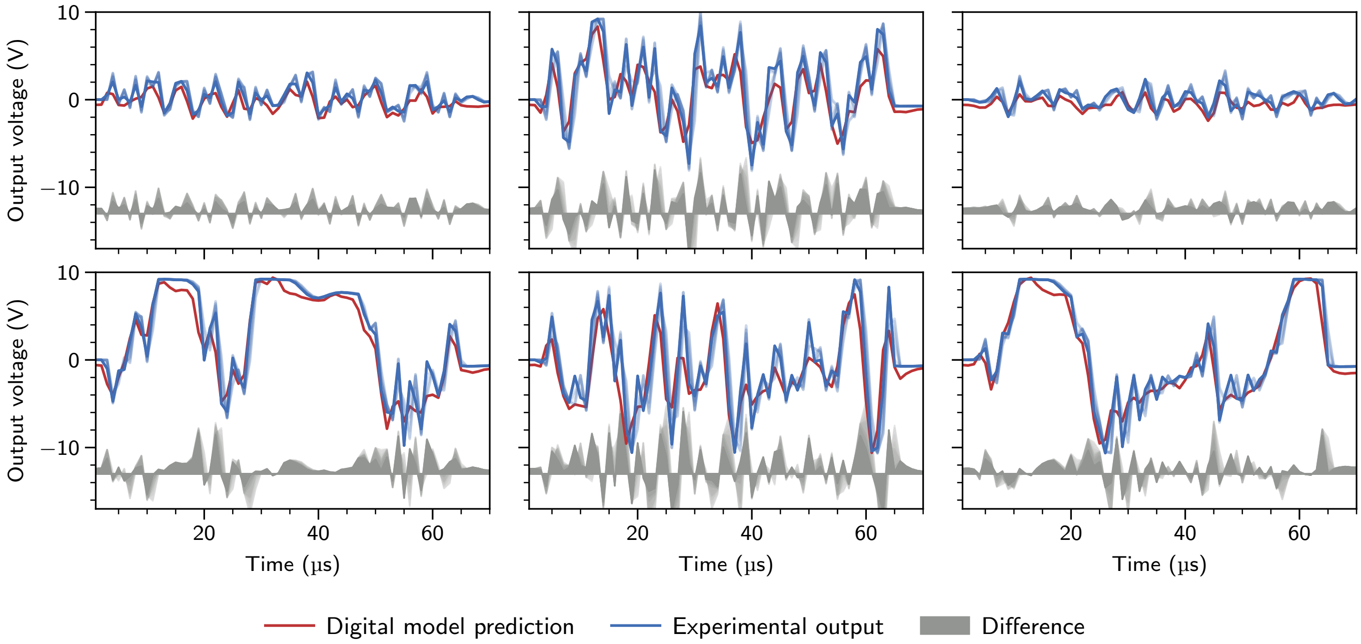

Fig. S23: Typical circuit outputs (blue) and digital model predictions (red) for the electronic circuit used for MNIST handwritten digit classification. For the circuit outputs, 40 traces are plotted, all for the same nominal input, in order to visualize the output noise behavior. The output of the circuit is complex and noisy, and the digital model is less accurate than for other systems.

Figure S23 shows the differentiable digital model's predictions compared to the circuit's actual output. As a reference point, the figure shows 40 output traces from the circuit, each taken for the same inputs. The figures illustrates that a main limiting factor in the digital model's accuracy is in fact the large, complex noise in the circuit's input-output transformation.

These two considerations, the low dimensionality of the input-output transformation, and the significant noise of the circuit's operation, influenced our PNN architecture design, shown in Supplementary Figure S24.

To make the 196-D MNIST images compatible with the 70-D input-output transformations of the electronic circuit, we chose to sum blocks of the MNIST image into 14-dimensional vectors (i.e., perform a pooling operation on the 196-dimensional vector in blocks of 14), then repeat this 14-dimensional vector 5 times as the input to the first layer of the PNN architecture. Of course, many other options are possible, but we found this strategy to be effective.

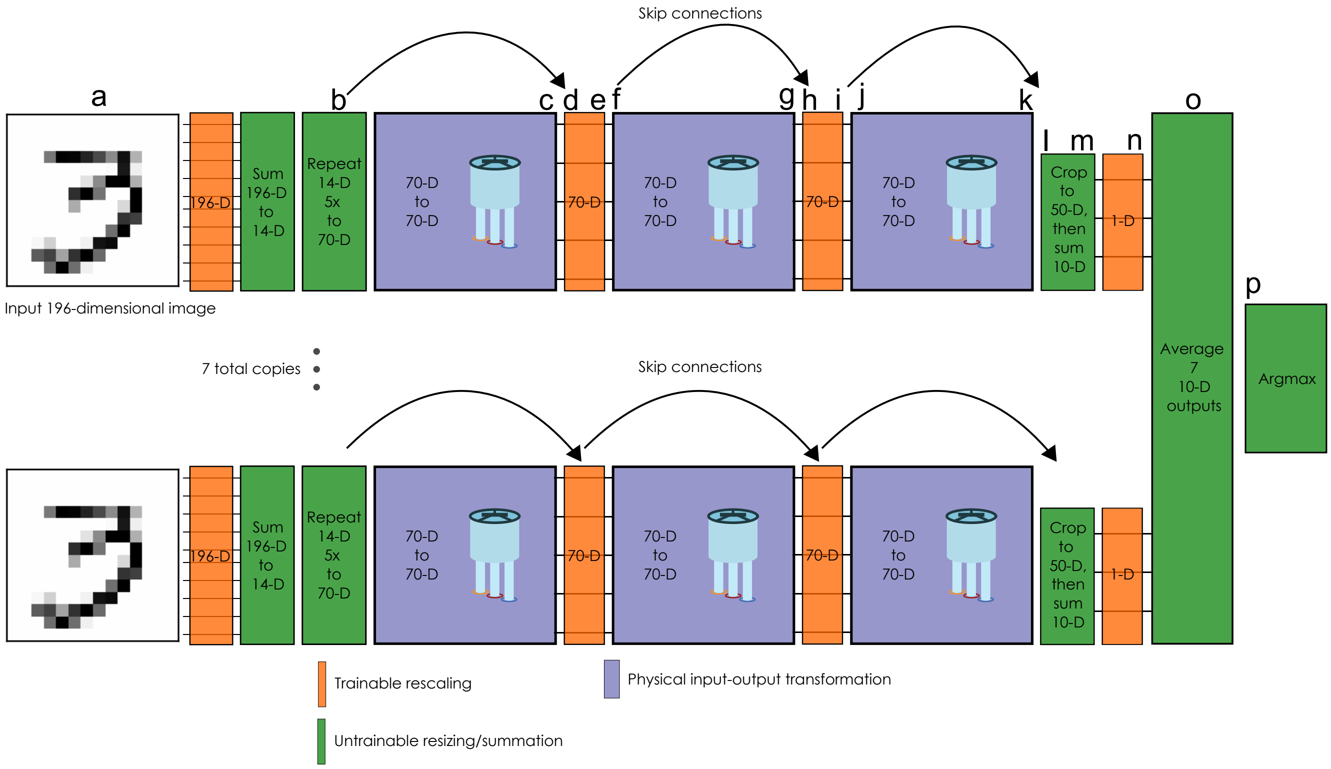

Fig. S24: The full PNN architecture used for the analog transistor circuit MNIST digit classifier. Annotation letters (a,b,c,d,...) refer to the plots in Fig S25 and the description in the main text. The architecture has a total of $7 \times 196 + 7 \times 2 \times 70 + 7 = 4711$ parameters, similar to the mechanical PNN.

To obtain robust performance despite the circuit's noise and the digital model's relatively poor accuracy, we chose to implement two additional design components. First, we operate the PNN as a 'committee': 7, 3-layer PNNs each produce a prediction, and the full PNN produces its prediction by taking a weighted average of these. Second, we added trainable skip connections between layers of the PNN. These connections, inspired by residual neural networks, were added to allow the network to act further as an ensemble of sub-networks [71], thus adding to the redundancy of the architectures (and, in principle, helping it to achieve good classification performance even when any one sub-part stochastically fails) [71].

Each inference of the full architecture involves the following steps. By letter labels (a,b,c, ...), we refer each step to its corresponding location in Supplementary Figure S24 and to the subpanels of Supplementary Figure S25.

1. 196-D MNIST images (a in Supplementary Figures S24 and S25, downsampled from 784-D) are element-wise rescaled, i.e. $x_i \to x_i a_i + b_i$, where a_i and b_i are trainable parameters .

2. Pool the 196-D vector from 196 to 14-D (i.e., sum each region of 14).

3. Concatenate this 14-D vector 5 times to produce a 70-D input for the first physical transformation (b in

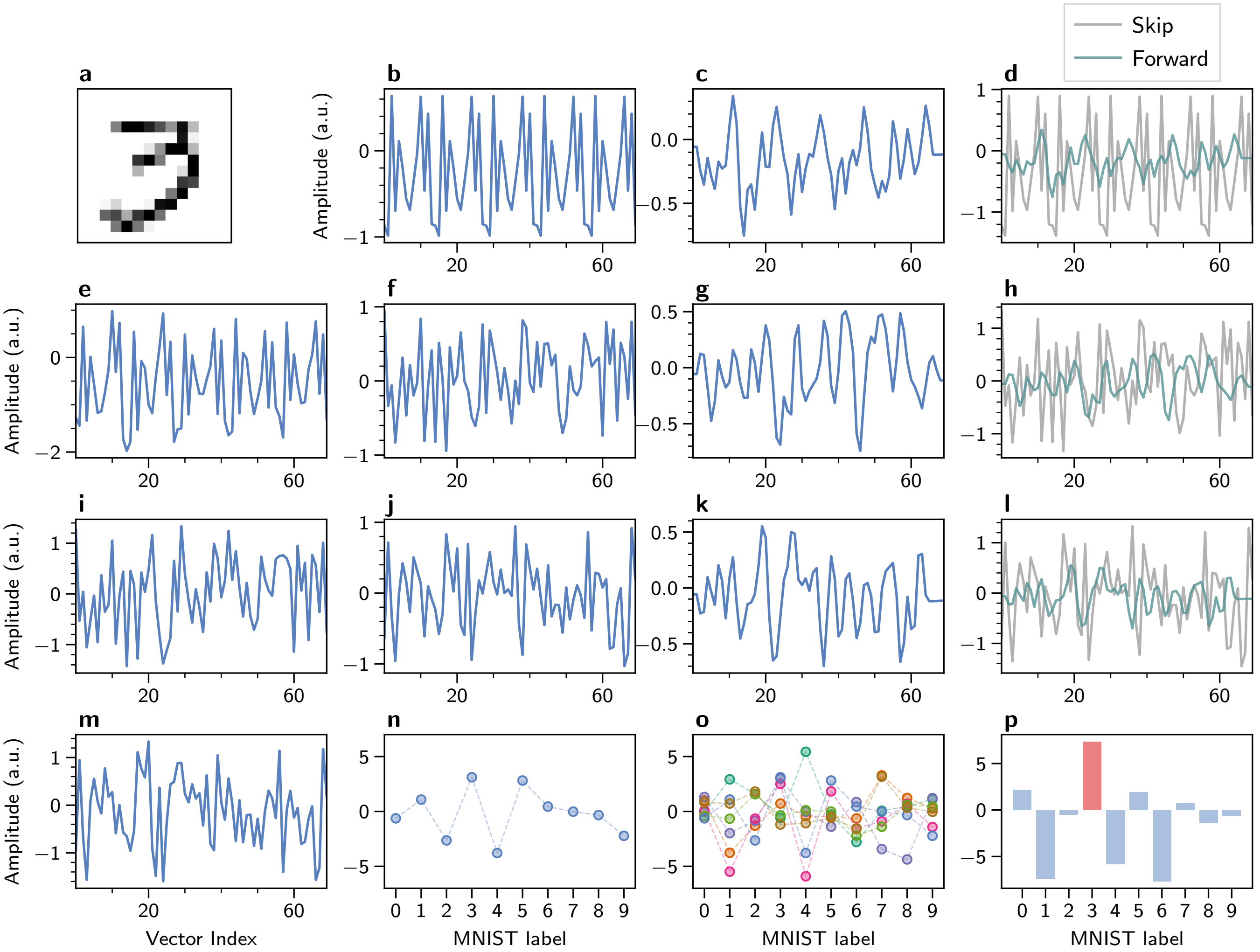

Fig. S25: The sequence of operations that make up the trained electronic PNN MNIST model. For details of each section, see main text. In part o, the different-colored curves correspond to the output from the 7 different 3-layer PNNs.

Supplementary Figures S24 and S25)

4. Run the physical transformation once, producing an output 70-D vector $\vec{y}$ (b-c in Supplementary Figures S24 and S25).

5. Add the output $\vec{y}$ to the initial input via trained skip connection, i.e. $\vec{y} \rightarrow \vec{y} + a\vec{x}$, where a is a trainable skip weight and $\vec{x}$ was the input to the physical system (d-e in Supplementary Figures S24 and S25).

6. Apply trainable, element-wise rescaling to produce the input for the second physical transformation, i.e., $x_i = y_i a_i + b_i$ (e-f in Supplementary Figures S24 and S25).

7. Run the physical transformation once, producing an output 70-D vector $\vec{y}$ (f-g in Supplementary Figures S24 and S25).

8. Add the output $\vec{y}$ to the initial input via a trained skip connection, i.e. $\vec{y} \rightarrow \vec{y} + a\vec{x}$, where a is a trainable skip weight and $\vec{x}$ was the input to the physical system (h-i in Supplementary Figures S24 and S25).

9. Apply trainable, element-wise rescaling to produce the input for the second physical transformation, i.e., $x_i = y_i a_i + b_i$ (i-j in Supplementary Figures S24 and S25).

10. Run the physical transformation once, producing an output 70-D vector $\vec{y}$ (j-k) in Supplementary Figures S24 and S25).

11. Add the output $\vec{y}$ to the initial input via trained skip connection, i.e. $\vec{y} \to \vec{y} + a\vec{x}$, where a is a trainable skip weight and $\vec{x}$ was the input to the physical system (l-m in Supplementary Figures S24 and S25).

12. Sum 50 elements of $\vec{y}$ in sections of 5 to produce a 10-dimensional vector (m-n in Figures S24 and S25).

13. Repeat the above steps for 6 other sub-PNNs, each with its own independent trainable parameters, producing its own independent output 10-dimensional vector (Outputs of each independent sub-PNN shown in o in Supplementary Figure S25).

14. Take a weighted sum of the 7, 10-dimensional vectors, $\vec{y}_{\text{out}} = \sum_{i=1}^{7} w_i \vec{y}_i + b_i$ (p in Supplementary Figures S24 and S25).

To train the PNN, we used PAT with the Adadelta optimizer with learning rate of 0.75, which was reduced to 0.375 after 30 epochs, then to 0.28 after 45 epochs. We found Adadelta to be particularly effective compared to other optimizers, and hypothesize that momentum may inhibit the suppression of gradient errors in PAT, at least in these very small-scale proof-of-concept PNNs. Although we found it resulted in only moderate improvements in small-scale tests, we retrained the digital model for the electronic circuit every two training epochs, using a combination of random 70-dimensional inputs and local data (i.e., the inputs and outputs obtained from the current training data). This was done in part to help compensate for variations in the circuit's response over time, and also to nominally help improve the digital model's accuracy within the local region of parameter space.

3. *Figure 4: Oscillating plate MNIST handwritten digit image classification PNN*

The linearity of the oscillating plate's input-output transformation allowed us to accurately learn even very high-dimensional input-output transformations. There was no need to downsample the original 784-D MNIST images as we could learn a 784-D to 784-D dimensional input-output map. Instead, we could unroll and encode a whole image into a time-multiplexed voltage signal. Outputs of the same dimension were read out from the voltage over time signal of the microphone.

The strong performance of a linear single-layer perceptron on the MNIST dataset and the realization that the oscillating plate PNN performs a matrix multiplication as in Eq. 23 inspired us to use the linear oscillating plate in a similar fashion. By cascading a few layers of element-wise-rescaling and passing the signal through the speaker we roughly emulate the operation of a single-layer perceptron.

The final layer of the oscillating plate consists of reading out a short time window from the microphone output voltage. The exact position and length of the time window was a trainable hyperparameter of our network architecture, however, its position was generally closer to the end of the output as information from the input can only interact with later inputs due to causality. The window is divided into 10 bins. MNIST digits were classified according to which bin had the strongest average signal.

Each inference of the full architecture involves the following steps:

1. 784-D MNIST images are element-wise rescaled, i.e. $x_i \to x_i a_i + b_i$, where a_i and b_i are trainable parameters.

2. Run the physical transformation once, producing an output 784-D vector $\vec{y}$ (Supplementary Figures S27b and S28b).

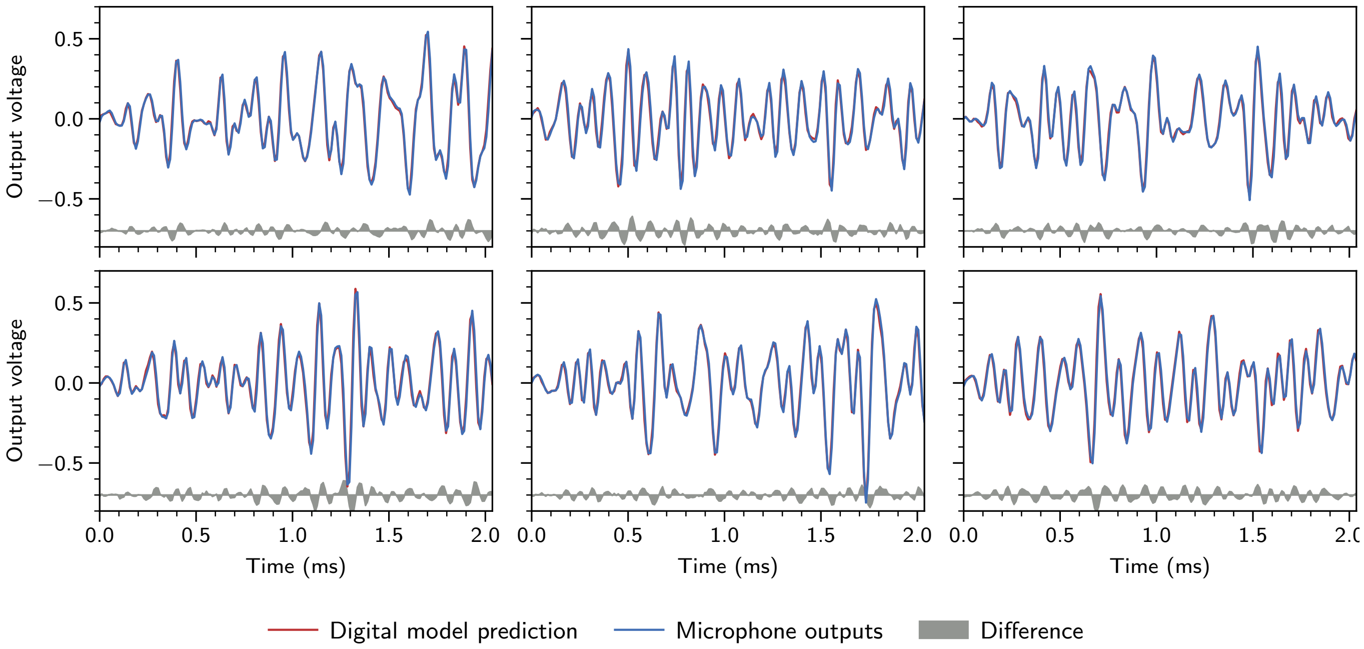

Fig. S26: Multiple microphone recordings for 196 uniformly distributed input voltages at an input frequency of 192kHz and the digital model's predictions. The linearity of the input-output transformation allowed excellent agreement between digital model and experiment as evident from the almost indistinguishable traces.

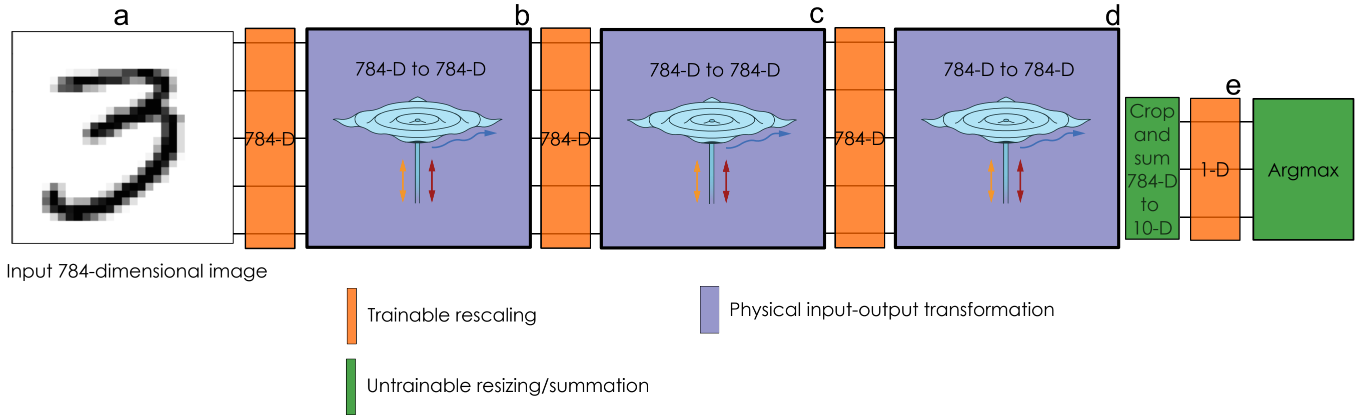

Fig. S27: The full PNN architecture used for the oscillating plate MNIST digit classifier. Annotation letters (a,b,c,d,e) refer to the plots in Fig S28 and the description in the main text. The architecture has a total of $784 \times 2 \times 3 + 1 = 4705$ trainable parameters.

3. Apply trainable, element-wise rescaling to produce the input for the second 784-D physical transformation, i.e. $x_i \rightarrow x_i a_i + b_i$.

4. Run the physical transformation once, producing an 784-D vector $\vec{y}$ (Supplementary Figures S27c and S28c).

5. Apply trainable, element-wise rescaling to produce the input for the third 784-D physical transformation, i.e. $x_i \rightarrow x_i a_i + b_i$.

6. Run the physical transformation once, producing an 784-D vector $\vec{y}$ (Supplementary Figures S27d and S28d).

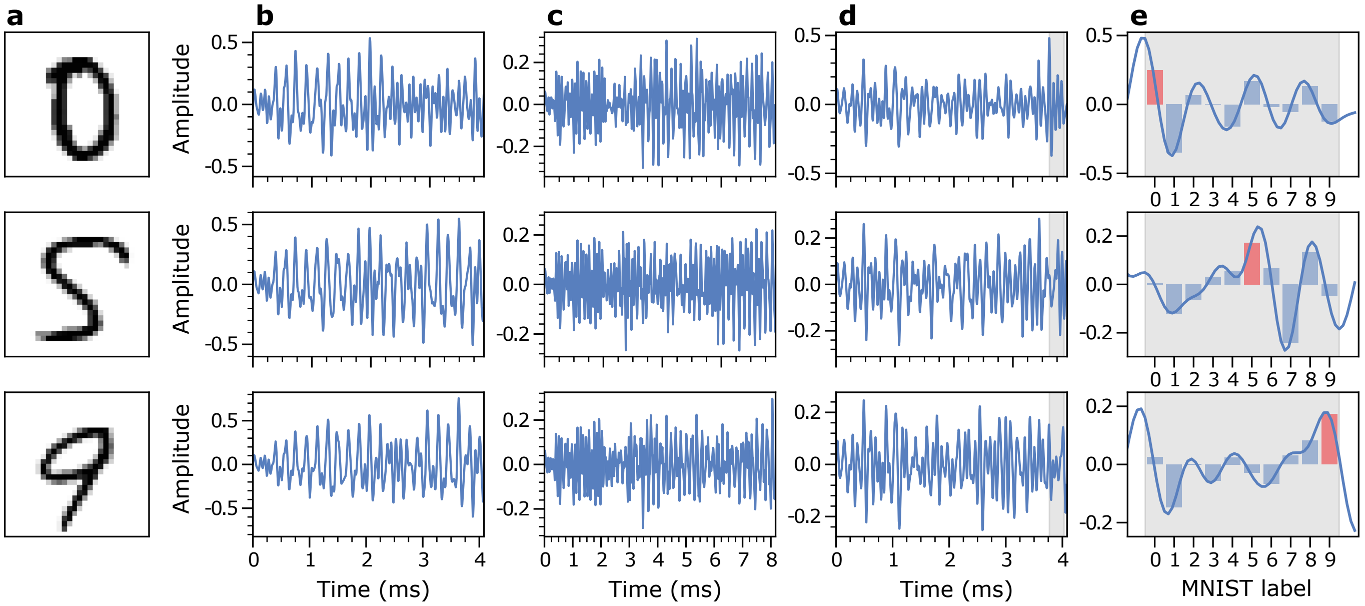

Fig. S28: The sequence of operations that make up the oscillating plate PNN MNIST model. For details of each section, see main text.

7. Take outputs 724 to 774 and average 5 consecutive points to produce a 10-D vector $\vec{y_{\mathrm{out}}}$ (Supplementary Figures S27e and S28e).

Due to the simple PNN architecture and the very accurate linear digital twins, we could achieve high accuracy on MNIST by only using *in silico* training and transferring the trained parameters onto the experiment. Using PAT still resulted in performance gains, although not as dramatic as for the nonlinear physical systems. Usually, *in silico* performance in experiment only fell a few percentage points short of PAT, and after just a few epochs (3) of transfer learning with PAT, the full performance could be retrieved. We expect therefore that, at least for very shallow networks, PAT is less crucial for linear physical systems, and we expect that *in silico* training can be generally applied to greater effect in such systems. While this may be useful in some contexts, we note that sequences of linear systems cannot realize the computations performed by deep neural networks, since they ultimately amount to a single matrix-vector multiplication.

4. Figure 4: Femtosecond SHG MNIST handwritten digit image classification PNN

For the hybrid physical-digital MNIST handwritten digit image classification PNN we present in Fig. 4, we chose to use linear input layers to pre-process MNIST images prior to SHG transformations. The structure of the input layer is in two segments, such that the input dimension is reduced to 50, then transformed back to 196, which is the input-output dimension of the SHG transformation. This seemingly unusual choice was made because we observed that the SHG tended to produce very little change in its output spectrum for high-dimensional random input vectors. Thus, we view the two-step input layer as projecting the input MNIST image into a low-dimensional space of SHG 'input modes', which ideally produces highly variable output spectra. Thus, each of the channels have a different trainable "encoder" (first) linear transformation and a common "decoder" (second) linear transformation that defines the "modes" of the SHG input. We found that, in order to maximize the sensitivity of the SHG's output spectra to the shape of the input modulation, it was optimal to use an effective input dimension of about 10-30 "input modes" (i.e., to choose the input layer to be comprised of two segments, with a middle dimension of 10-30).

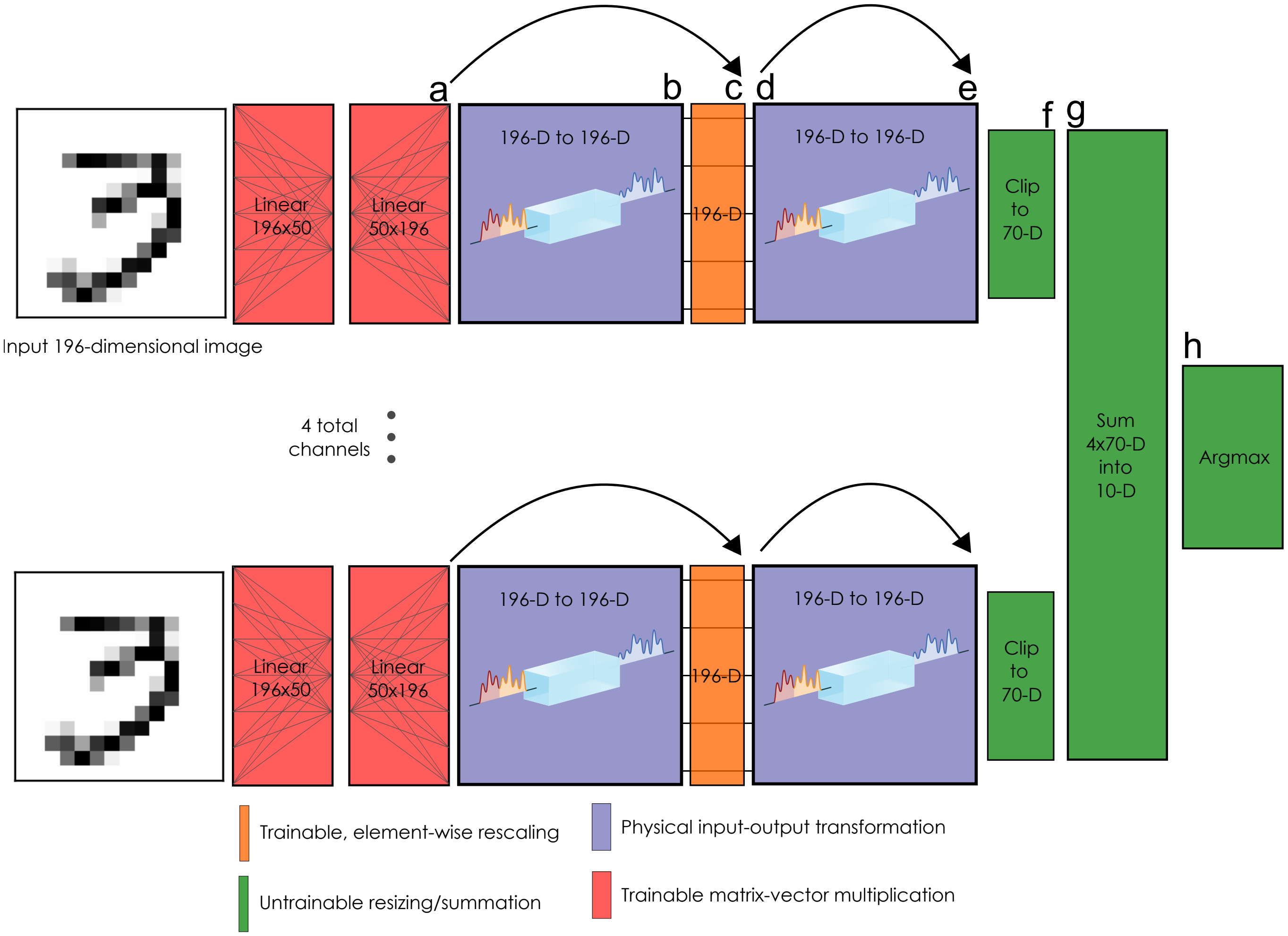

Fig. S29: The full PNN architecture used for the SHG MNIST digit classifier. Annotation letters (a,b,c,d,e) refer to the plots in Fig. S30 and the description in the main text. The architecture has a total of 50,568 trainable parameters. Note that the 50 by 196 trainable matrix-vector operation is constrained to be the same for all channels.

In order to minimize the size of the SHG-MNIST model considered in the main article however, we ultimately increased this input layer dimension to a relatively large value (50) so that good performance could be achieved with relatively few uses of the SHG process. Our desire to keep the model size small (in terms of number of physical system uses) was driven by the relatively slow (200 Hz) speed of the SHG transformation - if the SHG transformation were used many times, it would have been difficult for us to test different configurations. The result of these choices is, however, that the SHG plays less of a role in the hybrid network's calculations than it can with a more optimal choice of input dimension.

Despite this deviation from a more optimal model, the hybrid model presented in Fig. 4 nonetheless leverages the SHG's nonlinear transformations to effectively gain $\sim 7\%$ improvement on the MNIST digit classification task compared to when the SHG transformations are replaced by identity transformations. While this seems like a small improvement, we note that the difficulty of the MNIST digit classification task is highly nonlinear in accuracy: a model reaching roughly 90% needs only $\sim 10^3$ operations, while one reaching 96% or higher may require 100-1000 times as many operations.

Similar to the electronic PNN, we found it was helpful to include skip connections in the network. This is especially helpful because the SHG transformation does not support an identity operation, and is therefore prone to losing

information. This choice also ensured that the input layers could 'choose' to solve the linear part of the classification task without using the physical system, and only rely on the SHG transformations for the more challenging nonlinear parts of the task.

The PNN architecture used for MNIST digit classification with the SHG physical system in Fig. 4 of the main paper is depicted schematically in Fig. S29. The flow of inputs through the network is shown in Fig. S30, where 3 different inputs are shown by different colored-curves. In all three cases, the digits are classified correctly.

Each inference of the full architecture involves the following steps:

1. 196-D MNIST images are operated on by a trained 196x50 matrix, producing a 50-dimensional vector. Without any nonlinear activation, a second trained 50x196 matrix is applied to produce the input to the first SHG transformation (Fig. S30a). This is performed for 4 separate, independent channels (shown in different columns of Fig. S30).

2. The SHG apparatus is executed with the 196-dimensional input, producing a 196-dimensional output vector, which is depicted in Fig. S30b.

3. The output of the SHG transformation is operated on by a trained element-wise rescaling, i.e. $x_i \to x_i a_i + b_i$, resulting in Fig. S30c.

4. To this is added the input to the SHG transformation, multiplied by a trained (1-D) skip weight. This results in Fig. S30d.

5. The SHG apparatus is executed with the 196-dimensional input, producing a 196-dimensional output vector, which is depicted in Fig. S30e.

6. The SHG transformation's output is clipped from index 70 to 140, and added to the SHG input, which is multiplied by a trained skip weight. This produces a 70-dimensional vector depicted in Fig. S30f.

7. The 4 independent channels' output 70-dimensional vectors are concatenated into a 280-dimensional vector, shown in Fig. S30g.

8. The 280-dimensional vector is summed in bins of length 28 to produce a 10-dimensional vector y_{out}.

9. Perform softmax with a trainable temperature T on y_{out} (for training). Perform argmax to obtain the predicted digit (for inference).

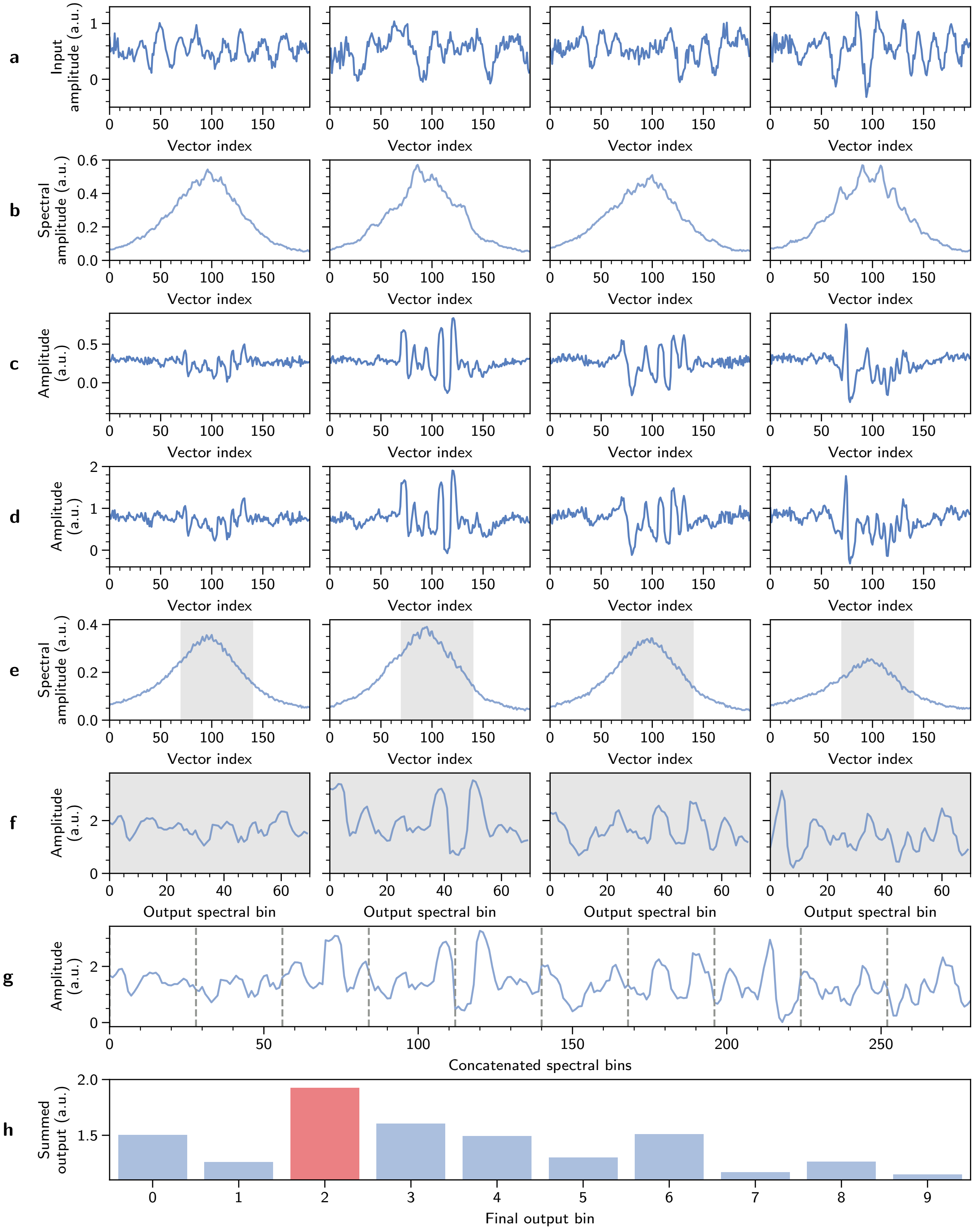

Fig. S30: The sequence of operations that make up the SHG PNN MNIST model. For details of each section, see main text. In parts e and f, the grey section shows the region clipped. In part h, the red-colored bar indicates the correct classification.

3. COMPARISON WITH RESERVOIR COMPUTING

Physical reservoir computing (PRC) is a technique to use generic physical transformations for machine learning [21, 22, 77–82]. In reservoir computers, the physical transformations are untrained (although sometimes hyper-parameters are optimized to improve the quality of the random features produced), and the main form of training is a digital linear regression performed on the physical reservoir output. Given the high-level similarities between physical reservoir computers and physical neural networks (both approaches aim to use arbitrary physical systems to perform machine learning), some readers may be interested to understand how these techniques differ.

Although physical neural networks share physical reservoir computing's philosophy that any complex physical system can be used to perform machine learning, physical neural networks differ from reservoir computers fundamentally. In physical neural networks, multiple layers of controllable physical transformations are trained using backpropagation, similar to how multiple neural network layers are trained together in deep learning. In contrast, in reservoir computers, training occurs only on the output layer.

In this section, we present a comparison of various implementations of feed-forward reservoir computing with the second-harmonic generation (SHG) system to the SHG-based physical neural network presented in Figure 4. Since this section deals only with a specific task (handwritten digit classification), and a specific physical system (ultrafast SHG), it is not exhaustive. Note that we are also comparing reservoir computers operated in a strictly feed-forward manner, which is a common, but not ubiquitous use of the reservoir computing approach – reservoir computers are frequently also employed in a recurrent mode. This is to allow a direct comparison with the feed-forward physical neural networks presented in this work, which are modelled after feed-forward deep neural networks used widely in modern machine learning.

Overall, while not exhaustive, the results of this section suggest physical neural networks can extract significantly more useful computational transformations than physical reservoir computers. Given the enormous energy and speed benefits that physical systems provide (see Section 4), this combination seems likely to be complementary: by applying deep learning's key innovations of backpropagation on multilayer neural networks, physical neural networks can dramatically improve on the machine learning performance of physical reservoir computers. By applying physical reservoir computing's innovation to harness unconventional physics for computation, physical neural networks may allow high-performance machine learning to be performed with energy efficiency and speed far beyond conventional electronic processors.

Figure S31 shows the results of several reservoir computing experiments with the ultrafast second-harmonic generation system (a-f) and, as comparison, the SHG-based physical neural network presented in Figure 4, all for the MNIST handwritten digit classification task.

Figure S31a shows the architecture for the simplest PRC using the ultrafast SHG. Here, the 196-dimensional MNIST image is fed as a vector to the ultrafast SHG experimental apparatus. Note that we found that this specific physical reservoir computer performed better if the MNIST image was inverted (i.e., by pre-processing images as $x \to 1 - x$), so we chose to use the inversion pre-processing for this PRC. The output spectrum is measured to produce a 196-dimensional vector, which is acted on by a trained 196 by 10 linear output layer. Following the approach in Ref. [83], all output layers in this section are trained via logistic regression. When the PRC depicted in Fig. S31a is trained to perform the MNIST digit classification task, the test accuracy is 86% (confusion matrix shown in Fig. S31b). Although this result is not remarkable (a comparable linear classifier can achieve better performance for this task with the same number of trainable parameters), it shows that the SHG transformation has the necessary properties to perform reservoir computing, namely a mostly-deterministic input-output transformation, and features that are sufficiently orthogonal.

To improve the performance of the SHG-PRC, one can increase the number of features provided to the output layer. To do this, it is not sufficient to merely add more independent channels by repeating the design in Figure S31a

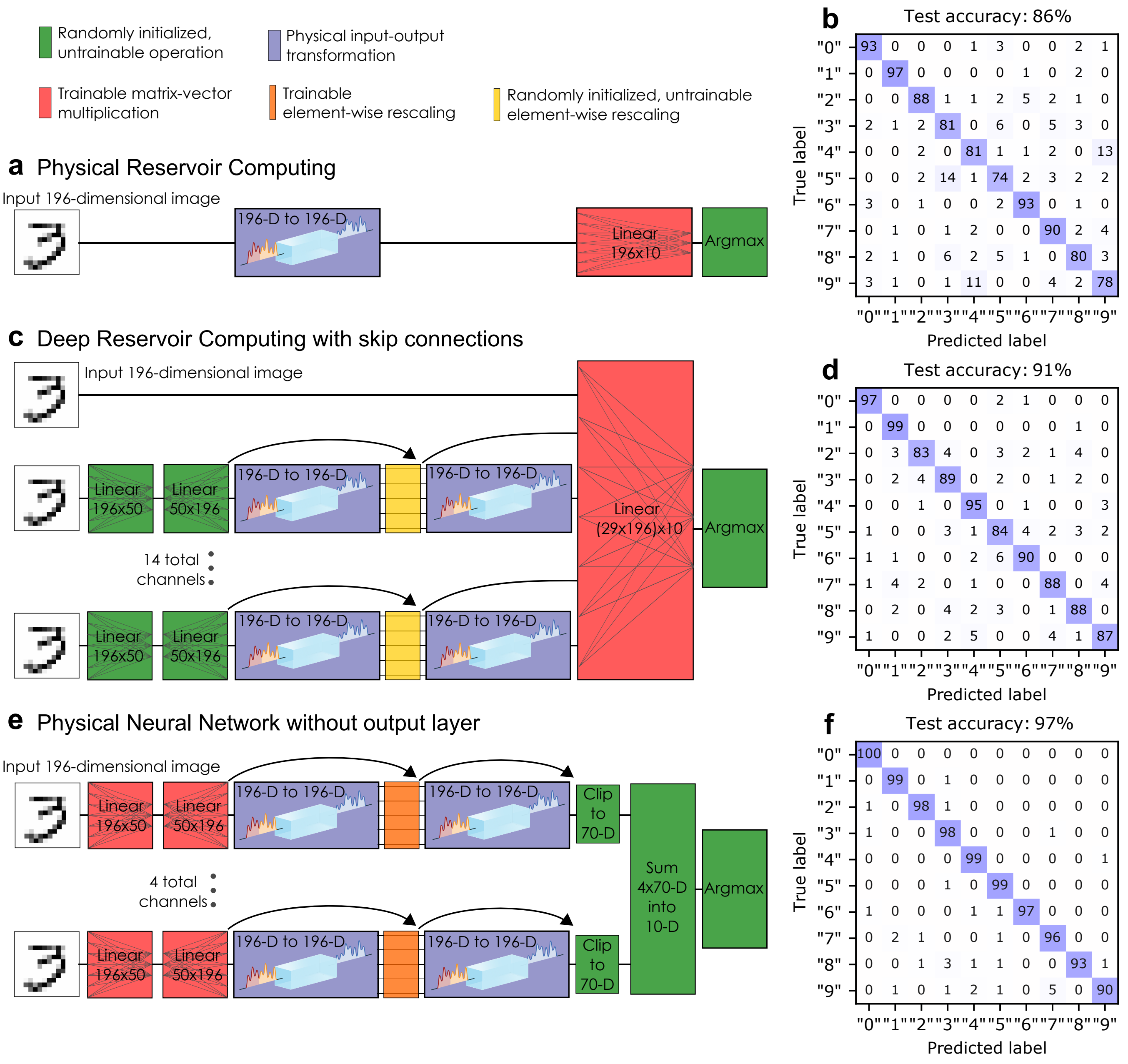

Fig. S31: Comparison between reservoir computing and physical neural networks. **a,c,e,** Schematics of the architectures. **b,d,f,** Resultant confusion matrix for the respective architectures considered. For details of each section, see main text.

and concatenating the features from channels into a single output layer, since the features provided by the different channels would be identical. Instead, we can add random input layers that change the nature of the random features produced by each SHG reservoir channel. Such randomly-initialized input layers are commonly used for PRCs [21]. When this is done, we find that the performance of the multi-channel PRC is, surprisingly, typically worse than the performance of the single-channel architecture shown in Figure S31a. This is because the SHG transformation does not produce useful features if the input vector is highly-structured. We can improve the quality of the SHG PRC's random features by making this input layer a 2-step input layer with a restricted internal dimension, such as a 196

by 50 matrix-vector operation followed by a 50 by 196 matrix-vector operation. This ensures that the vectors sent to the SHG are not too complex, and thus that each SHG reservoir provides more useful features. This rank-constrained input layer was also used in the SHG-based PNN presented in Figure 4, except that the latter was trainable.

To further improve the SHG-PRC, we can make use of two additional design features used in the RC literature. First, we can add to the output features the input vector directly, so that the output layer can solve any linear part of the task directly. Second, we can feed the output of reservoirs into subsequent reservoirs, concatenating the intermediate reservoir features into the output feature vector, as is done in 'deep reservoir computing' [84]. When we do this, we construct a PRC architecture that is very similar to the hybrid digital-physical PNN presented in Figure 4, except that the PRC's input layer and intermediate element-wise rescaling cannot be trained, and the PRC has a trainable output layer with $8 \times 196 + 196 = 1764$ input features, including the input image. When this PRC is trained, it achieves 90.5% test accuracy, which is comparable to a linear classifier, and significantly worse than the SHG-PNN in Figure 4.

However, the above multi-channel SHG-PRC still does not have as many parameters as the SHG-PNN in Figure 4, so for a final comparison we increase the number of channels in the SHG-PRC to 14, such that it has a total of 54,880 parameters. This is more than the number of parameters for the SHG-PNN in Figure 4, which has a total of 50,568 trainable parameters ($196{\times}50{\times}4$ for the first matrix-vector operation in the input layers, 196×50 for the second part of the input layers, which is common to all channels, and $196 \times 2 \times 4$ trainable rescaling parameters). When this 14-channel SHG-PRC, depicted in Fig. S31c, is trained to perform MNIST digit classification, it achieves 91% test accuracy. We found that other measures to improve the performance beyond this point, such as by further modifying the statistics of the randomly-initialized matrices and rescaling parameters, led to only small improvements (up to 1% additional test accuracy) compared to the simpler initialization schemes. Hence, in order to keep the PRCs presented here as close a comparison as possible to the PNN architecture presented in Figure 4, the results in Figure S31c-d show the results obtained with the simpler initialization schemes.

Finally, Figure S31e and f show the architecture for the hybrid physical-digital PNN and its performance. When trained using physics-aware training, the hybrid PNN achieved 97% test accuracy. This architecture features trainable input layers and trainable element-wise rescaling, but does not have an output layer. This choice was made primarily to avoid readers confusing the PNN with a reservoir computer, not because an output layer diminishes performance (rather, we find adding an output layer to the architecture shown in Fig. S31g improves performance by at least an additional 0.5%).

To summarize, we have examined a variety of physical reservoir computing architectures based on the SHG physical transformation, and tried to improve their performance by applying many methods known in the reservoir computing literature. Our results show that, while the SHG physical system can serve as a viable reservoir for PRC, we have not been able to achieve performance that approaches what can be achieved with PNNs, even when the PRC uses more physical transformations, more digital operations, and more digital parameters than the PNN, and even after performing optimization of the PRC architecture to improve the quality of features produced by the physical reservoirs (such as optimizing the input layer rank or statistics, and making use of multiple 'layers' of untrained reservoirs).

This comparison is not exhaustive, in that it only considers one physical system, and one task. Nonetheless, we expect these findings to hold more generally. Feed-forward deep neural networks trained with backpropagation have proven to scale surprisingly well in their performance as the number of parameters is increased, and are now the predominant technique used for large-scale machine learning models (including increasingly on the sequential tasks like language processing that were traditionally performed by recurrent neural networks). Physical neural networks combine the key innovations that have allowed this good scaling to occur, namely the backpropagation-based training of multi-layer computations, with the key insight of physical reservoir computing, namely opportunistic use of arbitrary, energy-efficient and high-speed physical transformations. We anticipate that many of the same physical systems that perform well as PRCs will perform well, and usually better, as PNNs, and that many of the insights developed in

constructing PRCs will be helpful in designing useful PNNs. For additional discussion related to this point, see Sections 4 and 5 of this document.

4. SCALING PNNS AND PAT TO MORE CHALLENGING TASKS: A NUMERICAL EXAMPLE WITH NONLINEAR OSCILLATORS

The experimental physical neural networks we have trained achieve good performance on tasks that, while difficult for early-stage physical hardware, are quite simple for conventional neural networks. To lend more support to the possibility that physical neural networks and physics-aware training (PAT) could one day be useful for accelerating machine learning inference, here we have performed simulations of a physical neural network (PNN), trained using PAT for the Fashion MNIST [56] classification task. Although still a relatively small-scale benchmark task, the Fashion MNIST task is known to be significantly more challenging than the original MNIST task, especially for performance near and above 90%.

Overall, we find that PAT can train a PNN based on a network of nonlinear oscillators to achieve 90% accuracy on Fashion MNIST, as well as 99.1% accuracy on the original MNIST task, in both cases with physical noise included and with over 20% mismatch between the simulated physical system and the digital model used for PAT's backward pass. These results thus show that PAT can tolerate significant model mismatch while still training physical systems to execute relatively complex machine learning tasks accurately.

A. Example system

To perform our tests, we simulated a network of nonlinear, nonlinearly-coupled oscillators, with the following equations of motion:

$$\frac{\mathrm{d}^2 q_i}{\mathrm{d}t^2} = -\sin q_i + \sum_{j=1}^{N} J_{ij}\left(\sin q_j - \sin q_i\right) + e_i \tag{29}$$

where q_i are the oscillator amplitudes, J_{ij} are the coupling coefficients (which must be symmetric, i.e., $J_{ij} = J_{ji}$), and e_i are individual oscillator drives.

Equation 29 is frequently approximated to realize the Frenkel-Kontorova model [85], which is a common model in condensed matter physics. Similar equations describe coupled lasers, electronic or spintronic oscillators, pendula, and, in the continuum limit, nonlinear wave propagation. We chose this physical system for several reasons.

First, it is a generic physical dynamical system that can be scaled simply (by increasing the number of oscillators). Since the system of equations describes coupled pendula, an example considered in many undergraduate mechanics courses, we expect many readers will already be familiar with it. Since coupled oscillators and nonlinear waves are very common, many systems can be compared closely to the system in Eqn. 29.

Second, the physical system's input-output transformation is very different from a conventional artificial neural network layer, but not so different that none of the same intuitions apply. The coupling network provides a large number of trainable physical parameters, J_{ij} and resembles the matrix-vector-product used in many neural networks. However, the nonlinear, sinusoidal form of this coupling is, while very common in physical systems, not typically used in artificial neural networks. Similarly, the couplings in Eqn. 29 are constrained to be symmetric, which is generally not the case in conventional neural networks. Likewise, the sinusoidal self-nonlinearity is common in physics, while most artificial neural networks use non-periodic, sigmoidal or ReLU nonlinearities. Last, and most importantly, Eqn. 29 includes a second-derivative in time. Thus, in stark contrast to well-known neuromorphic dynamical systems like Hopfield neural networks and neural ordinary differential equations [86], this system has many complex phase-space dynamics characteristic of Hamiltonian systems. We find these complex dynamics are actively exploited in the trained PNNs, which suggests that these 'exotic' (to conventional neural networks) dynamics are useful for computing.

B. PNN architecture and details of the physical system

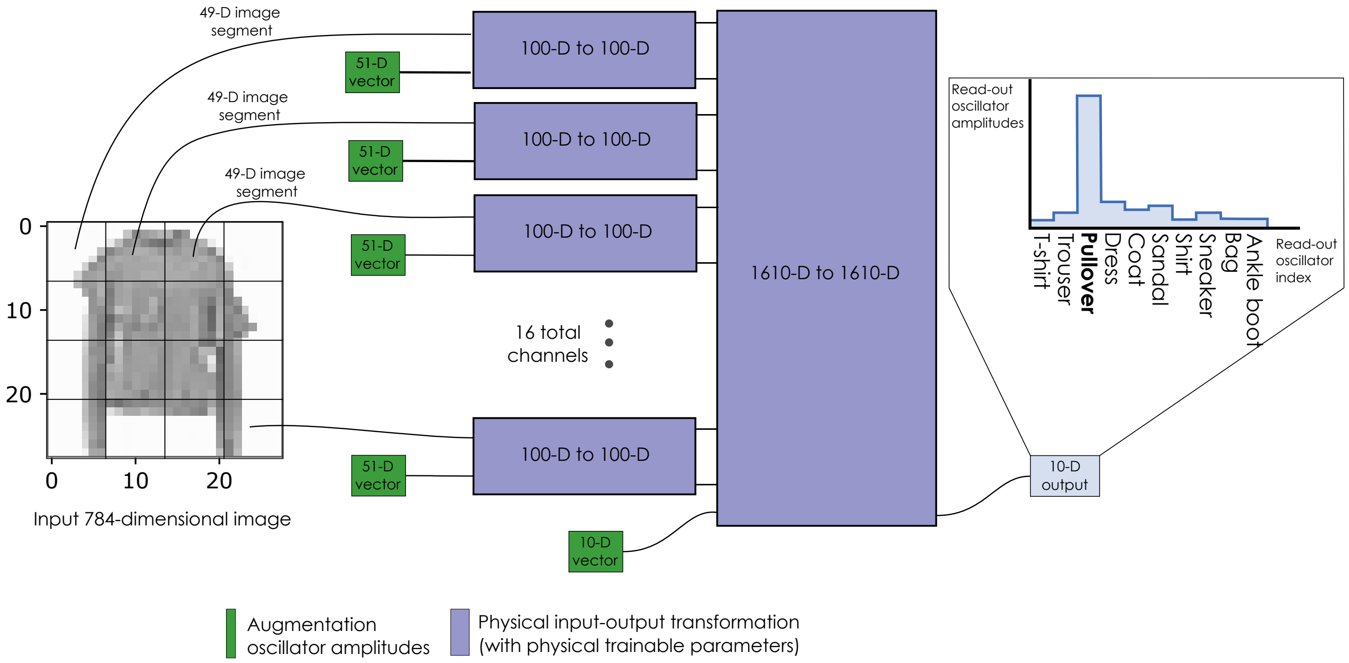

Fig. S32: The architecture for performing MNIST and Fashion MNIST with a PNN based on networks of coupled oscillators. Two different oscillator networks are used in the PNN. The first, smaller network has 100 oscillators and is reused 16 times on different patches on the incoming image, to extract the key lower levels features of the image, similar to convolutional layers. The second oscillator network has 1610 oscillators, and is used as an output layer.

The oscillator system is turned into a nonlinear physical function $\vec{y} = f(\vec{x}, \vec{\theta})$ as follows. The input data is encoded in the initial amplitudes, $q_i(t=0) = x_i + n_i$, where n_i models the noise that would present in setting the initial oscillator amplitudes in an experiment. n_i is sampled from a normal distribution of 2% standard deviation. (Note that we find that the initial oscillator amplitude, $|q_i(t=0)|$, is on average order unity so this does indeed represent about a 2% noise). The trainable parameters of the PNN are the coupling constants between the oscillators, J_{ij}, i.e., $\vec{\theta} = [J_{11}, J_{12}, ..., J_{NN}]$. Because this is a second order ODE, the first derivatives are set to 0, so the system begins at rest $\dot{q}_i(t=0) = 0$. The output vector $\vec{y}$ of the physical function is taken to be the amplitudes q_i after some time evolution T ($y_i = q_i(t=T)$), which we take to be 0.5. As noted above, the J_{ij} are constrained to be symmetric, $J_{ij} = J_{ji}$. In all the oscillator networks considered here, we assume that all J_{ij} can be non-zero (all-to-all connectivity). This is possible in many systems, such as optical networks or electronic oscillator networks (either when connected by a bus or crossbar array), but is not true of many others.

Because capturing locality of the image data is important, we chose the following network architecture. The architecture is schematically depicted in Supplementary Figure S32. The 28x28 (784 pixel) image is divided into 16 patches of 49 pixels each. Each patch is then processed by a small, 100-oscillator network. Though we could have chosen a 49-dimensional oscillator NN to process the data, we choose to use a 100 dimension oscillator NN that is augmented with 51 additional oscillators, initialized at zero, to obtain more complexity from the dynamics. The outputs of all the 16 different patch channels are concatenated into a 1600 dimensional vector. 10 oscillators are augmented to the 1600-dimensional vector. This vector is used as the input to a 1610 dimensional oscillator network. The 10 augmented oscillators in the last layer are taken to be the "class oscillators" which allows us to directly read out the predicted label. The PNN thus has a total of $100 \times 100 + 1610 \times 1610 = 2.6 \times 10^6$ total parameters.

To simulate the effects of the simulation-reality gap and numerically test the performance of physics-aware training (PAT), we modelled the mismatch between the digital model and real physical system present when using PAT by adding noise and physical variations to the simulated physical system (but not the model used for backpropagation) by modifying the dynamical equations as follows:

$$\frac{\mathrm{d}^2 q_i}{\mathrm{d}t^2} = -\sin\left((1+\eta)q_i\right) + \sum_{j=1}^{N} (J_{ij} + J_{ij}^{\text{noise}}) \left(\sin\left((1+\eta)q_j\right) - \sin\left((1+\eta)q_i\right)\right) + e_i \tag{30}$$

where J_{ij}^{noise} is a fixed matrix that represents noise in the coupling and η is a mismatch of the nonlinear coefficient. Each element of J_{ij}^{noise} is sampled from a normal distribution with standard deviation σ. As a reference, note that the trained J matrices are order unity for both small and large oscillator networks. Quantitatively, the RMS values are 0.35 and 1.8 respectively and the average magnitude of each entry of the trained J is 0.26 and 0.25 respectively for the small and large oscillator networks.

Finally, we note that the physical noise that is included in the initial oscillator amplitudes is also considered to be modelled identically in the model used for *in silico* training. Including such a noise model improves *in silico* training relative to the case when physical noise is ignored, but we find it is still insufficient to train the PNN in the presence of a non-negligible simulation-reality gap.

C. MNIST performance

To establish a baseline performance, we first trained a PNN based on the oscillators to perform the original MNIST task. We find that PAT can train an oscillator-PNN to nearly state-of-the-art performance on this task, around 99.1% test accuracy. The confusion matrix for the test set after training the PNN with PAT is shown in Supplementary Figure S33.

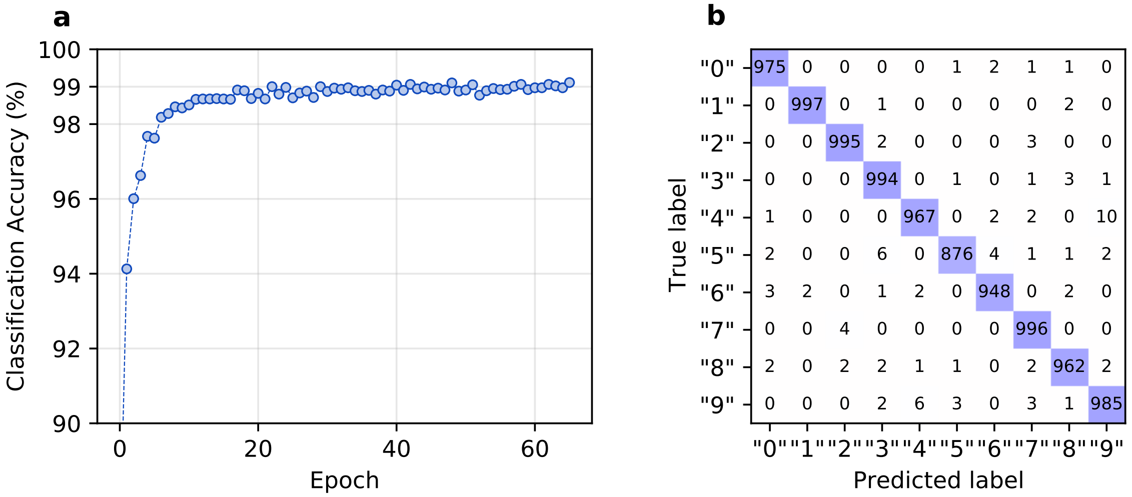

Fig. S33: MNIST classification with the oscillator PNN. Here the oscillator NN has the same architecture as that used for Fashion MNIST in Supplementary Figure S32. The physical system was described by Eqn. 30 with $\eta = 0.2$ and $\sigma = 0.3$. Thus, there is a mismatch between the simulation of the physical device and the model used for backpropagation of approximately 20%. **a** Training curve with physics-aware training. **b** Confusion matrix. The final validation accuracy is 99.1%.

D. Fashion MNIST performance

The Fashion MNIST task [56] is the second task we considered with this new physical system.

Although it still appears relatively simple, the Fashion MNIST task is significantly more challenging than the original MNIST task. As a frame of reference for readers unfamiliar with this task (see `https://github.com/zalandoresearch/fashion-mnist` for a table of performance results with different models):

1. Models that achieve 90% test accuracy or better on Fashion MNIST can usually achieve near state-of-art performance on the original MNIST (99% and higher).

2. Multilayer convolutional neural network models achieving the 90% benchmark typically require around 10^6 to 10^7 multiplication operations.

3. A single-layer perceptron, requiring $784 \times 10 \approx 10^4$ multiplications to execute, achieves around 84% test accuracy on the Fashion MNIST task.

We perform *in silico* and physics-aware training for 3 different values of mismatch between the physical system and the model used for the PAT backward pass and *in silico* training. The physical system is modelled by the mismatched Eqn. 30, while Eqn. 29 is used as the model for the PAT backward pass, and for *in silico* training. As shown in Supplementary Figure S34, PAT succeeds even with very significant model mismatch. With a 20% mismatch for the nonlinear coefficient, and 0.3 standard-deviation coupling noise, PAT trains the PNN to realize 90% validation accuracy, while *in silico* training only achieves around 84%, which is about the performance of a linear model. Even when the model mismatch is increased significantly, to 40% error in the nonlinear coefficient and and coupling coefficient noise standard deviation of 0.7 (corresponding to a mismatch of approximately 70% compared to the RMS of the J matrix), PAT still trains the physical system relatively accurately. Thus, we can conclude that even in the presence of physical noise and extremely large model error, PAT can still train PNNs to accurately perform relatively complex machine learning tasks.

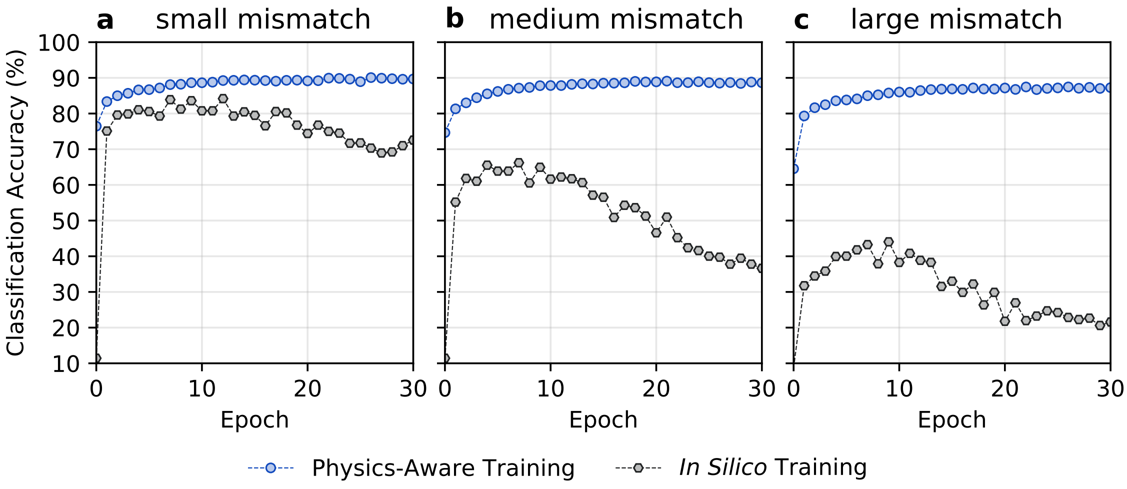

Fig. S34: Comparison of the validation accuracy versus training epoch with PAT and *in silico* training for the oscillator network PNN on the Fashion MNIST task. We consider 3 levels of mismatch between the model used for backpropagation and the model used to simulate physical system. $\eta = 0.2, 0.3, 0.4$ and $\sigma = 0.3, 0.5, 0.7$ for the respective panels in **a-c**.

The confusion matrices after training are shown in Supplementary Figure S36. Supplementary Figure S36a-b show

the result after training with *in silico* and PAT respectively, for the small model mismatch case (approximately 20% difference between model and simulated physical forward pass), while Supplementary Figure S36c-d show the same for the case of medium model mismatch (approximately 30% difference between model and simulated physical forward pass). Here we see that objects that are alike such as a "Shirt" and a "T-shirt" are most likely to be incorrectly classified.

The dynamics of the oscillator networks after training (in the small mismatch case) are shown in Figure S35. It is notable that after training the dynamics include many features that are not easily related to phenomena in standard deep neural networks, such as the anharmonic oscillations in Supplementary Figure S35b, and the dynamic changes in the maximum-amplitude oscillator in Supplementary Figure S35c. Since many of the oscillator amplitudes significantly exceed π during their evolution, the sinusoidal nonlinearity and sinusoidal nonlinear coupling are critical parts of PNN's physical computation.

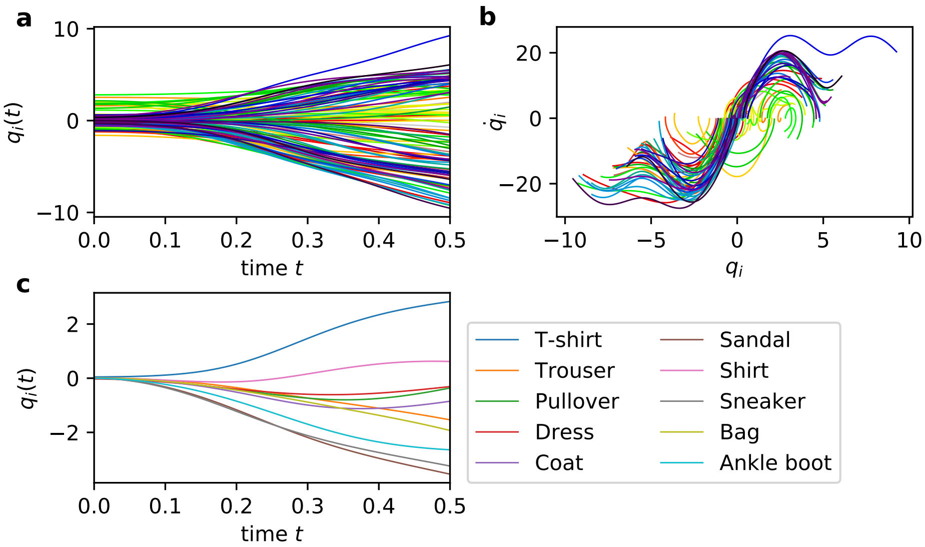

Fig. S35: Sample of the dynamical evolution of the oscillator PNN after training for the Fashion MNIST task. **a,** The amplitudes of the oscillators in the second, 1610-oscillator network (see Supplementary Figure S33) as a function of time. **b,** A phase space representation of the oscillator clearly shows the oscillatory nature of the dynamics. In addition, it also reveals a complex anharmonic nature to the oscillations. **c,** The dynamics of the read-out oscillators. At the end of the evolution, the oscillator corresponding to the "T-shirt" label has the highest amplitude, predicting the correct label.

E. Device-to-device transfer of parameters learned with PAT

Finally, we investigate how sensitive models obtained with PAT are to specific physical parameters, in order to evaluate if models obtained with PAT on one device might be transferable to a different physical device having similar, but not identical parameters (such as two devices made by an imperfectly-controlled fabrication process). To model and quantitatively study this device-to-device model transfer scenario, we assume that the variation between devices is smaller than the variation between the digital model and the physical device. Thus, we consider a second device that has physical parameters perturbed relative to the original device with which PAT was performed. For example, assuming a device-device variation of 30% of the model-device mismatch, the second device is simulated using Eqn. 30, but with the following parameter modifications: $\eta \rightarrow \eta + 0.3\eta$ in the second device, and similarly,

additional noise in the coupling coefficients of the second device such that $J_{ij}^{\text{noise}} \to J_{ij}^{\text{noise}} + J_{ij}^{\text{add,noise}}$, where $J_{ij}^{\text{add,noise}}$ is a matrix with standard deviation 0.3σ. To test transferability of models, we apply the parameters learned by using PAT with the first device to the second device, and then test the accuracy of the second device (without any further training).

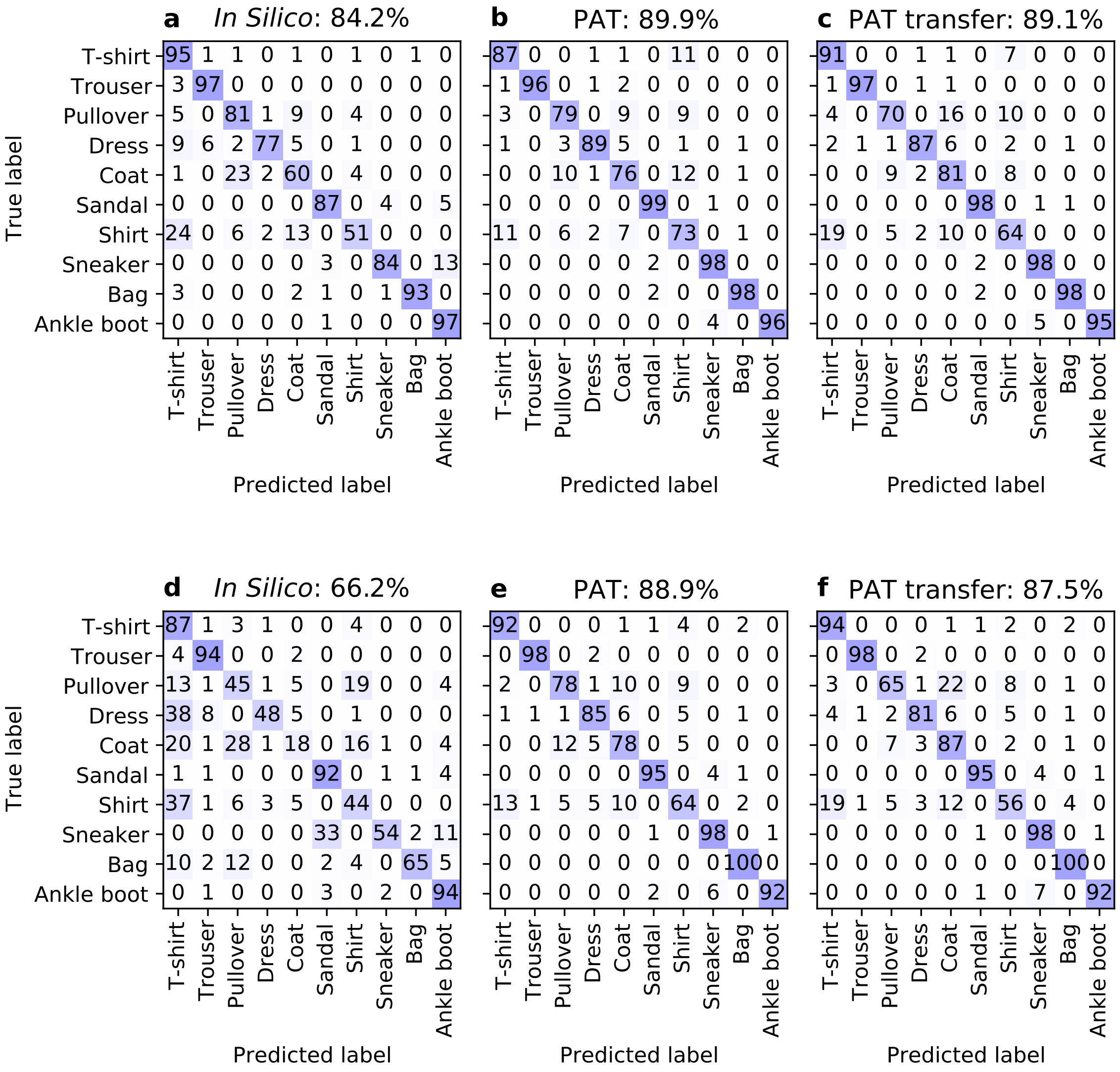

Fig. S36: Confusion matrices showing the classified label predicted by the oscillator PNN versus the correct result for the Fashion MNIST task. **a,b,** Confusions matrices for *in silico* training and physics aware training for the small mismatch scenario considered in Supplementary Figure S34 (about 20% model-device mismatch). **c,** Confusion matrix when the parameters derived from PAT on a first device are transferred over to a second oscillator PNN with variations of about 6% from the first device. **d-f,** The same as **a-c**, but with the model mismatch and device-device variation noise increased to the medium mismatch level considered in Supplementary Figure S34 (i.e., around 30% model mismatch, and 9% device-device variation).

Supplementary Figure S36a and d shows confusion matrices for the first physical device (**a,d**), tested with parameters obtained with *in silico* training, for small (20%) and medium (30%) model-device mismatch respectively. Supplementary Figure S36b and e show the first physical device tested with parameters obtained using PAT. Finally, Supplementary Figure S36c and f show the performance of the second device when it is tested using the parameters

learned using PAT with the first device. Since the device-device variation we consider is a fraction of the model-device mismatch, the device-device variation is about 6% for Supplementary Figure S36c (i.e., 30% of 20%), and about 9% for Supplementary Figure S36f. Overall, the parameters learned by applying PAT to a first device transfer relatively well to a second device, despite non-negligible device-device mismatch, model-mismatch, and physical noise.

Although not shown here, it is of course also possible to use PAT to retrain the second device after transferring the model parameters obtained with the first device. In this case, we find that the original performance obtained with PAT on the first device can be achieved with the second device, but with a much shorter time required for training.

5. CONSIDERATIONS FOR USEFUL PHYSICAL NEURAL NETWORKS BASED ON UNCONVENTIONAL PHYSICAL SUBSTRATES

A. A plausible design workflow for developing PNNs based on unconventional physical substrates

In this section, we present some initial guidelines and advice for designing physical neural networks based on unconventional physical systems. Although our understanding is still preliminary, throughout the course of conducting this work we have recognized a number of design tools and considerations that we expect will be helpful for others who are interested in pursuing research or design along the general direction considered in this work. In short, this section is intended to help readers who may be interested in training a physical system or developing an unconventional physical machine learning platform to get started.

Overall, we recommend the following process for developing physical neural networks based on a given physical system.

1. **First, perform a simulation analysis** to understand the types of computations performed by the physical system, how those computations are adjusted by tunable physical parameters, and when the physical system is capable of providing computational benefits in principle. This technique, which is developed in the next subsection, can help to determine if a given physical system is worth developing into a physical neural network substrate.

2. **Second, perform training of the proposed device using backpropagation *in silico* for representative tasks.** *In silico* training can be helpful to refine the device design by testing various configurations prior to fabrication. Furthermore, since parameters of a device that cannot be reconfigured after fabrication can be, nonetheless, trained *in silico*, this simulation-based training can facilitate pretraining many physical parameters of the device, to maximize its subsequent performance and allow it to be easily retrained for new tasks. The assumption is that these pretrained parameters will mostly be set permanently during fabrication, and thus not accessible to any *in situ* learning algorithm.

3. **Third, perform physics-aware training on the physical device, in order to train reconfigurable parameters.** Since most physical systems will exhibit a simulation-reality gap with respect to known models, physics-aware training will be useful to recover performance expected by *in silico* training in a real fabricated device, and to facilitate transfer learning to new tasks after fabrication. In order to maximize the potential improvement in speed or energy efficiency, the device's reconfigurable parameters should be selected carefully. This is discussed in the last subsection.

4. **Finally, in most applications we expect that physical hardware trained using the methods described above will still need to be augmented by additional hardware, capable of interfacing with and pre- or post-processing digital data, as well as efficient online learning.** In most near-term research, we anticipate this additional hardware will be conventional digital electronics. However, it should ideally be realized using analog neuromorphic hardware capable of in-device learning, so long as the novel hardware is general-purpose, i.e., capable of learning a broad class of functions (this will be the case for hardware in which the analogy to conventional DNN mathematics is relatively strict). As discussed in the last subsection, the distribution of operations among digital, general-purpose-analog, and unconventional physical processors must be considered carefully to ensure the total processor can optimize net energy efficiency and speed.

B. Simulation analysis as a tool for physical neural network design

In this section, we analyze the simulations of candidate PNN systems. The purpose of this section is three-fold:

1. To identify, based on the form of the numerical simulations, what types of mathematical operations candidate physical systems approximate,

2. To determine self-simulation quotients, which estimate an upper-bound on the computational benefits each PNN system could provide, and

3. To analyze the scaling of the latter with respect to physical parameters, in order to guide device design.

Physical processes closely resemble the structure of DNNs that operate on natural data: real physical processes typically feature low-order nonlinearities, hierarchical structure, local correlations, sparsity, and symmetry [36]. DNNs that perform well on natural data either exploit these features in their design (as in convolutional DNNs, which intrinsically account for translational symmetry and locality, or in networks trained with data augmentation to improve robustness), or they acquire it as the result of extended training with natural data. Although DNNs are usually nominally deterministic algorithms, noise like that encountered in real physical systems used for PNNs is often added to improve their performance. DNNs are often trained with data that is distorted or has had noise added to improve the generalization of their models or to reduce sensitivity to adversarial examples. During training, adding noise (such as drop-out) is also common for these purposes. In other applications, such as generative models, noise can play a more fundamental role. Noise within PNNs may be useful for similar reasons, provided it is not too large. In sum, all the reasons DNNs work well for computations involving natural data and natural processes are correspondingly strong rationale for why PNNs should be able to perform many of the same tasks modern DNNs perform.

Physical systems that are good candidates for PNNs implement operations that are well-suited to DNNs, even though they may be more effective for some kinds of computation than others. Such candidate PNN processes implement controllable matrix-vector operations and convolutions, as well as nonlinearities and tensor contractions. In the vast majority of physical systems, arbitrary control over these physically-implemented computations will not be easy. Instead, the physical processes will provide these operations approximately and controllable within some subspace of possible operations (typically subject to sparsity, locality and other symmetries) rather than fully-arbitrary mathematical operations. As a result, although these physical systems may provide significant computational benefits via operations similar to those implemented by modern DNNs, they are unlikely to be useful in isolation, and will sometimes require cooperation from a traditional digital electronic computer, or a general-purpose neuromorphic hardware device. In this case, it becomes essential to keep track of how many operations the PNN is saving the general-purpose hardware - this and related considerations are addressed in the last section of this document.

The self-simulation quotient (SSQ) is the ratio of the cost a given physical system takes to execute (either time or energy) divided by the cost it would take to simulate the physical system using general-purpose hardware. Calculating the SSQs is useful as a 'sanity check': if a physical system's SSQs are not substantially larger than 1 (or is even less than 1), it is unlikely to be useful in accelerating calculations.

The self-simulation quotients of a physical system provide an absolute upper-bound for the possible computational advantages the physical system could provide. The self-simulation quotient of quantum mechanical systems was an inspiration Feynman used motivate quantum computing [87], for example. Since then, experiments which demonstrate that complex quantum systems do indeed simulate themselves much more efficiently than classical computers can have been performed [88, 89]. These demonstrations, and the rationale behind them, do not guarantee that large quantum systems will be useful for actual, practical computations, but they do provide assurance that computational advantages are possible in principle. Even though we are interested in physical neural networks described by classical physics in

this work, our motivation for examining the self-simulation quotient is similar. SSQs for classical systems scale less rapidly with system size than quantum SSQs, but it is also much easier to scale and build classical systems. We find that the SSQs for classical systems can be extremely large - suggesting (though certainly not guaranteeing) that there is plenty of potential for accelerating computations.

We find that, generally, self-simulation quotients are higher when the physical system is intuitively more complex. Concretely, this corresponds to systems being designed such that:

1. Nonlinearity is present, especially higher-order nonlinearities and those that couple multiple degrees of freedom.

2. All degrees of freedom are coupled in heterogeneous ways to many other degrees of freedom.

3. The dimensionality is high (e.g. 3-D versus 2-D). However, this will often correspond to a reduced nonlinearity.

However, merely possessing large SSQs isn't sufficient for a physical system to be useful to speed-up or increase the efficiency of practical calculations, since the operations performed during self-simulation may not resemble any useful machine learning calculation. Instead, one needs large SSQs *and* the system's computations to translate well to performing all or part of useful calculations.

One conservative approach to ensure that a physical system's natural computations translates to machine learning is to choose physical systems whose evolutions closely resemble standard artificial feedforward neural networks. This can be seen as a weak form the usual strict mathematical analogy which most DNN accelerator hardware aim to maintain. For example, layer-by-layer 'propagation' through residual feedforward neural network layers [90] follows a form that resembles the Euler integration of a differential equation:

$$x_i[t+1] = x_i[t] + (f_i[t] + \sigma\left(\sum_{j=1}^{N} a_{ij}[t]x_j[t]\right), \tag{31}$$

where $\sigma(...)$ is a nonlinear activation function, such as tanh, and $f_i[t]$ is a bias. As is exploited in neural ordinary differential equations [86], this corresponds to a continuous dynamical evolution:

$$\dot{x}_i(t) = \sigma\left(\sum_{j=1}^{N} a_{ij}(t)x_j(t)\right) + f_i(t). \tag{32}$$

Since physical systems mostly do not follow dynamics of this precise form (none of the systems considered in this work do), and because systems evolving with more nonlinearity seem to offer larger potential for physical speed-up (at least as suggested by the SSQ), it will nonetheless be important to explore physical systems that differ from Eqn. 32, both slightly and significantly. We have already found that systems that do not resemble conventional neural networks can nonetheless perform deep learning calculations, and our explorations are still very early. It may turn out that such explorations discover not only useful physical systems for machine learning, but also useful alternative algorithms and mathematical ansatzes for conventional deep neural networks.

1. *General case*

The findings summarized above can be appreciated intuitively by considering a coupled nonlinear differential equation model of a physical dynamical system with N distinct degrees-of-freedom (modes) x_i, which interact with up to nth-order nonlinearities, and are driven by N driving forces $f_i(t)$

$$\dot{x}_i(t) = \sum_{j=1}^{N} a_{ij}(t)x_j(t) + \sum_{j=1}^{N}\sum_{k=1}^{N} b_{ijk}(t)x_j(t)x_k(t) + \sum_{j=1}^{N}\sum_{k=1}^{N}\sum_{l=1}^{N} c_{ijkl}(t)x_j(t)x_k(t)x_l(t) + ... + f_i(t). \tag{33}$$

For such a system, if the coupling of the N degrees of freedom is heterogeneous and non-negligible over M neighbours for all orders of nonlinearity, then the simulation complexity is dominated by the highest-order nonlinear term as $\Delta T \Delta f N M^n$, where Δf is the bandwidth of the system (the inverse of the necessary time step) and ΔT is the simulated time. Note that, because sparsity, locality and other symmetries can be exploited to make each term faster to simulate, it is important to consider the coupling range M of heterogeneous, non-negligible coupling.

In most systems, the coupling range depends on the order of the nonlinearity, so a more general expectation is

$$\text{Simulation Complexity} \sim \Delta T \Delta f N Q_{\max}, \tag{34}$$

where $Q_{\max} = \max[M_i^i]$ is the maximum coupling complexity over the different terms, where M_i is the coupling range of heterogeneous coupling for the ith order nonlinear term.

The power required to sustain such a physical system will usually be linear in the number of modes N, and the time taken is of course ΔT, so the most important factor in the scaling of self-simulation quotients is $Q_{\max}$: more, and more complex, coupling between physical degrees of freedom leads to larger SSQs. This however assumes that n and M are independent of the power supplied to the physical system, which will generally not be true: in most systems, as the energy localized in the system is increased, more nonlinearity and more complex, long-range coupling are necessary to describe the physics. As the dimension of the dynamics increases, the number of neighboring regions to any point increases: the ability for localized degrees of freedom radiating a fixed amount of energy to couple with neighbours increases exponentially. This is intuitive: higher-dimensional physics is harder to simulate.

Equation 33 also identifies, very generically, some of the physical data transformations realized by physical systems. If the physical parameters of the system that determine a_{ij}, b_{ijk}, c_{ijkl} can be adjusted and trained, then the evolution of the $x_i(t)$ implement matrix-vector operations (first term) and higher-order tensor contractions (subsequent terms). Depending on the locality and symmetry of a_{ij}, b_{ijk}, c_{ijkl}, these operations will often be similar to convolutions. In general, these coefficients could also be controlled in time, $a_{ij}(t)$, $b_{ijk}(t)$, $c_{ijkl}(t)$. While this approach allows more complex sequences of operations to be controllably executed by a single physical system, it may not be compatible with in-place parameters (parameters which are stored in-place in the physical device, and therefore do not need to be retrieved from a digital memory each time an inference is performed). For this reason (see the last section of this document), we expect the most useful, scalable parameterization of most physical systems will be the use of trainable, but time-independent physical parameters a_{ij}, b_{ijk}, c_{ijkl}, with possibly a relatively small number of time-dependent parameters.

Alternatively, if some of the $x_i(t)$ are controllable parameters (i.e., are treated as forced source terms, rather than terms obeying Eqn, 33), then one may recognize the nonlinear terms as realizing tensor operations of order n, where n is the order of nonlinearity (for example, the quadratic term $\sum_{j=1}^{N}\sum_{k=1}^{N} b_{ijk}x_jx_k$ could result (in part) in a controlled matrix-vector operation if some of the $x_k(t)$ are controlled parameters). If the drive terms $f_i(t)$ are used as controllable parameters, the outcome will probably be similar to controlling $x_i(t)$, depending on the memory of the system's dynamics, and the strength of the drive. For the same reasons mentioned in the last paragraph, such time-dependent physical parameters may pose scaling challenges if they require fast reading from a digital memory, and digital-to-analog conversion.

This general model also suggests that the greatest physical advantages can be gained with operations that are typically not used in modern deep neural networks, namely high-dimensional tensor operations, which are associated

with higher-order nonlinear coupling. Of course, this depends on utilizing these operations efficiently to perform machine learning, which has not been widely studied.

In the rest of this section as follows, we will analyze the physical costs (energy and time) to evolve two realistic PNN modules based on different physical systems, then compare these physical costs to those incurred by performing a simulation with a state-of-the-art digital electronic device. For these comparisons, we will consider the Nvidia DGX Superpod supercomputer [91] which achieves 39 pJ per floating point operation and 2.8×10^{15} operations/s, and the Nvidia Titan RTX GPU [92], which achieves 9.8 pJ/operation and 32×10^{12} operations/s for half-precision operations.

2. Nonlinear optical pulse propagation in one dimension

If we assume a third-order nonlinearity, nonlinear optical pulse propagation in one spatial dimension, z, evolves along z according to the nonlinear Schrödinger equation:

$$\partial_z A(t,z) = i\gamma|A(t,z)|^2 A(t,z) + \frac{i\beta_2}{2}\partial_{tt} A(t,z). \tag{35}$$

Studying a second-order nonlinear interaction, as considered in the main article, would involve a similar formulation, but since the NLSE is a much more well-known model for nonlinear wave propagation, we consider it to be better-suited to this example.

As written, Eqn. 36 does not closely resemble a conventional neural network. However, if an additional term, corresponding to a spatially or temporally-varying refractive index modulation is included on the right-hand side, $i\delta(t,z)A(t,z)$, then an approximate analogy can be established[14]. In this case, the combination of the dispersive term on the right-hand side of Eqn. 36 with the modulated refractive index $i\delta(t,z)A(t,z)$ can be seen as realizing a constrained linear coupling between electric field envelope amplitudes in time, roughly analogous to the matrix-vector product or linear convolutions in conventional artificial neural networks.

Alternatively, if the equation is considered in the frequency domain, the Kerr nonlinearity implements a constrained tensor contraction between frequency components of the input field envelope, $\tilde{A}(f,z)$. For simplicity, let us neglect the linear dispersive term, and suppose that the different frequency components are expressed as discrete frequency modes, $\tilde{A}_i$. Then, the nonlinear evolution is:

$$\partial_z \tilde{A}_i(z) = \sum_j \sum_k \sum_l \Gamma_{ijkl} \tilde{A}_j(z) \tilde{A}_k^*(z) \tilde{A}_l(z) \tag{36}$$

where Γ_{ijkl} is a tensor that ensures energy conservation.

Clearly, this evolution does not resemble a standard feedforward neural network. Suppose, as in our example second-harmonic PNN, that part of the input pulses' frequency components are assigned to trainable parameters, via a parameter field w, and the remainder is used as the data input, s. Then, the complex sum above will include some trainable terms in which s_i is linearly coupled to other s_j, such as $\Gamma_{ijkl} w_j(z) w_k^*(z) s_l(z)$, as well as controllable nonlinear terms, such as $\Gamma_{ijkl} w_j(z) s_k^*(z) s_l(z)$. Since the frequency components $w_i(z)$ evolve nonlinearly, and dispersion will generally complicate this description further, it suffices to say that there is not a straightforward analogy of this physical evolution to a conventional artificial neural network.

Let us now evaluate the self-simulation quotients. For ultrafast pulse propagation in nonlinear dispersive media, a standard simulation algorithm is the split-step Fourier method [93]. We will use the computational complexity of this algorithm as our basis for assessing self-simulation quotients.

For an optical propagation length L, the split-step algorithm divides the propagation into small steps whose length must be much shorter than the rate at which nonlinear effects occur, L_{nl}. The pulse is described by a vector of

N_ω values which contain the complex amplitudes of the electric field envelope. The number of spectral points is determined by the maximum frequency range of the pulse and any new frequency content the propagation creates, and by the time window needed to describe the pulse throughout the propagation, $N_\omega = T\Delta\omega$, where $\Delta\omega$ is the bandwidth of the simulation and T is the required time window of the simulation.

In the split-step algorithm, linear effects like dispersion are efficiently applied in the Fourier domain. The complexity of the split-step algorithm is dominated by the Fourier transform operations required, so:

$$N_{\text{ops}} \sim N_z N_\omega \log(N_\omega), \tag{37}$$

where N_z is the number of longitudinal steps required. The minimum step size is proportional to the rate of nonlinear effects, $N_z = L \times \gamma f(P)$, where $f(P)$ is the dependence of the nonlinear rate on the peak power P of the pulse and γ is the nonlinear constant, which depends on the material, the type of nonlinearity, and the transverse confinement of the single spatial mode the pulse propagates in. For Kerr (cubic) nonlinearity, $f(P) = P$, whereas for quadratic nonlinearity $f(P) = \sqrt{P}$, generally $f(P) = P^{(n-1)/2}$ where n is the order of the nonlinearity.

The peak power P is related to the minimum temporal feature of the pulse, $\sim 1/(\Delta\omega)$, where $\Delta\omega$ is the bandwidth of the simulation. $N_\omega = T\Delta\omega$, where T is the required time window of the simulation. In other words, N_ω is the maximum time-bandwidth product that occurs during the propagation, which generally increases for more complex, dispersive and nonlinear pulse propagation. For the sake of simplifying the discussion, we will let $P_0 = E_p N_\omega$, though we note that this will often overestimate the nonlinearity.

The maximum rate at which physical computations can be performed is determined by the longest of the following: duration between input light pulses, duration between electro-optic modulations, and the maximum duration of the optical pulses during propagation, with the last required to ensure that each physical computation is independent (which is usually, but not always required for classification tasks). We will assume this corresponds to about 33 ps (so a rate of 30 GHz). Pulsed sources of this repetition rate are possible [94], and electro-optic modulators with bandwidths in excess of 40 GHz are standard. Provided the length of the nonlinear optical medium, and the dispersion and pulse spectral broadening are chosen so that the maximum pulse duration never exceeds 33 ps, 30 GHz operation should therefore be feasible.

The self-simulation quotient for time is then:

$$\text{SSQ}_t = \frac{\text{Time for simulation}}{\text{Time for physical evolution}} = \frac{t_c N_{\text{ops}}}{t_p}, \tag{38}$$

where t_c is the time per operation for the reference conventional computer, and t_p is the time the physical evolution takes (being limited by one of the above considerations).

Similarly, energy:

$$\text{SSQ}_e = \frac{\text{Energy for simulation}}{\text{Energy for physical evolution}} = \frac{e_c N_{\text{ops}}}{E_{\text{phys}}}, \tag{39}$$

where e_c is the energy per operation of the reference computer and E_{phys} is the total energy required to instantiate and measure the pulse propagation. Instantiating the pulse propagation and measuring its result requires significantly more energy than just that needed for the optical pulse, as an example will show.

Quantitative calculations with plausible devices. Building upon these scaling expressions, we calculate the energy and speed self-simulation quotient of a realistic, scaled device based on ultrafast nonlinear pulse propagation. Our intention here is not to design a device that would maximize the self-simulation quotient within the most optimistic conditions, but to consider a realistic (though not trivial) experimental demonstration possible with existing technology. Throughout, we will note room for improvement in near-future devices, and at the end analyze both the realistic present-case and near-future potential.

We consider a device as follows. An ultrashort pulse at 1550-nm is split into 20 wavelength bins, where it can be modulated by electro-optic amplitude modulators and then recombined. Through these electro-optic modulators, input vectors can be applied at $\sim$ 10-100 GHz via spectral modulations. For physical parameters, the pulse is then shaped by a phase pulse shaper based on a spatial light modulator or similar device. This device allows $\sim 10^3$ or more independent controllable parameters for the subsequent nonlinear pulse propagation, which can be stored in-place over many uses of the PNN without incurring energy costs due to memory reads or digital-analog conversion. The modulated pulse then propagates in a nanophotonic waveguide based on periodically-poled thin film lithium niobate. To maximize the complexity of the dynamics, we assume the waveguide is poled to create an artificial Kerr effect, so that it can be roughly described by Eqn. 36 above.

In Ref. [95], 1 pJ pulses were sufficient to observe significant supercontinuum pulse propagation in a cm-scale waveguide. However, the pulses used were simple, transform-limited pulses. To ensure very complex pulse propagation with the structured, data-encoded pulses used for computation, we assume that roughly 50 times as much pulse energy should be used (spread across a longer, more complex pulse). To account for various optical losses in a realistic device (transmission around 1%), and the wall-plug efficiency of the initial mode-locked laser (around 10%), we assume a total of 50 nJ of energy is required per optical pulse. This energy could be reduced by several orders of magnitude in a fully-integrated device, since the optical losses would be drastically reduced. Assuming a 0.5 cm waveguide, the pulse duration should remain below 33 ps throughout the waveguide (for a dispersion of -15 fs^2/mm, such a waveguide would correspond to at most 10-20 dispersion lengths, so a maximum duration of 10-20 ps is reasonable to expect given the expected pulse bandwidths described below).

To inject data to the physical system, it must be converted from a digital computer's digital format to a format (typically analog) suitable for the physical system. The most energy-efficient ADCs are capable of approximately 1 pJ/conversion [96] at 24 GHz. For the sake of this order-of-magnitude estimate, we will assume ADC and DAC operations each require 1 pJ/conversion. Integrated electro-optic modulators that operate with $\sim$ pJ/modulation are typical [97–99], while modulators capable of few-bit-depth modulation appear possible [99–101]. For our purposes here, we will assume each fast optical modulation requires an additional 1 pJ on top of the DAC cost. Although on-chip photodetectors with ultralow capacitance may enable amplifier-free detection of light pulses as weak as 1 fJ [102, 103], we will assume that detectors add an additional 1 pJ on top of the ADC cost for a modern experiment. Finally, the spatial light modulator requires roughly 10 W to maintain, resulting in 330 pJ per 33 ps inference. Of course, in future devices this would be significantly reduced, presumably to significantly below 1 W.

Adding these physical costs per inference is:

$$330 \text{ pJ} + 20 \times 1 \text{ pJ} + 20 \times 1 \text{ pJ} + 20 \times 1 \text{ pJ} + 50 \text{ nJ} = 50.39 \text{ nJ} \tag{40}$$

for the present-day experiment, and

$$33 \text{ pJ} + 20 \times 0.1 \text{ pJ} + 20 \times 0.1 \text{ pJ} + 20 \times 10 \text{ fJ} + 0.5 \text{ nJ} \approx 537 \text{ pJ} \tag{41}$$

for an improved, near-future device. For both devices, the time-per-computation would be limited by the propagation time, 33 ps, since fast electro optic modulators and DAC/ADC would permit input/output rates exceeding 1/(33 ps) = 30 GHz.

We now consider the energy and time costs for simulating this system with state-of-the-art conventional hardware. For the 50 pJ pulses we have assumed, if the waveguide is designed to support weak anomalous dispersion, peak powers of roughly 1 kW ($\sim$ 10 pJ localized into a 10 fs region) are probable, corresponding to a nonlinear length scale of 10 µm for the device in Ref. [95] which has an effective self-phase modulation coefficient of 100/Wm. To adequately simulate this would require a longitudinal step size of around 1 µm or smaller. To accommodate spectral broadening up to around 400 THz, a spectral range $\Delta\omega$ of 800 THz is required for accurate simulations. To accommodate the

complex shaped pulses and dispersion of the broadband light, a temporal window size of 10-50 ps would be expected, since the initial pulse duration would be roughly 1-5 ps depending on the spectral modulations applied (100-1000 phase degrees of freedom across a $\sim$ 50 THz input bandwidth), and the 5 mm waveguide corresponds to roughly 5-8 characteristic dispersion lengths for the broadened pulse (assuming a dispersion of -15 fs^2/mm).

Thus, using the Fourier split-step method, the number of operations for simulations would be:

$$N_z N_\omega \log_2(N_\omega) = (5\text{ mm}/1\,\mu\text{m}) \times 800\text{ THz} \times 25\text{ ps} \times \log_2(800\text{ THz} \times 25\text{ ps}) \approx 5 \times 10^8 \text{operations.} \tag{42}$$

Using the Nvidia Titan RTX, this would cost 5 mJ and take 16 µs. This corresponds to roughly 10^5 times more energy and 10^5 times longer than the present-day experiment, and roughly 10^7 more energy and 10^5 times longer than the near-term device.

Using the Nvidia DGX SuperPOD, the simulation could be performed more quickly, 179 ns, but with greater energy cost, 20 mJ. This corresponds to roughly 10^5 times more energy and 10^4 times longer than the present-day experiment, and roughly 10^8 more energy and 10^4 times longer than the near-term device.

Although the self-simulation quotients possible with nanophotonic nonlinear optics are large, as was mentioned at the start of this section, this only provides a first clue that nanophotonic nonlinear optics could be useful for machine learning. Our simulation analysis has identified several important considerations, which should be taken into account alongside the SSQs.

First, the scaling of the self-simulation quotient for single-mode nonlinear optical pulse propagation is in general only marginally superlinear with respect to the optical degrees of freedom, N_ω, since the logarithmic factor is relatively weak. Since larger N_ω can give rise to shorter pulses, in some regimes N_z could scale optimistically as N_ω, suggesting a best-case scaling of $N_\omega^2 \log_2(N_\omega)$. It is not obvious if pulse propagation that supports this complexity scaling will also be useful for practical computations, however.

Second, the operations performed by single-mode nonlinear optical pulse propagation are, by default, not well-matched to the standard operations performed in modern deep neural networks, rather they are constrained, high-order tensor contractions. Because of energy and momentum conservation in these nonlinear interactions, it will rarely be possible to realize fully arbitrary high-order tensor contractions or convolutions. While PAT and PNNs can facilitate training physical computations based on these exotic operations (this was one scientific motivation for using nonlinear optics as a first demonstration of PNNs here), it is not yet clear how those PNNs should be designed to be useful, since machine learning based on their underlying operations is not well-studied.

To make the nonlinear pulse propagation more closely resemble a conventional neural network, one could include a time or space-dependent refractive index modulation, though this would entail a more complex device, with possibly higher energy costs, and moreover the type of linear coupling achieved would be subject to physical constraints that could limit the types of functions that could be efficiently approximated, compared to a general-purpose device.

In summary, while single-mode, nanophotonic nonlinear optics presents many promising features for PNNs, we believe that significant effort will be required to understand how to utilize the exotic nonlinear optical operations in practical tasks. Work along this direction is promising [14, 104, 105]. In contrast, other systems, such as the electrical networks discussed subsequently, have a more direct connection to standard deep neural network operations.

3. *Analog transistor networks and similar systems*

As a second example, here we consider (at a very coarse level of detail) the transient, analog nonlinear dynamics of a network of transistors.

To describe the transient analog nonlinear dynamics of a network of transistors, one may use a nonlinear circuit simulation strategy, solving a system of nonlinear differential equations of at least size $N_T N_d$, where N_T is the number

of transistors, and N_d is the number of dimensions (∼nodes) in the equivalent circuit model of the transistor. For accurate large-signal analog models such as the EKV model, $N_d \sim 10$. To solve for the dynamics of a nonlinear network of transistors, a common technique is to numerically integrate the differential equations that describe the network by solving at each time-step the nonlinear system of equations using a nonlinear solver such as Newton-Raphson. Alternatively, if we can approximate all nonlinearities by low-order Taylor-series approximations, one may instead integrate a system of coupled nonlinear differential equations directly. Since the nonlinearities in electronic circuits are typically strong, including strong saturation and exponential dependencies, the latter approach is not often used.

The standard approach scales in complexity as $\mathcal{O}(n^{1.3-1.8})$ [106–108], with the most complex step due to the linear system solving required for each iteration of the Newton-Raphson method. For simulating very large circuits, there are methods to improve this scaling depending on the topology and details of the circuit [107, 108]. However, these methods sacrifice accuracy, and depend on the specific circuit topology.

Even though it is less accurate, integrating a system of equations similar to Eqn. 33 allows more straightforward insight into the circuit simulation scaling and the operations it effectively performs. Thus, even though our estimate of the self-simulation quotients will be less accurate, we will be able to more easily realize the more important goal of understanding the computations the physical system can perform.

Assuming integration of the nonlinear system of differential equations, the number of operations to simulate the transistor network is nominally:

$$N_{\text{ops}} \approx \Delta T \Delta f [N_T N_d M_1 + N_T N_d^2 M_2^2 + ... + N_T N_d^n M_n^n], \tag{43}$$

where ΔT = time of simulation, Δf = bandwidth of simulation (the inverse of the time-step, roughly the maximum frequency expected to be encountered in the dynamics), N_d = number of degrees of freedom needed to describe each transistor ∼ 10-20, n = highest polynomial order of the nonlinear coupling needed to describe the operating regime, and M_i = range of transistor-transistor coupling (i.e., the number of other transistors each transistor is non-negligibly coupled to) for each order of nonlinearity.

Although it may not be true when nano-sized transistors are operated near one another at radio frequencies, we will assume that the nonlinear terms are local to each transistor, so $M_i = 1$ for i larger than 1. In this case,

$$N_{\text{ops}} \approx \Delta T \Delta f [N_T N_d M_1 + N_T N_d C_{\text{nl}}], \tag{44}$$

where we have assumed that a more efficient method is used to calculate the nonlinearity at each transistor, such that the scaling is only a constant factor larger than the number of degrees of freedom required to simulate each transistor. We have done this because there are obviously much more efficient methods for the transistor nonlinearity than the full ODE expansion in Eqn. 33, and we would prefer to obtain the most conservative SSQ estimates possible. If we let $C_{\text{nl}} = N_d$, it is equivalent to assuming that the calculation of the nonlinearity is about as efficient as if the nonlinearity were approximated by terms up to quadratic. Provided C_{nl} is not much larger than M_1, its choice is not significant in our final estimate, since the nonlinear part of the simulation will be comparable to or smaller than the linear coupling part.

Before considering a concrete example, it is worthwhile to emphasize that, with these assumptions, and assuming that the internal complexities of the transistor can abstracted away into a local nonlinear response σ, the physical system follows an equation for which each numerical step resembles propagation through a residual layer:

$$x_i[t+1] = x_i[t] + \Delta t(f_i[t] + \sum_{j=1}^{N} a_{ij}[t] x_j[t] + \sigma(x_i[t])). \tag{45}$$

Simulating transistors in the RF, transient and nonlinear regime is significantly more challenging than just applying element-wise nonlinear activation functions. While PNNs could in principle make use of such detailed physics beyond Eqn. 45, it is difficult to estimate how much computational benefit such higher-order physics would confer. The main takeaway is just that, up to some approximations and abstractions, networks of coupled transistors (or other approximately-local nonlinear elements, like diodes) perform operations as they evolve that are very similar to those in conventional neural networks. Therefore, to this coarse level of detail, we feel relatively more confident in asserting that PNNs based on these systems may ultimately be able to approach their self-simulation quotient for tasks of practical interest, provided the self-simulation quotient is computed conservatively (i.e., that computational advantages for practical tasks could be as high as $\sim 1\%$ of the self-simulation quotients).

As an example, let us assume an electronic network of typical modern transistors. Modern CPUs/GPUs require 10 W per billion transistors ($\sim$ 100 nW per transistor, on average) [109], but in part this power requirement relies on efficient use of transistors such that all are not on all the time, so the power consumption per transistor during its operation is much higher. Clock rates of up to few GHz are typical. In typical digital usage, these transistors are operated in constrained safe-operation regimes. However, as analog devices, these same transistors are more complex, especially if driven outside these typical regimes, for high-frequency analog signals beyond the small-signal regime [110, 111]. We will therefore assume the transistors dissipate roughly 1000 nW each, and can be operated at digital clock cycles of a few GHz, such that their nonlinear analog dynamics can be as fast as 20 GHz or so. In addition, we will typically want to use digital-analog conversion for inputs. As is discussed in the last section, it is ideal if the majority of the circuit's parameters are stored in-place. We will therefore assume there are N_T simultaneous, time-dependent inputs to and outputs from the circuit, requiring N_T DAC and ADCs.

Let us assume $N_T = 100$ transistors described by a $N_d = 10$ equivalent circuit, an evolution time of 10 ns, 5 GHz bandwidth inputs (so that a maximum frequency of 20 GHz occurs in the simulation) and a coupling range of $M = 100$ neighbouring transistors. This leads to a physical energy cost of:

$$N_T \times P_{\text{transistor}} \times \Delta T = 100 \times 100 \text{ nW} \times 10 \text{ ns} = 1 \text{ pJ} \tag{46}$$

for the transistors themselves, and

$$N_T \times 2 \times \Delta T f_{\text{ s}} = 100 \times 10 \text{ ns} \times 1 \text{ pJ} \times 5 \text{ GHz} = 10 \text{ nJ} \tag{47}$$

for the DAC and ADC, where f_{s} is the input and output sampling frequency. Arguably, the transistor power requirement can be neglected, both because it is much smaller than any DAC/ADC cost, but also because the signals from the DAC provide the power required to drive the transistors. We will however not do this, since we are interested in order-of-magnitude estimates that are preferably conservative.

Meanwhile, simulations require:

$$N_{\text{ops}} \approx \Delta T \Delta f [N_T N_d M_1 + N_T N_d C_{\text{nl}}] = 10 \text{ ns} \times 20 \text{ GHz} \times [100 \times 10 \times 100 + 100 \times 10 \times 10] \approx 10^7 \tag{48}$$

As a result, the digital simulation, which consists effectively of locally-structured matrix-vector multiplications and 3rd-order tensor contractions, takes 0.1 mJ (0.4 mJ) and 310 ns (3.6 ns) on the GTX GPU (DGX Superpod).

From this, we see that a small ($N_T = 100$) analog transistor network has a self-simulation quotient of around 10^4 to 10^5 for energy, and between 0.4 and 30 for speed. To increase this advantage (and thus, speculatively, the computational capabilities of a PNN based on the analog transistor network), it is clear that establishing longer-range coupling M would be desirable.

We can increase the speed SSQ simply by increasing the number of transistors. For example, with $N_T = 10^3$, and $\Delta T = 1$ ns, the number of operations required for simulation is the same as the example above, and the total energy

required to run the network for ΔT is the same (since $N_T \Delta T$ was held constant). However, in this case the electronic circuit has a speed advantage of 310 (4) over the GTX GPU (DGX Superpod). Obviously there is a limit to how much this can be exploited, since the evolution time of the circuit cannot be made arbitrarily short if we require non-trivial physical transformations and long-range coupling. Nonetheless, this illustrates how adding parallel physical processes to a physical system can significantly enhance its potential computational power.

We could also increase the self-simulation quotients by adding additional "parameter" transistors that do not require fast input and output, and thus do not add to the DAC and ADC costs, but instead add only a smaller cost associated with providing enough total power to compensate for the dissipation of the additional transistors. If maintaining each additional such transistor requires an additional 1 µW, one could increase the self-simulation quotients by nearly 10^6 by adding 10^6 such transistors before the cost of these additional transistors was comparable to the DAC/ADC cost.

Finally, we note that the above discussion has neglected the details of realizing controllable linear coupling between transistors (or, in the earlier section, oscillators). Our findings should hold for coupling techniques that require no energy to maintain, such as those based on switchable bi- or multi-stable circuit elements, even if some energy is required to change their value (e.g., such as elements used in many memory devices). If constant power is required, however, its cost could be significant, since the number of connection elements is M_1 times larger than the number of transistors. If connections were implemented using transistors operated as voltage-controlled resistors, for example, the self-simulation quotients predicted above would be up to M_1 times smaller (for energy, for speed there would be no change). For very large transistor networks, this would not preclude enormous SSQs, since the SSQs found above can very easily exceed 10^{10} for realistic numbers of transistors and $M_1 \approx 10^3$. Nonetheless, passive, or nearly-passive, connectivity will be an important feature of scalable PNNs. In this regard, PAT and the PNN concept should be useful in using coupling mechanisms that do not necessarily exhibit ideal linearity, low noise, high-controllable-precision, or any other number of features that would be required if devices were assumed to *perfectly* obey an equation like Eqn. 45.

C. The operational bottleneck

Drawing on the previous subsections and other analysis we have performed in the preparation of this work, this section and the next one attempt to provide some heuristic guidelines for the types of PNNs that could provide real benefits for computation, and the conditions under which these might be achieved. Neither of these final sections is intended to be exhaustive, but merely to summarize some preliminary considerations.

In this section, for clarity we use the terminology PNN to refer specifically to the part of an overall neuromorphic hardware that is based on trained, unconventional physical evolutions. We consider the possibility that a more general-purpose analog hardware is used in tandem with this unconventional physical system, in addition to digital hardware; for clarity in this section, to delineate between the digital hardware, general-purpose analog hardware, and unconventional trained physics hardware, we will refer only the last hardware as PNN.

Virtually all tasks one could ask a neurmorphic processor to do will involve a number of read-in operations N_{RI} from a digital memory, and read-out operations N_{RO} back to the digital processor at the end of the computation. These operations account for converting the data from the electronic digital domain to the analog domain (either to the physical domain of the PNN, or a general-purpose analog hardware being used), and from the analog domain to the electronic digital domain. Of course, there will also be a third set of operations, those associated with actually performing the computation, which may be performed entirely or in part by the PNN system. If portions of the computation are performed with conventional digital hardware, those operations can be counted as N_{D}. If they are performed by general-purpose analog hardware, we will count them as N_{A}.

To quantify the *effective* number of operations performed by the physical system, $N_{\mathrm{P,eff}}$, we should compare the

PNN's performance to an equivalent algorithm executed on a conventional computer. For example, if a PNN performs MNIST classification with a certain accuracy, we would say it performs $N_{\mathrm{P,eff}} = N_{\mathrm{C}}$ operations, where N_{C} is the number of operations required by a digital NN that reaches the same performance. If the PNN uses some digital operations and requires some operations from general-purpose analog hardware, the physical portion's effective operations count is the remainder: $N_{\mathrm{P,eff}} = N_{\mathrm{C}} - N_{\mathrm{D}} - N_{\mathrm{A}}$.

For computational acceleration, we ultimately care about having the total time and energy of the computation (including all digital overhead operations) be significantly smaller than the equivalent time and energy of doing the calculation purely on a digital computer. If an unconventional physical system is to be useful, it should offer a significant energy and speed advantage over both conventional digital electronics, and state-of-the-art general-purpose analog hardware, and it should be able to perform a large enough fraction of routine computations that the net energy or time savings is significant.

We therefore want the following two statements to be true. First, to ensure an energy benefit from the PNN:

$$e_{\mathrm{RO}} N_{\mathrm{RO}} + e_{\mathrm{RI}} N_{\mathrm{RI}} + e_{\mathrm{D}} N_{\mathrm{D}} + e_{\mathrm{A}} N_{\mathrm{A}} + E_{\mathrm{PNN}} \ll e_{\mathrm{D}} N_{\mathrm{C}}, \tag{49}$$

where e_{RO}, e_{RI}, e_{D}, e_{A} are the per-operation energy cost of read-out, read-in, digital operations, operations by a general-purpose analog hardware, and E_{PNN} is the total energy cost of executing physical operations (i.e., the unconventional physical processor cost).

Second, to ensure a speed benefit from the PNN:

$$t_{\mathrm{RO}} N_{\mathrm{RO}} + t_{\mathrm{RI}} N_{\mathrm{RI}} + t_{\mathrm{D}} N_{\mathrm{D}} + t_{\mathrm{A}} N_{\mathrm{A}} + T_{\mathrm{PNN}} \ll t_{\mathrm{D}} N_{\mathrm{C}}, \tag{50}$$

where t_{RO}, t_{RI}, t_{D}, and t_{A} are the per-operation time to perform read-out, read-in, digital operations, general-purpose analog hardware operations, and T_{PNN} is the total time to execute physical operations.

To a first approximation, we can assume that all digital operations, including read-in and read-out, will take the same amount of energy and time, such that $e_{\mathrm{RO}} = e_{\mathrm{RI}} = e_{\mathrm{D}}$ and $t_{\mathrm{RO}} = t_{\mathrm{RI}} = t_{\mathrm{D}}$. The general-purpose analog hardware will likely be significantly faster and more energy-efficient. Thus, for maximum performance, one also should ideally optimize a calculation such that any operations not performed by unconventional PNN physics are primarily performed on a general-purpose analog processor rather than a digital one. To simplify this discussion, we will neglect this part of the operational optimization and focus only on the distribution of operations between the PNN and all other hardware.

Operational bottleneck: A task-PNN combination is operationally bottlenecked if the vast majority of the operations (the 'complexity' of the calculation task) occurs within the PNN, with only a small amount happening at the input (read-in), output (read-out), on digital hardware, or on general-purpose analog hardware. A task in which we read-in N numbers and find the maximum of those N numbers cannot be operationally bottlenecked, regardless of the choice of PNN, since the read-in consumes N operations and the computation only requires $\sim N$ digital-equivalent operations. A task in which N numbers are read-in and we need to perform the equivalent of $\sim N^2$ operations could in principle be operationally bottlenecked. This is true of matrix-vector multiplication, for example.

With our assumptions above in place, such an operational bottleneck means:

$$N_{\mathrm{RO}} + N_{\mathrm{RI}} + N_{\mathrm{D}} + N_{\mathrm{A}} \ll N_{\mathrm{C}}. \tag{51}$$

This is because the operations on the left hand side are assumed to cost significantly more than those of the PNN. Even if the energy and time required for executing the PNN physical system are both zero ($T_{\mathrm{PNN}} = E_{\mathrm{PNN}} = 0$ above), an unconventional physical processor can only provide a benefit to the extent the above inequality is satisfied.

The operational bottleneck condition is, of course, equivalent to saying that the vast majority of the computational operations need to be performed by the unconventional physical system, since that is where speed-ups and energy gains can be realized:

$$N_{\mathrm{P,eff}} \gg N_{\mathrm{RO}} + N_{\mathrm{RI}} + N_{\mathrm{D}} + N_{\mathrm{A}}. \tag{52}$$

For example, with MNIST handwritten digit classification, a 196-dimensional image is sent into the PNN, and the PNN produces a 10-D output, or possibly a 1-D output. If the PNN's performance is equivalent to a 196x10 single-layer perceptron (i.e., it gets about 90% accuracy), then the number of equivalent-operations it performs is only 10 times higher than the number of operations required for read-in. It is thus very hard for such a simple task to gain from a PNN: the PNN will always be far from being operationally bottlenecked, and will fail to offer much benefit, if any. Concretely, $N_{\mathrm{RO}} = 10$, $N_{\mathrm{RI}} = 196$, and $N_{\mathrm{D}} \sim 196$ (since we will almost always have to rescale data before sending it to a physical co-processor). Meanwhile, the total number of operations is at most 1960, so the maximum possible energy gain (and speed-up) possible with a physical co-processor is $1960/(10 + 196 + 196) \approx 5$. If the full 784-dimensional images are used, then the potential for speed-up may be even smaller.

If on the other hand, the processor is performing a much harder task, then it may be possible for the number of operations performed physically, with the PNN components, to dwarf the read-in and read-out operations, and any digital overhead operations. For CIFAR10, high accuracy classification of the 3-channel, 32 by 32 images would be equivalent to 1-100 billion digital operations [112]. The maximum possible PNN advantage is then approximately $\sim 10^9/(32 \times 32 \times 3) \approx 10^5$.

D. PNN design considerations for practical benefits

There are several ways to overcome these challenges, which are mostly not exclusive. Here, we briefly describe some strategies.

1. Parameter storage and interlayer connectivity

Parameter-in-place PNNs: If the PNN's parameters must be read-in for each inference (i.e., streamed parameters, Fig. S37a), then the number of read-in operations can easily approach the scale of the computational operations (unless the PNN somehow only requires a few parameters). Since read-in operations are limited by the energy and speed of conventional processors, it is optimal that most of a PNN's physical parameters are stored in-place, as in the right part of Fig. S37a. That is, the parameters that control the PNN's input-output transformation should ideally be stored in the PNN itself, rather than read-in through the same channel as the inputs. If computations the PNN performs are part of a typical NN inference, then the parameters do not need to be updated as often as the inputs, and the PNN can avoid a significant read-in cost. As was noted in the last section, it is helpful (but not essential) that in-place parameters be fully passive, in order to minimize their energy cost. Examples of in-place parameters typically overlap with devices or effects that have been used or proposed as memories, such as phase-change materials or liquid crystals, multi-stable MEMs devices as in digital micromirror devices, or multistable circuits like flip-flops.

Physical-feedforward PNNs: In some PNNs, the outputs can be fed directly into another PNN layer without a digital intermediary (Fig. S37b) between the physical layers. PNNs in which the output is directly measured (such as electronic PNNs where the output is a voltage, or possibly in optical cavities or arrangements, where the full optical field is fed-forward to subsequent physical processing) are in this category, as are PNNs which have a memory of previous inputs and states (as in optical cavities or other resonators). Physical-feedforward PNNs thus include

recurrent PNNs, but recurrence is not necessary for physical feed-forward: for example, in a sequence of several electronic circuits, the voltage from each circuit can be directly routed to the next, but in a fully feedforward fashion without any need for a hidden state memory. With physical-feedforward, one can cascade PNNs over many layers, building up computational complexity, without incurring read-in or read-out costs except at the very beginning and end.

2. Pre-training PNNs using in silico training

Although *in silico* training will often suffer from simulation-reality gaps, it can be complementary to physics-aware training, as well as to techniques for physical learning hardware. As context, in modern deep learning, large models are typically pretrained for a class of tasks, and then are adapted to new, related tasks and data by training only a subpart of the model (e.g., the final layer or layers) on the new data/task, while keeping other parts (e.g., the initial layers) fixed [113]. *In silico* training and PAT are well-suited to efficiently implementing the fixed, pretrained parts of such models as PNNs, while techniques for learning hardware [7, 40–44, 46, 47, 81, 114–116] may be ideal for implementing the retrainable parts.

In silico training can be used to train many physical parameters. Importantly, this includes physical parameters that can be set permanently when a device is fabricated. Since reconfigurable parameters are expensive, pretraining a device *in silico* may allow for the fabricated device to be able to learn a variety of tasks with relatively fewer reconfigurable parameters, since many non-reconfigurable parameters could be trained prior to fabrication *in silico*. Physics-aware training can be used to train this relatively small number of reconfigurable parameters (including both pre-and post-processing parameters, as well as physical reconfigurable parameters) to recover performance expected from simulations, as well as to transfer to new tasks. Alternatively, conventional digital electronic hardware or efficient analog learning hardware [7, 40–44, 46, 47, 81, 114–116] might be used to implement the plastic parts of an overall neuromorphic hardware instead. In this context, such digital or analog learning hardware could facilitate transfer to new tasks, without needing to adjust the pretrained physical hardware again, so that PAT would only need to be used to used once correct for the simulation-reality gap in the subsequently-fixed, pretrained parts of the physical hardware. As examples, such pretrained PNNs could be used to implement a large fraction of the initial layers of a large image-classification or speech-classification network, generating mainly generic features [117], or could emulate pretrained Transformers [118].

3. PNNs for physical-domain inputs and outputs

There are two other important scenarios where PNNs can have benefits, but that are broader than just computational energy-efficiency or speed. In these contexts, PAT could be used to apply differential programming [119–121] to the design of complex functional devices. While the use of differential programming for inverse design of functional devices within simulations is not new (e.g., [25–27, 122–124]), its applicability is limited by simulation-reality gaps, which PAT could help close.

Physical-data-domain-*input* PNNs: In some PNNs, the input data will already be in the physical domain of the PNN, such as light entering an optical PNN. In this case, the PNN not only avoids the read-in cost, but can additionally perform operations that a traditional device, which would first read-in the data to the electronic domain and then process it, could not. As in compressed-sensing single-pixel cameras using spatial light modulators [125], this could allow for higher-speed, higher-resolution, or lower-energy costs for performing measurements (either by improving the nature of the measurement itself, or by performing parts of the post-processing physically). In other words, one could use PAT to train physical 'smart sensors' [30–32] (Fig. S37d).

Physical-*output* PNNs: PNNs may also provide a means of producing a desired physical quantity or functionality, in the domain of the physical system, rather than a computation result that is ultimately utilized in a downstream digital computer. In PNNs, the final output is normally obtained from measuring the physical system. However, we can instead treat the final state of the physical system as the output, and the measurement of it as merely an assessment of that physical state. For example, a nonlinear optical PNN may be trained to produce a desired output pulse, which would be assessed based on measurements of that pulse (such as the power spectrum used in our optical SHG-PNN). Alternatively, the physical functionality of the produced physical state might be assessed, such as by measuring the effects of the produced pulse on a chemical, the optical functionality of a complex nanostructure [27, 28, 126], material functionality [18, 29], or the behavior of small robots [24–26, 123, 124]. In all cases, measurements that assess the physical state need to be performed and transferred to the digital domain, in order to facilitate PAT, but the true output of the PNN is the physical object and its state. Thus, in PNNs of this kind, as with the 'smart sensors' described above, PAT allows the backpropagation algorithm for gradient descent to be applied to produce a desired physical state or functionality, in the physical domain of the PNN (Fig. S37e). This mode of use has some resemblance to deep reinforcement learning. In PAT, the structure of the optimization of physical functionality is model-based, since the differentiable digital model is explicitly an estimator of the physical system's derivatives. It will nonetheless be interesting to explore variants of PAT that adopt some reinforcement learning techniques, especially in scenarios where the digital model's accuracy is low, or where there may be specific features of the physical system that may be exploited to assist learning.

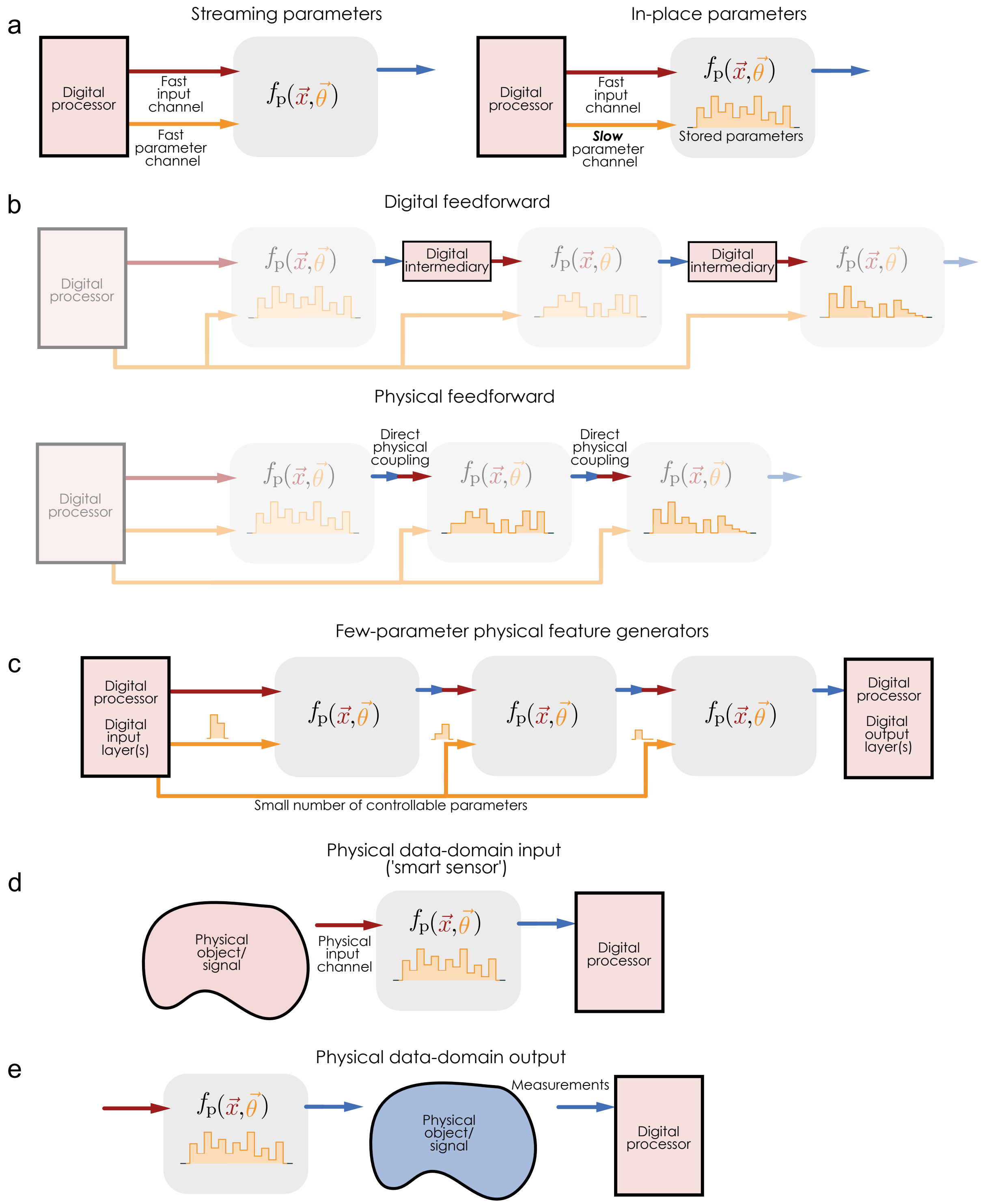

Fig. S37: Various types of PNNs and PNN design considerations, with emphasis drawn to designs that may facilitate practical benefits. **a.** Streaming-parameter versus in-place parameter PNNs. **b.** Physical-feedforward versus digital feedforward PNNs. Parts of the diagrams that are common between the two operation regimes have been faded out in order to emphasize the key differences. **c.** PNNs with a small number of reconfigurable parameters. **d.** Physical data-domain input PNNs. **e.** Physical data-domain output PNNs. Note that where a digital processor is shown, a general-purpose analog hardware could be substituted; this would usually be preferred given that most digital operations here are spent executing conventional feedforward neural networks, which could be efficiently executed by analog hardware. Although using an analog hardware for these operations would not completely eliminate the need for digital-to-analog conversion and analog-to-digital conversion, it might reduce the number of these operations required.

[1] Patterson, D. et al. Carbon emissions and large neural network training. *arXiv:2104.10350* (2021).

[2] Reuther, A. et al. Survey of machine learning accelerators. In *2020 IEEE High Performance Extreme Computing Conference (HPEC)*, 1–12 (IEEE, 2020).

[3] Xia, Q. & Yang, J. J. Memristive crossbar arrays for brain-inspired computing. *Nature materials* **18**, 309–323 (2019).

[4] Burr, G. W. et al. Neuromorphic computing using non-volatile memory. *Advances in Physics: X* **2**, 89–124 (2017).

[5] Khaddam-Aljameh, R. et al. HERMES core–a 14nm CMOS and PCM-based in-memory compute core using an array of 300ps/LSB linearized CCO-based ADCs and local digital processing. In *2021 Symposium on VLSI Circuits*, 1–2 (IEEE, 2021).

[6] Narayanan, P. et al. Fully on-chip mac at 14nm enabled by accurate row-wise programming of PCM-based weights and parallel vector-transport in duration-format. In *2021 Symposium on VLSI Technology*, 1–2 (IEEE, 2021).

[7] Kohda, Y. et al. Unassisted true analog neural network training chip. In *2020 IEEE International Electron Devices Meeting (IEDM)*, 36–2 (IEEE, 2020).

[8] Marković, D., Mizrahi, A., Querlioz, D. & Grollier, J. Physics for neuromorphic computing. *Nature Reviews Physics* **2**, 499–510 (2020).

[9] Wetzstein, G. et al. Inference in artificial intelligence with deep optics and photonics. *Nature* **588**, 39–47 (2020).

[10] Romera, M. et al. Vowel recognition with four coupled spin-torque nano-oscillators. *Nature* **563**, 230–234 (2018).

[11] Shen, Y. et al. Deep learning with coherent nanophotonic circuits. *Nature Photonics* **11**, 441 (2017).

[12] Prezioso, M. et al. Training and operation of an integrated neuromorphic network based on metal-oxide memristors. *Nature* **521**, 61–64 (2015).

[13] Euler, H.-C. R. et al. A deep-learning approach to realizing functionality in nanoelectronic devices. *Nature Nanotechnology* **15**, 992–998 (2020).

[14] Hughes, T. W., Williamson, I. A., Minkov, M. & Fan, S. Wave physics as an analog recurrent neural network. *Science Advances* **5**, eaay6946 (2019).

[15] Wu, Z., Zhou, M., Khoram, E., Liu, B. & Yu, Z. Neuromorphic metasurface. *Photonics Research* **8**, 46 (2020).

[16] Furuhata, G., Niiyama, T. & Sunada, S. Physical deep learning based on optimal control of dynamical systems. *Physical Review Applied* **15**, 034092 (2021).

[17] Lin, X. et al. All-optical machine learning using diffractive deep neural networks. *Science* **361**, 1004–1008 (2018).

[18] Miller, J. F., Harding, S. L. & Tufte, G. Evolution-in-materio: evolving computation in materials. *Evolutionary Intelligence* **7**, 49–67 (2014).

[19] Chen, T. et al. Classification with a disordered dopant-atom network in silicon. *Nature* **577**, 341–345 (2020).

[20] Bueno, J. et al. Reinforcement learning in a large-scale photonic recurrent neural network. *Optica* **5**, 756–760 (2018).

[21] Tanaka, G. et al. Recent advances in physical reservoir computing: a review. *Neural Networks* **115**, 100–123 (2019).

[22] Appeltant, L. et al. Information processing using a single dynamical node as complex system. *Nature Communications* **2**, 1–6 (2011).

[23] Mouret, J.-B. & Chatzilygeroudis, K. 20 years of reality gap: a few thoughts about simulators in evolutionary robotics. In *Proceedings of the Genetic and Evolutionary Computation Conference Companion*, 1121–1124 (2017).

[24] Howison, T., Hauser, S., Hughes, J. & Iida, F. Reality-assisted evolution of soft robots through large-scale physical experimentation: a review. *Artificial Life* **26**, 484–506 (2021).

[25] de Avila Belbute-Peres, F., Smith, K., Allen, K., Tenenbaum, J. & Kolter, J. Z. End-to-end differentiable physics for learning and control. *Advances in Neural Information Processing Systems* **31**, 7178–7189 (2018).

[26] Degrave, J., Hermans, M., Dambre, J. et al. A differentiable physics engine for deep learning in robotics. *Frontiers in neurorobotics* **13**, 6 (2019).

[27] Molesky, S. et al. Inverse design in nanophotonics. *Nature Photonics* **12**, 659–670 (2018).

[28] Peurifoy, J. et al. Nanophotonic particle simulation and inverse design using artificial neural networks. *Science Advances* **4**, eaar4206 (2018).

[29] Stern, M., Arinze, C., Perez, L., Palmer, S. E. & Murugan, A. Supervised learning through physical changes in a mechanical system. *Proceedings of the National Academy of Sciences* **117**, 14843–14850 (2020).

[30] Zhou, F. & Chai, Y. Near-sensor and in-sensor computing. *Nature Electronics* **3**, 664–671 (2020).

[31] Martel, J. N., Mueller, L. K., Carey, S. J., Dudek, P. & Wetzstein, G. Neural sensors: Learning pixel exposures for HDR imaging and video compressive sensing with programmable sensors. *IEEE Transactions on Pattern Analysis and Machine Intelligence* **42**, 1642–1653 (2020).

[32] Mennel, L. et al. Ultrafast machine vision with 2D material neural network image sensors. *Nature* **579**, 62–66 (2020).

[33] Brooks, R. A. Intelligence without reason. In *Proceedings of the 12th International Joint Conference on Artificial Intelligence - Volume 1*, 569–595 (Morgan Kaufmann Publishers, 1991).

[34] Hooker, S. The hardware lottery. *arXiv:2009.06489* (2020).

[35] Krizhevsky, A., Sutskever, I. & Hinton, G. E. Imagenet classification with deep convolutional neural networks. *Advances in Neural Information Processing Systems* **25**, 1097–1105 (2012).

[36] Lin, H. W., Tegmark, M. & Rolnick, D. Why does deep and cheap learning work so well? *Journal of Statistical Physics* **168**, 1223–1247 (2017).

[37] Grollier, J. et al. Neuromorphic spintronics. *Nature Electronics* **3**, 360–370 (2020).

[38] Mitarai, K., Negoro, M., Kitagawa, M. & Fujii, K. Quantum circuit learning. *Physical Review A* **98**, 032309 (2018).

[39] Poggio, T., Banburski, A. & Liao, Q. Theoretical issues in deep networks. *Proceedings of the National Academy of Sciences* **117**, 30039–30045 (2020).

[40] Scellier, B. & Bengio, Y. Equilibrium propagation: Bridging the gap between energy-based models and backpropagation. *Frontiers in Computational Neuroscience* **11**, 24 (2017).

[41] Ernoult, M., Grollier, J., Querlioz, D., Bengio, Y. & Scellier, B. Equilibrium propagation with continual weight updates. *arXiv:2005.04168* (2020).

[42] Laborieux, A. et al. Scaling equilibrium propagation to deep convnets by drastically reducing its gradient estimator bias. *Frontiers in Neuroscience* **15**, 129 (2021).

[43] Martin, E. et al. Eqspike: spike-driven equilibrium propagation for neuromorphic implementations. *iScience* **24**, 102222 (2021).

[44] Dillavou, S., Stern, M., Liu, A. J. & Durian, D. J. Demonstration of decentralized, physics-driven learning. *arXiv:2108.00275* (2021).

[45] Hermans, M., Burm, M., Van Vaerenbergh, T., Dambre, J. & Bienstman, P. Trainable hardware for dynamical computing using error backpropagation through physical media. *Nature Communications* **6**, 1–8 (2015).

[46] Hughes, T. W., Minkov, M., Shi, Y. & Fan, S. Training of photonic neural networks through in situ backpropagation and gradient measurement. *Optica* **5**, 864–871 (2018).

[47] Lopez-Pastor, V. & Marquardt, F. Self-learning machines based on hamiltonian echo backpropagation. *arXiv:2103.04992* (2021).

[48] Hubara, I., Courbariaux, M., Soudry, D., El-Yaniv, R. & Bengio, Y. Quantized neural networks: Training neural networks with low precision weights and activations. *The Journal of Machine Learning Research* **18**, 6869–6898 (2017).

[49] Frye, R. C., Rietman, E. A. & Wong, C. C. Back-propagation learning and nonidealities in analog neural network hardware. *IEEE Transactions on Neural Networks* **2**, 110–117 (1991).

[50] Cramer, B. et al. Surrogate gradients for analog neuromorphic computing. *arXiv:2006.07239* (2020).

[51] Adhikari, S. P., Yang, C., Kim, H. & Chua, L. O. Memristor bridge synapse-based neural network and its learning. *IEEE Transactions on Neural Networks and Learning Systems* **23**, 1426–1435 (2012).

[52] Lillicrap, T. P., Cownden, D., Tweed, D. B. & Akerman, C. J. Random synaptic feedback weights support error backpropagation for deep learning. *Nature Communications* **7**, 1–10 (2016).

[53] Launay, J., Poli, I., Boniface, F. & Krzakala, F. Direct feedback alignment scales to modern deep learning tasks and architectures. *arXiv:2006.12878* (2020).

[54] Paszke, A. et al. PyTorch: An imperative style, high-performance deep learning library. In *Advances in Neural Information Processing Systems*, 8024–8035 (2019).

[55] LeCun, Y., Bottou, L., Bengio, Y. & Haffner, P. Gradient-based learning applied to document recognition. *Proceedings of the IEEE* **86**, 2278–2324 (1998).

[56] Xiao, H., Rasul, K. & Vollgraf, R. Fashion-MNIST: a novel image dataset for benchmarking machine learning algorithms. *arXiv:1708.07747* (2017).

[57] Spoon, K. et al. Toward software-equivalent accuracy on transformer-based deep neural networks with analog memory devices. *Frontiers in Computational Neuroscience* 53 (2021).

[58] Kariyappa, S. et al. Noise-resilient DNN: Tolerating noise in PCM-based AI accelerators via noise-aware training. *IEEE Transactions on Electron Devices* (2021).

[59] Gokmen, T., Rasch, M. J. & Haensch, W. The marriage of training and inference for scaled deep learning analog hardware. In *2019 IEEE International Electron Devices Meeting (IEDM)*, 22–3 (IEEE, 2019).

[60] Rasch, M. J. et al. A flexible and fast PyTorch toolkit for simulating training and inference on analog crossbar arrays. In *2021 IEEE 3rd International Conference on Artificial Intelligence Circuits and Systems (AICAS)*, 1–4 (IEEE, 2021).

[61] Falcon, W. et al. PyTorch Lightning, `https://github.com/PyTorchLightning/pytorch-lightning` (2019).

[62] Biewald, L. Experiment Tracking with Weights and Biases, `https://www.wandb.com/` (2020).

[63] Kasim, M. F. et al. Up to two billion times acceleration of scientific simulations with deep neural architecture search. *arXiv:2001.08055* (2020).

[64] Rahmani, B. et al. Actor neural networks for the robust control of partially measured nonlinear systems showcased for image propagation through diffuse media. *Nature Machine Intelligence* **2**, 403–410 (2020).

[65] Karniadakis, G. E. et al. Physics-informed machine learning. *Nature Reviews Physics* **3**, 422–440 (2021).

[66] Akiba, T., Sano, S., Yanase, T., Ohta, T. & Koyama, M. Optuna: A next-generation hyperparameter optimization framework. In *Proceedings of the 25th ACM SIGKDD International Conference on Knowledge Discovery & Data Mining*, 2623–2631 (2019).

[67] Liu, W. et al. Programmable controlled mode-locked fiber laser using a digital micromirror device. *Optics Letters* **42**, 1923–1926 (2017).

[68] Matthès, M. W., del Hougne, P., de Rosny, J., Lerosey, G. & Popoff, S. M. Optical complex media as universal reconfigurable linear operators. *Optica* **6**, 465–472 (2019).

[69] Popoff, S. M. & Matthès, M. W. ALP4lib: A Python wrapper for the Vialux ALP-4 controller suite to control DMDs, `https://doi.org/10.5281/zenodo.4076193` (2020).

[70] Hillenbrand, J. M. Vowel formant frequency classification data, `https://homepages.wmich.edu/~hillenbr/voweldata.html`.

[71] Veit, A., Wilber, M. & Belongie, S. Residual networks behave like ensembles of relatively shallow networks. *arXiv:1605.06431* (2016).

[72] Jakobi, N., Husbands, P. & Harvey, I. Noise and the reality gap: The use of simulation in evolutionary robotics. In *European Conference on Artificial Life*, 704–720 (Springer, 1995).

[73] Nielsen, M. *Neural Networks and Deep Learning* (Determination Press, 2015).

[74] Kingma, D. P. & Ba, J. Adam: A method for stochastic optimization. In *International Conference on Learning Representations* (2015).

[75] Zeiler, M. D. Adadelta: An adaptive learning rate method. *arXiv:1212.5701* (2012).

[76] Torrejon, J. et al. Neuromorphic computing with nanoscale spintronic oscillators. *Nature* **547**, 428–431 (2017).

[77] Nakajima, K. Physical reservoir computing—an introductory perspective. *Japanese Journal of Applied Physics* **59**, 060501 (2020).

[78] Fujii, K. & Nakajima, K. Harnessing disordered-ensemble quantum dynamics for machine learning. *Physical Review Applied* **8**, 024030 (2017).

[79] Brunner, D. et al. Tutorial: Photonic neural networks in delay systems. *Journal of Applied Physics* **124**, 152004 (2018).

[80] Larger, L. et al. High-speed photonic reservoir computing using a time-delay-based architecture: Million words per second classification. *Physical Review X* **7**, 011015 (2017).

[81] Hermans, M., Soriano, M. C., Dambre, J., Bienstman, P. & Fischer, I. Photonic delay systems as machine learning implementations. *Journal of Machine Learning Research* **16**, 2081–2097 (2015).

[82] Rafayelyan, M., Dong, J., Tan, Y., Krzakala, F. & Gigan, S. Large-scale optical reservoir computing for spatiotemporal chaotic systems prediction. *Physical Review X* **10**, 041037 (2020).

[83] Ballarini, D. et al. Polaritonic neuromorphic computing outperforms linear classifiers. *Nano Letters* **20**, 3506–3512 (2020).

[84] Gallicchio, C., Micheli, A. & Pedrelli, L. Deep reservoir computing: A critical experimental analysis. *Neurocomputing* **268**, 87–99 (2017).

[85] Braun, O. M. & Kivshar, Y. S. Nonlinear dynamics of the frenkel–kontorova model. *Physics Reports* **306**, 1–108 (1998).

[86] Chen, R. T., Rubanova, Y., Bettencourt, J. & Duvenaud, D. K. Neural ordinary differential equations. In *Advances in Neural Information Processing Systems*, 6571–6583 (2018).

[87] Feynman, R. P. Simulating physics with computers. *Int. J. Theor. Phys* **21** (1982).

[88] Arute, F. et al. Quantum supremacy using a programmable superconducting processor. *Nature* **574**, 505–510 (2019).

[89] Zhong, H.-S. et al. Quantum computational advantage using photons. *Science* **370**, 1460–1463 (2020).

[90] He, K., Zhang, X., Ren, S. & Sun, J. Deep residual learning for image recognition. In *Proceedings of the IEEE Conference on Computer Vision and Pattern Recognition*, 770–778 (2016).

[91] Green500 November 2020, `https://www.top500.org/lists/green500/2020/11/` (2020).

[92] GeForce 20 series - Wikipedia, `https://en.wikipedia.org/wiki/GeForce_20_series` (2021).

[93] Agrawal, G. P. *Nonlinear Fiber Optics* (Elsevier, 2000).

[94] Carlson, D. R. et al. Ultrafast electro-optic light with subcycle control. *Science* **361**, 1358–1363 (2018).

[95] Jankowski, M. et al. Ultrabroadband nonlinear optics in nanophotonic periodically poled lithium niobate waveguides. *Optica* **7**, 40–46 (2020).

[96] Xu, B., Zhou, Y. & Chiu, Y. A 23mW 24GS/s 6b time-interleaved hybrid two-step ADC in 28nm CMOS. In *2016 IEEE Symposium on VLSI Circuits*, 1–2 (IEEE, 2016).

[97] Sun, C. et al. Single-chip microprocessor that communicates directly using light. *Nature* **528**, 534–538 (2015).

[98] Atabaki, A. H. et al. Integrating photonics with silicon nanoelectronics for the next generation of systems on a chip. *Nature* **556**, 349–354 (2018).

[99] Wang, C. et al. Integrated lithium niobate electro-optic modulators operating at CMOS-compatible voltages. *Nature* **562**, 101–104 (2018).

[100] Koeber, S. et al. Femtojoule electro-optic modulation using a silicon–organic hybrid device. *Light: Science & Applications* **4**, e255–e255 (2015).

[101] Srinivasan, S. A. et al. 56 Gb/s germanium waveguide electro-absorption modulator. *Journal of Lightwave Technology* **34**, 419–424 (2016).

[102] Nozaki, K. et al. Photonic-crystal nano-photodetector with ultrasmall capacitance for on-chip light-to-voltage conversion without an amplifier. *Optica* **3**, 483–492 (2016).

[103] Tang, L. et al. Nanometre-scale germanium photodetector enhanced by a near-infrared dipole antenna. *Nature Photonics* **2**, 226–229 (2008).

[104] Nakajima, M., Tanaka, K. & Hashimoto, T. Neural Schrödinger equation: physical law as neural network. *arXiv:2006.13541* (2020).

[105] Teğin, U., Yıldırım, M., Oğuz, İ., Moser, C. & Psaltis, D. Scalable optical learning operator. *Nature Computational Science* **1**, 542–549 (2021).

[106] Günther, M., Feldmann, U. & ter Maten, J. Modelling and discretization of circuit problems. *Handbook of Numerical Analysis* **13**, 523–659 (2005).

[107] Rewieński, M. A perspective on fast-spice simulation technology. In *Simulation and Verification of Electronic and Biological Systems*, 23–42 (Springer, 2011).

[108] Benk, J., Denk, G. & Waldherr, K. A holistic fast and parallel approach for accurate transient simulations of analog circuits. *Journal of Mathematics in Industry* **7**, 1–19 (2017).

[109] Sun, Y., Agostini, N. B., Dong, S. & Kaeli, D. Summarizing CPU and GPU design trends with product data. *arXiv:1911.11313* (2019).

[110] Enz, C. C., Krummenacher, F. & Vittoz, E. A. An analytical MOS transistor model valid in all regions of operation and dedicated to low-voltage and low-current applications. *Analog Integrated Circuits and Signal Processing* **8**, 83–114 (1995).

[111] Enz, C. C. An MOS transistor model for RF IC design valid in all regions of operation. *IEEE Transactions on Microwave Theory and Techniques* **50**, 342–359 (2002).

[112] Hasanpour, S. H., Rouhani, M., Fayyaz, M. & Sabokrou, M. Lets keep it simple, using simple architectures to outperform deeper and more complex architectures. *arXiv:1608.06037* (2016).

[113] Goodfellow, I., Bengio, Y. & Courville, A. *Deep Learning* (MIT Press, 2016).

[114] Scellier, B. A deep learning theory for neural networks grounded in physics. *arXiv:2103.09985* (2021).

[115] Gokmen, T. & Haensch, W. Algorithm for training neural networks on resistive device arrays. *Frontiers in Neuroscience* **14**, 103 (2020).

[116] Gokmen, T. Enabling training of neural networks on noisy hardware. *Frontiers in Artificial Intelligence* 126 (2021).

[117] Sharif Razavian, A., Azizpour, H., Sullivan, J. & Carlsson, S. CNN features off-the-shelf: an astounding baseline for recognition. In *Proceedings of the IEEE Conference on Computer Vision and Pattern Recognition Workshops*, 806–813 (2014).

[118] Shen, S. et al. Reservoir transformer. *arXiv:2012.15045* (2020).

[119] Differentiable Programming - Wikipedia, `https://en.wikipedia.org/wiki/Differentiable_programming` (2021).

[120] Wang, F., Decker, J., Wu, X., Essertel, G. & Rompf, T. Backpropagation with callbacks: Foundations for efficient and expressive differentiable programming. *Advances in Neural Information Processing Systems* **31**, 10180–10191 (2018).

[121] Innes, M. et al. A differentiable programming system to bridge machine learning and scientific computing. *arXiv:1907.07587* (2019).

[122] Giles, M. B. & Pierce, N. A. An introduction to the adjoint approach to design. *Flow, Turbulence and Combustion* **65**, 393–415 (2000).

[123] Hermans, M., Schrauwen, B., Bienstman, P. & Dambre, J. Automated design of complex dynamic systems. *PLOS One* **9**, e86696 (2014).

[124] Hu, Y. et al. Difftaichi: Differentiable programming for physical simulation. *arXiv:1910.00935* (2019).

[125] Duarte, M. F. et al. Single-pixel imaging via compressive sampling. *IEEE Signal Processing Magazine* **25**, 83–91 (2008).

[126] Estakhri, N. M., Edwards, B. & Engheta, N. Inverse-designed metastructures that solve equations. *Science* **363**, 1333–1338 (2019).
